# Synthesis and Effect of the Structure of Bithienyl-Terminated Surfactants for Dielectric Layer Modification in Organic Transistor

**DOI:** 10.3390/ma14216345

**Published:** 2021-10-23

**Authors:** Lucia Feriancová, Iveta Kmentová, Michal Micjan, Milan Pavúk, Martin Weis, Martin Putala

**Affiliations:** 1 Department of Organic Chemistry, Faculty of Natural Sciences, Comenius University in Bratislava, Ilkovičova 6, 842 15 Bratislava, Slovakia; feriancova13@uniba.sk (L.F.); iveta.kmentova@uniba.sk (I.K.); 2 Institute of Electronics and Photonics, Slovak University of Technology, Ilkovičova 3, 812 19 Bratislava, Slovakia; michal.micjan@stuba.sk; 3 Institute of Nuclear and Physical Engineering, Slovak University of Technology, Ilkovičova 3, 812 19 Bratislava, Slovakia; milan.pavuk@stuba.sk

**Keywords:** bithienyl, chlorodimethylsilane, OFET, pentacene, phosphonic acid, self-assembled monolayer, sexithiophene, surface modification

## Abstract

A series of bithienyl-terminated surfactants with various alkyl chain lengths (from C8 to C13) and phosphono or chlorodimethylsilyl anchoring groups were synthesized by palladium-catalyzed hydrophosphonation, or platinum-catalyzed hydrosilylation as a key step. Surfactants were tested in pentacene or α-sexithiophene-based organic field-effect transistors (OFETs) for the modification of the dielectric surface. The studied surfactants increased the effective mobility of the α-sexithiophene-based device by up to one order of magnitude. The length of alkyl chain showed to be significant for the pentacene-based device, as the effective mobility only increased in the case of dielectric modification with bithienylundecylphosphonic acid. AFM allowed a better understanding of the morphology of semiconductors on bare SiO_2_ and surfaces treated with bithienylundecylphosphonic acid.

## 1. Introduction

In the field of organic electronics, increased attention is paid to organic semiconductor interfaces. Organic semiconducting materials (OS) are used as the active layer in many electronic applications, such as organic-field effect transistors (OFET), organic photovoltaic cells, organic light-emitting diodes or memory devices [1].

An organic field-effect transistor (OFET) efficiency is given mainly by the first two layers of the organic semiconductor at the dielectric surface where the charge transfer takes place [2]. Therefore, the quality of the interface between OS and insulator has a crucial role [3,4,5,6]. It should be mentioned here that this complex topic includes the impact of various effects. Charge carriers trapping at the organic semiconductor/gate dielectric interface due to the polaron nature of the dielectric surface, the presence of defects and chemical groups of the dielectric surface, and structural defects in the organic semiconductor, or the presence of adsorbed water molecules. In detail, the random orientation of the dipole moments of the gate dielectric surface leads to a significant broadening of the density of states in an organic semiconductor (dipolar electrostatic disorder). Gate dielectric surface with electroactive sites (e.g., hydroxy groups) acts as traps and suppress electron transport. Water molecules adsorbed on the gate dielectric surface also induce charge trapping in p-type organic semiconductors and cause voltage bias-stress instability.

**Figure 1:**
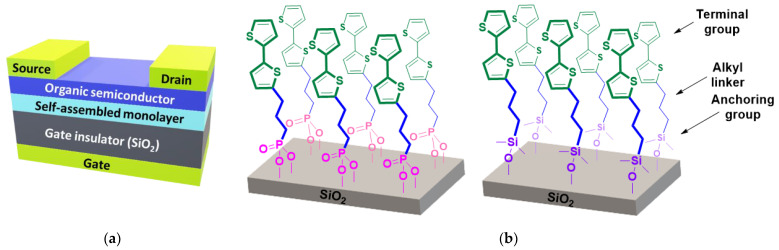
(**a**) Schematic view of the organic field-effect transistors (OFET) using the active layer of OS deposited on a SAM-modified gate insulator layer; (**b**) proposed modification of SiO_2_ surface by SAM formation with phosphonic acid and silane anchoring group.

Improvement in the electrical response can be achieved [1,7,8,9] (increasing mobility or reducing the threshold voltage) by modification of electronic and morphological properties of the interface, such as surface functionalization using specially designed organic molecules-surfactants forming a self-assembled monolayer (SAM, Figure 1a) [10]. The surfactant can also improve the wettability of the surface, providing new functionality to the substrate and can affect the structural order of organic semiconductor by directing the growth of semiconductor to obtain the suitable orientation for high FET performance [11,12,13,14,15,16].

The typical structure of such surfactants consists of α,ω-functionalized alkanes with an anchoring group at one end and terminal group at the other end (Figure 1b). The anchoring group is responsible for binding to the dielectric surface. The aliphatic chain contributes to the self-assembling process through interchain van der Waals interactions. The role of the terminal group is either to provide stronger interaction to the OS [9] or to act as an organic semiconductor in monolayer OFET devices [17].

While developing organic semiconductors with bithienyl substituted naphthalenes in our research group [18,19,20] we found problems with their deposition on the substrate during application in OFET due to low surface wettability, even in the case of the surface processed with commercially available octadecylsilyl trichloride (OTCS). Therefore, we designed surfactants containing bithienyl terminal groups to provide π–π interaction with our semiconducting materials. For interface modification on SiO_2_, SAMs with silane and phosphonic acid anchoring groups have been widely studied [9,17,21,22]. Since several types of silane anchoring groups, such as tri-, di-, and monochlorosilanes, or trimetoxysilanes, are used in SAM materials and are differing in reactivity, we have chosen the medium moisture-sensitive and medium reactive monochlorosilane group. Phosphono anchoring group was chosen as moisture insensitive, which significantly simplifies their storage and working conditions and does not undergo self-condensation. The designed surfactants were aimed to be tested with two typical p-type organic semiconductors—pentacene or sexithiophene in OFET.

**Figure 2:**
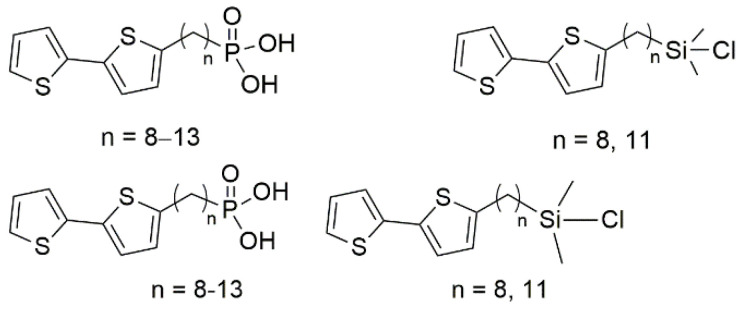
Chemical structures of designed derivatives with phosphono and chlorodimethylsilyl anchoring groups.

The chemical structure of the designed derivatives for the SAM formation is displayed in Figure 2. Each derivative consists of bithienyl terminal group, C8 to C13 alkyl linker and phosphono or chlorodimethylsilyl anchoring group. The bithienyl group should provide π–π interaction between neighboring molecules in both steps for possible guidance in controlled surface modification and later on for controlled self-assembling of selected semiconducting material on the modified surface. Due to the observed significant odd-even effect of aliphatic linker lengths on molecular packing of SAM [23,24], we selected structures with both even and odd numbers of carbon atoms in the alkyl chain.

## 2. Materials and Methods

### 2.1. Materials

Solvents were purified by standard methods before use [25]. The 4,4,5,5-tetramethyl-1,3,2-dioxaphospholane 2-oxide was purchased from TCI Chemicals (TCI Europe N. V.- Tokyo Chemical Industry, Brussels, Belgium). All bromoalkenes, 1,8-dibromooctane, bithiophene, and tetrakis(triphenylphosphine)palladium (0) (Pd(PPh_3_)_4_) were purchased from Fluorochem. *n*-Butyllithium (1.6 M solution in hexane), Karstedt′s catalyst and chlorodimethylsilane were purchased from Sigma Aldrich (Merck, KGaA, Darmstadt, Germany), and bromotrimethylsilane was purchased from Acros Organics (Thermo Fisher Scientific, Geel, Belgium). All chemicals were used as purchased without further purification. Thin-layer chromatography was performed on Merck TLC plates of silica gel 60, F-254, visualization under UV 254 nm and 365 nm, for column chromatography was used silica gel 60 (Merck).

### 2.2. Experiment

#### 2.2.1. Synthesis of ω-Bromoalk-1-enes (**1e**–**f**)

To a combined solution of LiCl (254 mg, 6 mmol, 12 mL THF) and CuCl_2_ (403 mg, 3 mmol, 12 mL THF), which was stirred overnight, 1,8-dibromooctane (4.08 g, 15 mmol) was added in one portion. The reaction mixture was cooled to 0 °C, and but-3-en-1-ylmagnesium bromide (2.43 g, 18 mmol, 10 mL of 2M solution in Et_2_O) was added through a cannula. After addition, the mixture was stirred overnight at room temperature. Then, 1 M HCl was added, and the mixture was stirred for another 30 min. The aqueous layer was extracted with diethyl ether. Combined organic layers were washed with brine, dried over Na_2_SO_4_, and the solvent was evaporated. The crude mixture was separated by flash chromatography (SiO_2_, hexanes). Further characterizations are reported in Supplementary Materials.

#### 2.2.2. General Procedure for Preparation of 5-(Alk-ω-en-1-yl)-2,2′-bithiophenes (**2a**–**f**)

To a solution of bithiophene (39.00 g, 234 mmol) in dry THF (20 mL), *n*-BuLi (27 mL, 43 mmol) was added dropwise at 0 °C. The reaction mixture was allowed to warm to room temperature and stirred for an additional 20 min. Then, corresponding ω-bromoalkene (4.9 mL, 29 mmol) was added, and the mixture was refluxed overnight. After cooling to room temperature, water (40 mL) was added, and the organic phase was separated. The aqueous phase was extracted with dichloromethane (3 × 40 mL). The combined organic phases were dried over Na_2_SO_4,_ filtered through SiO_2_ pad and after the evaporation of the solvent, the product was purified by vacuum distillation. Further characterizations are reported in Supplementary Materials.

#### 2.2.3. General Procedure for 2-[ω-(2,2′-Bithiophen-5-yl)alkyl]dioxaphospholane 2-oxides (**3a**–**f**)

Corresponding 5-(alk-ω-enyl)-2,2′-bithiophene (500 mg, 1.8 mmol), 4,4,5,5-tetramethyl-1,3,2-dioxaphospholane 2-oxide (300 mg, 1.8 mmol.) and Pd(PPh_3_)_4_ (106 mg, 0.9 mmol, 0.05 equiv.) were dissolved in dry toluene (6 mL) in Schlenk flask. The mixture was heated at 110 °C for 12 h. The solvent was removed under vacuum, and the crude product was purified by chromatography on silica gel with hexanes and ethyl acetate (0→100% EtOAc) as eluents. Further characterizations are reported in Supplementary Materials.

#### 2.2.4. General Synthesis of ω-(2,2′-Bithiophen-5-yl)alkylphosphonic Acids **TTCnP**

Corresponding 2-[ω-(2,2′-bithiophen-5-yl)alkyl]-4,4,5,5-tetramethyldioxaphospho-lane 2-oxide (500 mg, 1.1 mmol) was dissolved in dry dichloromethane (5 mL) and BrSiMe_3_ (450 mg, 2.9 mmol, 2.5 equiv.) was added dropwise at 0 °C. The reaction mixture was stirred at 40 °C for 3 h, then allowed to stir at room temperature overnight. The solvent was removed, and the remaining solid was dissolved in methanol, and the product was precipitated with pentane. The solid was filtered and washed with pentane. Further characterizations are reported in Supplementary Materials.

#### 2.2.5. General Synthesis of ω-(2,2′-Bithiophen-5-yl)alkylchlorodimethylsilanes **TTCnSi**

To corresponding 5-(alk-ω-enyl)-2,2′-bithiophene (5.43 mmol) in dry toluene (100 mL), chlorodimethylsilane (13.7 mL, 11.80 g, 108 mmol, 20 equiv.) and Karstedt’s catalyst (108 μL, 2% Pt in xylene, 0.16 mmol, 0.03 equiv.) were added at 32 °C. The reaction mixture was stirred at 35 °C overnight. The unreacted chlorodimethylsilane and solvent were removed by distillation, and the greenish residue was purified by bulb-to-bulb distillation under reduced pressure (203 °C/0.4 Torr). Further characterizations are reported in Supplementary Materials.

### 2.3. Device Fabrication

The organic field-effect (OFET) devices have been fabricated in top-contact geometry. Heavily doped silicon wafers (boron-doped, 1–10 Ω∙cm) were used as the substrates, where the thermally grown oxide (SiO_2_) of 110 nm has been used as a gate insulator layer with a capacitance per unit of area of 33 nF/cm^2^. Prior to the organic film deposition, the substrates were sonicated in isopropyl alcohol and pure water, consecutively, and then cleaned up by UV/ozone treatment. SAM materials were dissolved in methanol with a concentration of 0.5 wt.%. Silicon substrates were immersed in SAM solution for 48 h in an inert atmosphere (nitrogen). Organic semiconductors pentacene or α-sexithiophene (both from Merck) were deposited onto bare and SAM-modified substrates using SPECTROS 100 deposition system (Kurt J. Lesker, East Sussex, UK) by thermal evaporation from a Knudsen cell (Al_2_O_3_ crucible) in a vacuum better than 10^−4^ Pa. The deposition rate was fixed at 3 nm/min and monitored by a quartz crystal microbalance (Kurt J. Lesker, East Sussex, UK). Copper electrodes were subsequently deposited by thermal evaporation in a vacuum through the shadow mask. The channel length varied from 50 to 200 μm, whereas the channel width was 2 mm for all the fabricated devices. All output and transfer characteristics of fabricated OFET devices of the structure shown in Figure 1 were measured in an ambient atmosphere.

### 2.4. Characterization

Melting points were determined with a Melting Point M-656 Büchi (BÜCHI Labortechnik AG, Flawil, Switzerland) apparatus and were uncorrected. IR spectra were determined with a Agilent Cary 630 FTIR Spectrometer (Agilent Technologies, Santa Clara, CA, USA). NMR spectra were recorded on the Varian VNMRS (Agilent Technologies Inc., Palo Alto, CA, USA) (600 MHz for ^1^H, 151 MHz for ^13^C, and 243 MHz for ^31^P). Chemical shifts are reported in δ ppm referenced to an internal SiMe_4_ standard for ^1^H NMR, and all measurements were performed at 20 °C if not stated differently. Compounds were measured in chloroform-*d1* (CDCl_3_) or dimethyl sulfoxide-*d6* (DMSO) The following abbreviations are used: s = singlet, d = doublet, dd = doublet of doublets, t = triplet, q = quartet, m = multiplet. HRMS was determined with an Orbitrap Velos Pro (Thermo Fisher Scientific Inc., Waltham, MS, USA). The spectral characteristics of synthesized compounds are reported in Supplementary Materials.

The fabricated devices were characterized by standard steady-state current-voltage measurement using the 4155 C Semiconductor Parameter Analyzer (Agilent Technologies, CA, USA) to determine threshold voltages and effective mobilities.

Surface morphology study of organic films was performed with the Dimension Edge Atomic Force Microscope (AFM) system (Veeco Instruments, CA, USA) operating in the tapping mode. The AFM probe (model FESPA-V2 from Bruker, MA, USA) with a soft cantilever (nominal spring constant, *k* = 2.8 N/m) was used to measure the film surface. The nominal tip radius was8 nm. The AFM images have been captured over the area of 1.2 × 1.2 μm^2^ and 2.4 × 2.4 μm^2^ for α-sexithiophene or pentacene films, respectively. The surface morphology images were taken from the middle of the OFET channels.

## 3. Results and Discussion

### 3.1. Synthesis of Surfactants

**Scheme 1:**
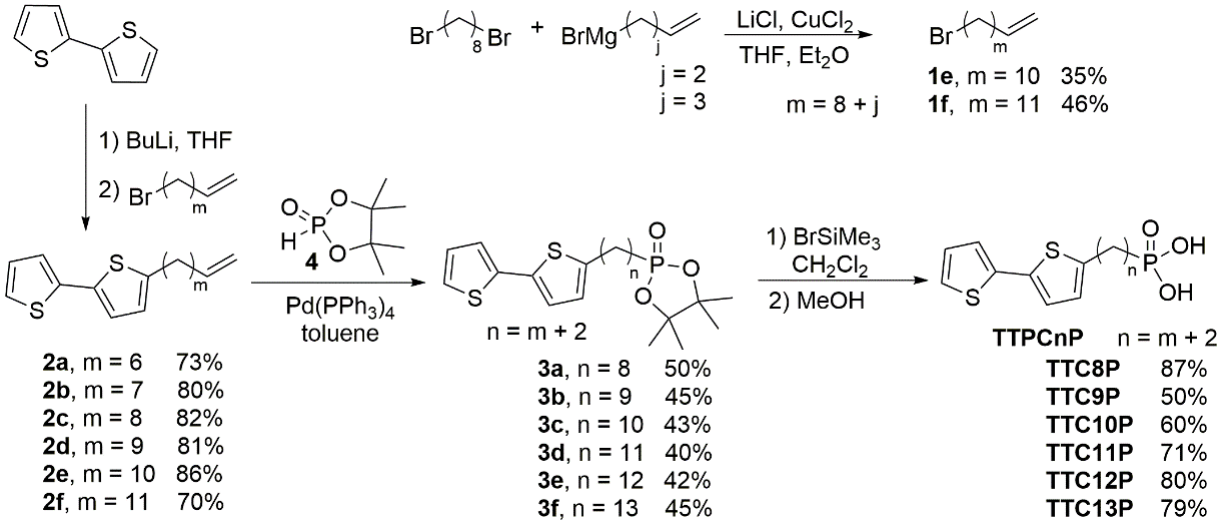
Synthesis of derivatives for surface modification **TTCnP** with phosphono anchoring group and various lengths of alkyl linker.

The synthetic strategy towards derivatives with phosphono anchoring group is outlined in Scheme 1. While shorter ω-bromoalkenes were commercially available, longer bromoalkenes **1e** and **1f** were synthesized by previously described copper-catalyzed cross-coupling from 1,8-dibromooctane and corresponding Grignard reagent [26,27]. The formation of disubstituted products causes a lower yield of these derivatives. However, these by-products can be easily separated by column chromatography. The bithienyl alkenes **2a**–**f** were obtained by the reaction of in-situ prepared 2-bithienyllithium with ω-bromoalkenes in good yields from 70% to 86%. Excess bithiophene in the reaction mixture was distilled off, and the residue was further purified by column chromatography. The synthesis of undecenylbithiophene **2f** has been previously described by Muzafarov et al. [28]. The monoalkylated bithiophenes **2a**–**f** were converted to dioxaphospholane oxides **3a**–**f** by Tanaka-Pd catalyzed hydrophosphorylation [29] using pinacol phosphonate (**4**). Alkylphosphonic acids **TTCnP** (*n* = 8–13) were obtained by treating corresponding dioxaphospholane oxides **3a**–**f** with BrSiMe_3_ [30,31] followed by anhydrous methanol [32] in good yields. All derivatives were characterized by ^1^H, ^13^C, ^31^P NMR, HRMS and FTIR. In some cases, 2D NMR was recorded to assign split carbon signals due to ^13^C–^31^P coupling.

**Scheme 2:**
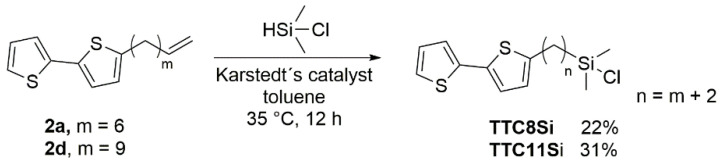
Synthesis of derivatives for surface modification **TTCnSi** with chlorodimethylsilyl anchoring group.

**Figure 3:**
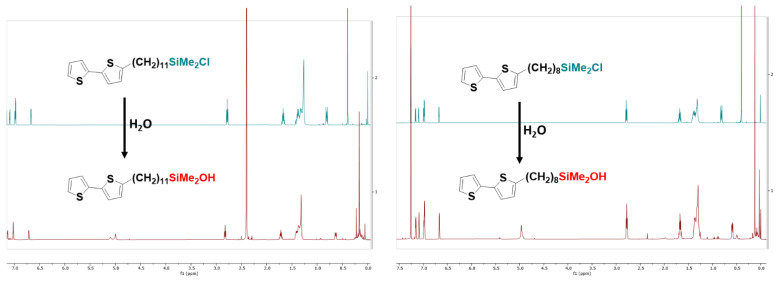
Changes observed in ^1^H NMR spectrum of chlorodimethylsilane derivatives **TTCnSi** by hydrolysis.

The chlorosilane derivatives **TTCnSi** (*n* = 8, 11) were obtained by a hydrosilylation reaction of bithienyl alkenes with chlorodimethylsilane in the presence of Karstedt’s catalyst (Scheme 2). Despite high conversion in these reactions, yields of the corresponding products **TTCnSi** were relatively low due to partial decomposition in vacuum distillation. We verified the presence of chlorine atom in the silane group by controlled hydrolysis confirmed by ^1^H NMR. After measurement of ^1^H NMR spectrum of the products **TTCnSi**, water was added to the NMR tube, and the ^1^H NMR spectrum was remeasured. Hydrolysis caused the change in shifts of silane methyl groups from 0.47 for **RSiMe_2_Cl** to 0.14 ppm for **RSiMe_2_OH** (Figure 3).

### 3.2. OFET Performance

**Figure 4:**
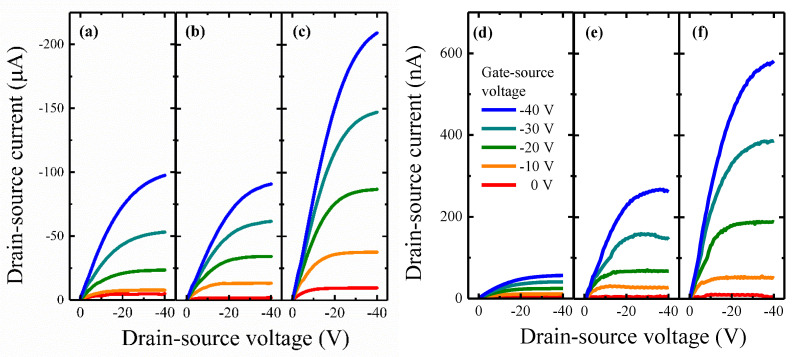
Output characteristics of pentacene OFET devices (channel length *L* of 50 μm) with (**a**–**c**) pentacene or (**d**–**f**) α-sexithiophene layer deposited on (**a**,**d**) bare SiO_2_ surface or SAM-modified surface with (**b**,**e**) **TTC11Si** and (**c**,**f**) **TTC11P**.

Pentacene and α-sexithiophene have been chosen as representative of typical and widely used organic semiconductors. Typical output characteristics of pentacene OFET devices with organic semiconductor layer deposited on bare SiO_2_ surface or SAM-modified surface are depicted in Figure 4. All fabricated devices exhibited standard transistor behavior. The surface modification does not influence the device performance significantly for the SAM with **TTC11Si** (chlorodimethylsilyl anchoring group), whereas the SAM with **TTC11P** (phosphono anchoring group) increases the output currents.

**Figure 5:**
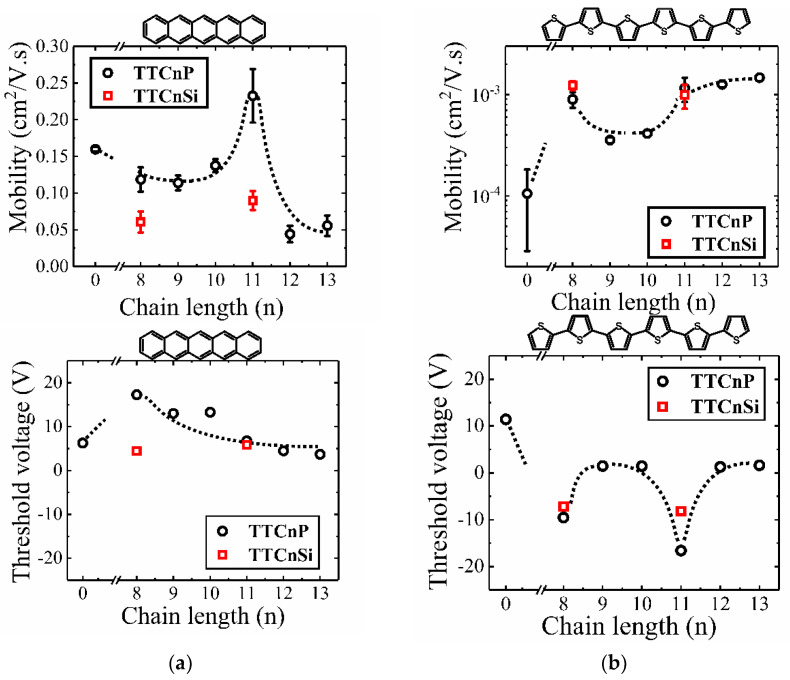
Effective mobilities and threshold voltages for (**a**) pentacene or (**b**) α-sexithiophene OFETs using bare and SAM-modified substrates.

The transfer characteristics have been used to evaluate the threshold voltage and the saturated effective mobility for all devices, as depicted in Figure 5. Interestingly, for pentacene-based devices, the effective mobility is slightly reduced with increasing alkyl chain length of phosphono-based surfactants **TTCnP** and significantly rises for an alkyl chain length of *n* = 11. Surprisingly, the SAM with **TTCnSi** (SiMe_2_Cl anchoring group) does not follow this tendency, and the effective mobility is suppressed. The anchoring groups are greatly affecting the packing density and molecular orientation. As a result, the bithienyl terminal group and pentacene have different interactions due to change mutual order. It should be noted here that since the organic semiconductors exhibit a certain distribution of material parameters, the effective mobility has been evaluated from a group of devices and the standard deviation was estimated (plotted as an error bar in Figure 5). The other way to verify the credibility of claimed effective mobilities is the evaluation of the reliability factor *r* [4]. The pentacene-based devices exhibit the reliability factor r of 92% ± 12%, whereas the α-sexithiophene-based devices had reliability factor on the level of 86% ± 12%. The trend of effective mobility is not accompanied by the threshold voltage that is almost conserved. The conservation of the threshold voltage indicates that the SAM modification does not cause additional trapping states on the organic semiconductor/gate insulator interface. On the other hand, the α-sexithiophene OFET devices exhibit a significant increase in effective mobility for all investigated SAM modifications of gate insulator surface. The threshold voltage is greatly shifted from a positive voltage to a zero value or a negative one. Observed shift points out the modification of the internal electric field due to charge trapping and/or interfacial dipole. Here, the SAM with silyl surfactants **TTCnSi** reaches almost identical results as the SAM with phosphono-based **TTCnP**.

### 3.3. Surface Morphology

**Figure 6:**
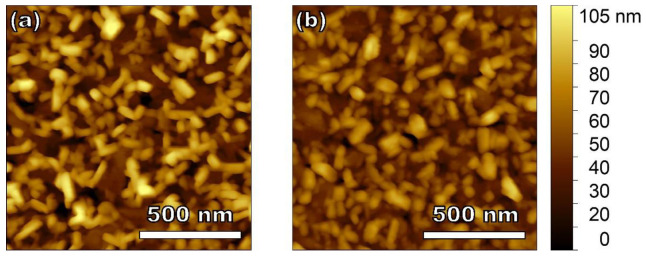
Surface morphology of α-sexithiophene layer deposited on (**a**) bare SiO_2_ or (**b**) **TTC11P** modified surface.

The thin-film morphology of the α-sexithiophene or pentacene film deposited on bare SiO_2_ and modified by **TTC11P** were analyzed by the Atomic Force Microscopy (AFM). The AFM images were taken from the middle of the transistor channel for both vacuum-deposited thin films onto bare and SAM treated surfaces. Figure 6 illustrates the α-sexithiophene films. The organic semiconductor forms elongated crystals with an average crystal length of 125 ± 29 nm on bare SiO_2_, whereas the length reaches 92 ± 26 nm on SAM treated surface. It should be noted here that according to the quartz crystal microbalance (QCM) measurement, the average film thickness of the deposited film was 100 nm and the AFM morphology revealed a similar height distribution range. In other words, the α-sexithiophene film is most probably not fully covering the substrate surface, and the charge transfer is due to the percolation mechanism.

**Figure 7:**
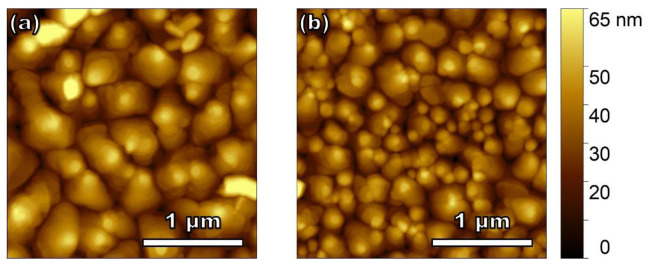
Surface morphology of pentacene layer deposited on (**a**) bare SiO_2_ or (**b**) **TTC11P** modified surface.

**Figure 8:**
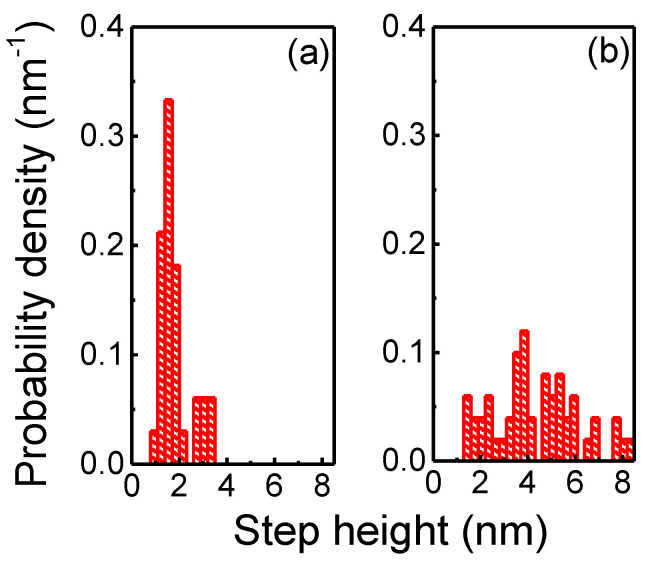
Probability density distribution of pentacene layer step heights for films deposited on (**a**) bare SiO_2_ or (**b**) **TTC11P** modified surface.

Figure 7 depicts the surface morphology of pentacene films. Pentacene displays polymorphism in its crystalline structure with two different phases [33,34,35]. Pentacene molecules onto the SiO_2_ surface form the “thin-film phase”, where the orthorhombic structure occurs. On the other hand, the “bulk phase” observed for thick film refers to the triclinic structure. The thin-film phase represents the layer-by-layer growth of pentacene films on a substrate surface. Hence, the incomplete layers, the terraces, have single-molecule thickness and are characterized by the inter-planar spacing *d*_001_ = 1.54 nm [33]. The pentacene films deposited on bare SiO_2_, shown in Figure 7a, exhibit terraces separated by steps of 1.5 ± 0.5 nm, and the presence of double layers is almost negligible, as shown in Figure 8a. Interestingly, the pentacene films deposited on **TTC11P** treated surface have obviously smaller grains with not well-defined terraces. Note that even though pentacene grains on SAM treated surfaces are usually smaller than those on SiO_2_, pentacene deposited on SAM has a lower defect density than pentacene on bare SiO_2_ [36,37]. The absence of observable flat terraces points out the presence of other molecular orientations. Once the bulk phase nucleates onto the thin-film phase, the coexistence of different structural phases causes smoothing of incrementation steps [35,38]. As a result, the SAM treatment causes an earlier transition from the thin-film phase to the bulk phase of pentacene films. It improves the effective mobility by a greatly larger contribution than it is mobility suppression due to the higher density of grain boundaries

## 4. Conclusions

In summary, we synthesized new surfactants with phosphono or chlorodimethylsilyl anchoring groups and various alkyl chain lengths by up to three-step synthesis. In the case of pentacene-based OFET, **TTC11P** surfactant showed a significant increase in effective mobility. The effective mobility of α-sexithiophene-based transistor increased in all cases of surface treatment with our surfactants. Investigation of the surface morphology showed that surface treatment with **TTC11P** causes the formation of smaller grains of pentacene with lower defect density and therefore results in higher mobility compared to OFET device with bare SiO_2_. Application of our organic semiconductors with bithienyl surfactants in OFET is under research.

## Data Availability

Data is contained within this article or its Supplementary Materials.

## Supplementary Materials

The following are available online at https://www.mdpi.com/article/10.3390/ma14216345/s1, Synthetic procedures and characterizations of prepared compounds. Figure S1–S59: ^1^H, ^13^C and ^31^P NMR spectra.
