# Supplementary material for: Synthesis and Effect of the Structure of Bithienyl-Terminated Surfactants for Dielectric Layer Modification in Organic Transistor
Source: Materials (Basel). 2021 Oct 23;14(21):6345. doi: 10.3390/ma14216345 (PMC8585473; doi:10.3390/ma14216345)
Supplement: Supplementary file 1 [file materials-14-06345-s001.zip › materials-1421057-supplementary.pdf]

Supplementary materials

Synthesis and effect of the structure of bithienyl-terminated surfactants for dielectric layer modification in organic transistor

Lucia Feriancová[1], Iveta Kmentová[1], Michal Mičjan[2], Milan Pavúk[3], Martin Weis [2]*, and Martin Putala[1]*

[1] Department of Organic Chemistry, Faculty of Natural Sciences, Comenius University in Bratislava, Ilkovičova 6, 842 15 Bratislava, Slovak Republic;

[2] Institute of Electronics and Photonics, Slovak University of Technology, Ilkovičova 3, 812 19 Bratislava, Slovak Republic;

[3] Institute of Nuclear and Physical Engineering, Slovak University of Technology, Ilkovičova 3, 812 19 Bratislava, Slovak Republic;

* Correspondence: martin.putala@uniba.sk, Tel.: +421 2 90149323 (M. P.); martin.weis@stuba.sk, Tel. +421 910942310 (M.W.)

Table of content

Synthetic procedures 1

^{1}H, ^{13}C and ^{31}P NMR spectra 9

Synthetic procedures

12-Bromododec-1-ene (1e)

To combined solutions of LiCl (254 mg, 6 mmol) and $CuCl_2$ (403 mg, 3 mmol) in dry THF, which was stirred overnight, 1,8-dibromooctane (4.08 g, 15 mmol) was added in one portion. The reaction mixture was cooled to 0 °C and but-3-en-1-ylmagnesium bromide (2.43 g, 18 mmol, 10 ml of 2M solution in Et_2O) was added through a cannula. After addition, the mixture was allowed to stir overnight at room temperature. Then, 1 M HCl was added and the mixture was stirred for another 30 min. The aqueous layer was extracted with diethyl ether. Combined organic layers were washed with brine, dried over Na_2SO_4, and the solvent was evaporated. The crude mixture was separated by flash chromatography (SiO_2, hexanes). The product was obtained as a colourless oil (1.30 g, 35 % yield). **^{1}H NMR** (600 MHz, $CDCl_3$): δ 5.81 (ddt, J = 16.9, 10.2, 6.7 Hz, 1H), 4.99 (dd, J = 17.1, 1.4 Hz, 1H), 4.93 (d, J = 10.1 Hz, 1H), 3.40 (t, J = 6.9 Hz, 2H), 2.04 (dd, J = 14.3, 7.0 Hz, 2H), 1.89–1.81 (m, 2H), 1.47–1.22 (m, 14H) ppm. **^{13}C NMR** (75 MHz, $CDCl_3$): δ 139.2, 114.1, 34.1, 33.8, 32.8, 29.5, 29.4, 29.4, 29.1, 28.9, 28.7, 28.2 ppm. **FTIR** (neat): ν 2923, 2852, 1640, 1460, 908 cm^{-1}. **HRMS** (pESI) m/z: $[M + Na]^+$ calcd for $C_{12}H_{23}BrNa$ 269.0883, found 269.0875.

13-Bromotridec-1-ene (1f)

Same procedure was used as for 12-bromododec-1-ene. Pent-4-en-1-ylmagnesium bromide was used (13.4 g, 90 mmol, 45 ml of 2M solution in Et_2O). The crude mixture was separated by flash chromatography (SiO_2, hexanes). The product was obtained as colourless oil (3.60 g, 46 % yield). **^{1}H NMR** (600 MHz, $CDCl_3$): δ 5.81 (ddt, *J* = 16.9, 10.2, 6.7 Hz, 1H), 4.99 (ddd, *J* = 17.1, 3.6, 1.6 Hz, 1H), 4.95 – 4.89 (m, 1H), 3.40 (t, *J* = 6.9 Hz, 2H), 2.04 (dd, *J* = 14.6, 6.9 Hz, 2H), 1.90–1.81 (m, 2H), 1.46–1.33 (m, 4H), 1.27 (s, 12H) ppm. **^{13}C NMR** (151 MHz, $CDCl_3$): δ 139.2, 114.1, 34.01 33.8, 32.8, 29.5, 29.5, 29.5, 29.4, 29.1, 28.9, 28.8, 28.2 ppm. **FTIR** (neat): *ν* 2922, 2852, 1640, 1463, 908 cm^{-1}. **HRMS** (pESI) *m/z*: $[M + Na]^+$ calcd for $C_{13}H_{25}BrNa$ 283.1039, found 283.1032.

5-(Oct-7-en-1-yl)-2,2'-bithiophene (2a)

To a solution of bithiophene (39.00 g, 234 mmol) in dry THF (20 mL), *n*-BuLi (27 mL, 43 mmol) was added dropwise at 0°C. The reaction mixture was allowed to warm to room temperature and stirred for additional 20 min. Then, 8-bromooct-1-ene (4.9 mL, 29 mmol) was added and the mixture was refluxed overnight. After cooling to room temperature, water (40 mL) was added and the organic phase was separated. The aqueous phase was extracted with dichloromethane (3 x 40 mL). The combined organic phases were dried over Na_2SO_4, filtered through Si_2O pad and after the evaporation of the solvent, the product was purified by vacuum distillation. The product was obtained as light-yellow liquid (5.94 g, 73 %). **^{1}H NMR** (300 MHz, $CDCl_3$): δ 7.15 (dd, *J* = 5.1, 1.1 Hz, 1H), 7.09 (dd, *J* = 3.6, 1.1 Hz, 1H), 6.98 (dd, *J* = 4.8, 3.6 Hz, 2H), 6.67 (d, *J* = 3.5 Hz, 1H), 5.81 (ddt, *J* = 16.9, 10.2, 6.7 Hz, 1H), 5.06–4.89 (m, 2H), 2.78 (t, *J* = 7.5 Hz, 2H), 2.04 (dd, *J* = 13.2, 6.2 Hz, 2H), 1.68 (dt, *J* = 14.9, 7.6 Hz, 2H), 1.45–1.31 (m, 6H) ppm. **^{13}C NMR** (151 MHz, $CDCl_3$): δ 145.3, 139.1, 138.0, 134.8, 127.7, 124.7, 123.7, 123.4, 123.0, 114.3, 33.8, 31.6, 30.1, 28.9, 28.9, 28.8 ppm. **FTIR** (neat): *ν* 3069, 2924, 2852, 1638, 792, 686 cm^{-1}. **HRMS** (pESI) *m/z*: $[M + H]^+$ calcd for $C_{16}H_{20}S_2H$ 277.1086, found 277.1079.

5-(Non-8-en-1-yl)-2,2'-bithiophene (2b)

Same procedure was used as for **2a** using 16.21 g of bithiophene. After distillation, the remaining dark distillation residue containing mostly product was further purified by flash chromatography (SiO_2, hexanes). The product was obtained as a colourless liquid (2.27 g, 80%). **^{1}H NMR** (600 MHz, $CDCl_3$): δ 7.16 (dd, *J* = 5.1, 1.1 Hz, 1H), 7.10 (dd, *J* = 3.6, 1.1 Hz, 1H), 7.01–6.97 (m, 2H), 6.68 (d, *J* = 3.5 Hz, 1H), 5.82 (ddt, *J* = 16.9, 10.2, 6.7 Hz, 1H), 5.00 (dd, *J* = 17.1, 1.6 Hz, 1H), 4.94 (dd, *J* = 10.2, 1.0 Hz, 1H), 2.79 (t, *J* = 7.6 Hz, 2H), 2.05 (dd, *J* = 14.2, 7.1 Hz, 2H), 1.68 (dt, *J* = 15.3, 7.5 Hz, 2H), 1.43–1.29 (m, 8H) ppm. **^{13}C NMR** (151 MHz, $CDCl_3$): δ 145.3, 139.1, 137.9, 134.7, 127.6, 124.8, 123.7, 123.4, 123.0, 114.2, 33.8, 31.6, 30.1, 29.2, 29.0 (2C), 28.9 ppm. **FTIR** (neat): *ν* 3069, 2922, 2851, 1638, 792, 686 cm^{-1}. **HRMS** (pESI) *m/z*: $[M + H]^+$ calcd for $C_{17}H_{22}S_2H$ 291.1241, found 291.1236.

5-(Dec-9-en-1-yl)-2,2'-bithiophene (2c)

Same procedure was used as for **2a** using 15.17 g of bithiophene. The excess amount of bithiophene was distilled out under reduced pressure and dark distillation residue was purified by flash chromatography (SiO_2, hexanes). The product was obtained as a colourless liquid (2.28 g, 82%). **^{1}H NMR** (600 MHz, $CDCl_3$): δ 7.16 (dd, *J* = 5.1, 1.1 Hz, 1H), 7.10 (dd, *J* = 3.6, 1.1

Hz, 1H), 7.02–6.96 (m, 2H), 6.68 (d, *J* = 3.5 Hz, 1H), 5.82 (ddt, *J* = 16.9, 10.2, 6.7 Hz, 1H), 5.00 (dd, *J* = 17.1, 1.7 Hz, 1H), 4.94 (dd, *J* = 10.2, 0.9 Hz, 1H), 2.79 (t, *J* = 7.6 Hz, 2H), 2.09–2.01 (m, 2H), 1.72–1.62 (m, 2H), 1.42–1.26 (m, 10H) ppm.**^{13}C NMR** (151 MHz, $CDCl_3$): δ 145.3, 139.2, 138.0, 134.7, 127.6, 124.7, 123.7, 123.4, 123.0, 114.1, 33.8, 31.6, 30.1, 29.4, 29.3, 29.1, 29.0, 28.9 ppm. **FTIR** (neat): *ν* 3069, 2922, 2851, 1638, 792, 686 cm^{-1}. **HRMS** (pESI) *m/z*: $[M + H]^+$ calcd for $C_{18}H_{24}S_2H$ 305.1397, found 305.1392.

5-(Undec-10-en-1-yl)-2,2'-bithiophene (2d)

Same procedure was used as for **2a**, using 45.65 g of bithiophene. The product was obtained as a light green oil (10.36 g, 81%). **^{1}H NMR** (300 MHz, $CDCl_3$): δ 7.16 (dd, *J* = 5.1, 1.0 Hz, 1H), 7.10 (dd, *J* = 3.6, 1.1 Hz, 1H), 7.03–6.94 (m, 2H), 6.67 (d, *J* = 3.5 Hz, 1H), 5.91–5.72 (m, 1H), 5.14–4.83 (m, 2H), 2.79 (t, *J* = 7.5 Hz, 2H), 2.04 (dd, *J* = 14.2, 6.8 Hz, 2H), 1.68 (dt, *J* = 15.0, 7.6 Hz, 2H), 1.43–1.23 (m, 12H) ppm. **^{13}C NMR** (151 MHz, $CDCl_3$): δ 145.3, 139.2, 138.0, 134.7, 127.6, 124.7, 123.7, 123.4, 123.0, 114.1, 33.8, 31.6, 30.1, 29.5, 29.5, 29.3, 29.1, 29.1, 28.9 ppm. **FTIR** (neat): *ν* 2922, 2851, 1458, 907, 792, 686 cm^{-1}. **HRMS** (pESI) *m/z*: $[M + H]^+$ calcd for $C_{19}H_{26}S_2H$ 319.1556, found 319.1549.

5-(Dodec-11-en-1-yl)-2,2'-bithiophene (2e)

Same procedure was used as for **2a** using 6.73 g of bithiophene. The excess amount of bithiophene was distilled out under reduced pressure and distillation residue was purified by flash chromatography (SiO_2, hexanes). The product was obtained as colourless oil (2.90 g, 86% yield). **^{1}H NMR** (600 MHz, $CDCl_3$): δ 7.15 (dd, *J* = 5.1, 1.0 Hz, 1H), 7.09 (dd, *J* = 3.5, 1.0 Hz, 1H), 7.00–6.96 (m, 2H), 6.67 (d, *J* = 3.5 Hz, 1H), 5.81 (ddt, *J* = 16.9, 10.2, 6.7 Hz, 1H), 5.03–4.97 (m, 1H), 4.95–4.90 (m, 1H), 2.78 (t, *J* = 7.6 Hz, 2H), 2.04 (dd, *J* = 14.5, 6.9 Hz, 2H), 1.67 (dt, *J* = 15.2, 7.5 Hz, 2H), 1.40–1.34 (m, 4H), 1.27 (s, 10H) ppm. **^{13}C NMR** (151 MHz, $CDCl_3$): δ 145.3, 139.2, 138.0, 134.7, 127.6, 124.7, 123.7, 123.4, 123.0, 114.1, 33.8, 31.6, 30.1, 29.6, 29.5, 29.5, 29.4, 29.1, 29.1, 28.9 ppm. **FTIR** (neat): *ν* 2921, 2851, 1638, 793, 687 cm^{-1}. **HRMS** (pESI) *m/z*: $[M + H]^+$ calcd. for $C_{20}H_{29}S_2$ 333.1712, found 333.1705.

5-(Tridec-12-en-1-yl)-2,2'-bithiophene (2f)

Same procedure was used as for **2a** using 12.73 g of bithiophene. The product was obtained as white solid (1.85 g, 70% yield). **M.p.** = 28.1-29.9 °C. **^{1}H NMR** (600 MHz, $CDCl_3$): δ 7.16 (dd, *J* = 5.1, 1.0 Hz, 1H), 7.09 (dd, *J* = 3.5, 0.9 Hz, 1H), 7.00–6.96 (m, 1H), 6.67 (d, *J* = 3.5 Hz, 1H), 5.81 (ddt, *J* = 16.9, 10.2, 6.7 Hz, 1H), 4.99 (dd, *J* = 17.1, 1.8 Hz, 1H), 4.95–4.90 (m, 1H), 2.78 (t, *J* = 7.6 Hz, 2H), 2.04 (dd, *J* = 14.5, 6.9 Hz, 2H), 1.72–1.63 (m, 2H), 1.37 (m, 4H), 1.29 (m, 12H) ppm. **^{13}C NMR** (151 MHz, $CDCl_3$): δ 145.4, 139.3, 138.0, 134.7, 127.6, 124.7, 123.7, 123.4, 122.9, 114.1, 33.8, 31.6, 30.1, 29.6, 29.6, 29.5, 29.5, 29.3, 29.1, 29.1, 28.9 ppm. **FTIR** (neat): *ν* 2913, 2847, 1467, 789, 693 cm^{-1}. **HRMS** (pESI) *m/z*: $[M + H]^+$ calcd for $C_{21}H_{30}S_2H$ 347.1869, found 347.1862.

[8-(2,2'-Bithiophen-5-yl)octyl]chlorodimethylsilane (TTC8Si)

To 5-(oct-7-en-1-yl)-2,2'-bithiophene (1.5 g, 5.43 mmol) in dry toluene (100 mL), chlorodimethylsilane (13.7 mL, 11.80 g, 108 mmol, 20 equiv.) and Carsted catalyst (108 μL, 2% Pt in xylene, 0.16 mmol, 0.03 equiv.) were added at 32°C. The reaction mixture was stirred at 35°C overnight. The unreacted chlorodimethylsilane and solvent were removed by

distillation, and the greenish residue was purified by bulb-to-bulb distillation under reduced pressure (203°C/ 0.4 Torr). The product was isolated as a colourless liquid (444 mg, 22%). ^{1}**H NMR** (600 MHz, $CDCl_3$): *δ* 7.16 (dd, *J* = 5.1, 1.1 Hz, 1H), 7.10 (dd, *J* = 3.6, 1.0 Hz, 1H), 7.00–6.97 (m, 2H), 6.68 (d, *J* = 3.5 Hz, 1H), 2.79 (t, *J* = 7.6 Hz, 2H), 1.68 (dt, *J* = 15.2, 7.5 Hz, 2H), 1.46–1.27 (m, 12H), 0.82 (dd, *J* = 9.5, 6.9 Hz, 2H), 0.43 – 0.38 (m, 6H) ppm. ^{13}**C NMR** (151 MHz, $CDCl_3$): *δ* 145.4, 138.0, 134.8, 127.7, 124.7, 123.7, 123.4, 123.0, 33.4, 31.6, 30.2, 29.3, 29.3, 29.2, 23.3, 18.4, 0.5, 0.4 ppm. **FTIR** (neat): *ν* 2917,0 2848, 1249, 1068, 835, 778, 683 cm^{-1}. **HRMS** does not ionize, measured by ESI (with addition of AcOH), APPI in MeOH and MeCN.

[11-(2,2'-Bithiophen-5-yl)undecyl]chlorodimethylsilane (TTC11Si)

Same procedure was used as for **TTC8Si** using 5-(undec-10-en-1-yl)-2,2'-bithiophene (638 mg, 2 mmol) ,40 mL of dry toluene, 5.1 mL of chlorodimethylsilane (40 equiv.) and 0.06 mmol of Carsted catalyst (0.03 equiv.). The product was isolated as a colourless liquid (245 mg, 31% yield). ^{1}**H NMR** (600 MHz, $CDCl_3$): δ 7.16 (d, *J* = 5.1 Hz, 1H), 7.09 (d, *J* = 3.5 Hz, 1H), 7.00–6.96 (m, 2H), 6.67 (d, *J* = 3.5 Hz, 1H), 2.78 (t, *J* = 7.6 Hz, 2H), 1.72–1.63 (m, 3H), 1.45–1.22 (m, 20H), 0.81 (dd, *J* = 9.5, 6.9 Hz, 2H), 0.41–0.39 (m, 6H) ppm. ^{13}**C NMR** (151 MHz, $CDCl_3$): δ 145.4, 138.0, 134.7, 127.6, 124.6, 123.7, 123.4, 122.9, 33.4, 31.6, 30.1, 29.7, 29.6, 29.6, 29.4, 29.4, 29.1, 23.3, 18.4, 0.4 (2C) ppm. **FTIR** (neat): *ν* 2915, 2847, 1465, 1243, 1063, 836, 784, 681 cm^{-1}. **HRMS** does not ionize, measured by ESI (with addition of AcOH), APPI in MeOH and MeCN.

2-[8-(2,2'-Bithiophen-5-yl)octyl]-4,4,5,5-tetramethyl-1,3,2-dioxaphospholane 2-oxide (3a)

5-(Oct-7-en-1-yl)-2,2'-bithiophene (500 mg, 1.8 mmol, 1 equiv.), 4,4,5,5-tetramethyl-1,3,2-dioxaphospholane 2-oxide (300 mg, 1.8 mmol, 1 equiv.) and $Pd(PPh_3)_4$ (106 mg, 0.9 mmol, 0.05 equiv.) were dissolved in dry toluene (6 mL) in Schlenk flask. The mixture was heated at 110 °C for 12 h. The solvent was removed under vacuum and crude product was purified by chromatography on silica gel with hexanes and EtOAc (0→100% EtOAc) as eluents. Product was isolated as a yellow solid (380 mg, 50% yield). **M.p.** = 48.1-52.0 °C. ^{1}**H NMR** (600 MHz, $CDCl_3$): δ 7.15 (dd, J = 5.1, 1.1 Hz, 1H), 7.09 (dd, J = 3.6, 1.1 Hz, 1H), 7.00–6.95 (m, 2H), 6.66 (d, J = 3.5 Hz, 1H), 2.77 (t, *J* = 7.5 Hz, 2H), 1.83 (m, 2H), 1.69 (m, 4H), 1.48 (s, 6H), 1.44–1.30 (m, 14H). (at 5.3 residue of dichloromethane) ppm ^{31}**P NMR** (243 MHz, $CDCl_3$): δ 44.29 ppm. ^{13}**C NMR** (151 MHz, $CDCl_3$): δ 145.2, 137.9, 134.7, 127.6, 124.7, 123.7, 123.4, 123.0, 87.7 (d, *J* =1.4 Hz, 2C 4°), 31.5, 30.6 (d, *J* = 15.6 Hz), 30.1, 29.0, 29.0, 28.9, 28.1 (d, $^{1}J_{PC}$ = 131.6 Hz, 1C 2^0), 24.7 (d, *J* = 3.8 Hz, 2C 1^0), 24.1 (d, *J* = 5.3 Hz, 2C 1^0), 22.9 (d, *J* = 5.2 Hz, 1C 2^0) ppm. **FTIR** (neat): *ν* 2925, 1255, 959, 925 cm^{-1}. **HRMS** (pESI) *m/z*: $[M + H]^+$ calcd for $C_{22}H_{33}O_3PS_2H$ 441.1689, found 441.1682.

2-[9-(2,2'-Bithiophen-5-yl)nonyl]-4,4,5,5-tetramethyl-1,3,2-dioxaphospholane 2-oxide (3b)

The same procedure and equivalents of starting materials were used as for **3a** starting from 2 g (6.88 mmol) of **2b**. The product was isolated as white solid (1.40 g, 45%). **M. p.** = 68.7-69.8 °C. ^{1}**H NMR** (600 MHz, $CDCl_3$): δ 7.15 (dd, *J* = 5.1, 0.9 Hz, 1H), 7.09 (dd, *J* = 3.5, 0.9 Hz, 1H), 7.00–6.96 (m, 2H), 6.67 (d, *J* = 3.5 Hz, 1H), 2.78 (t, *J* = 7.6 Hz, 2H), 1.89–1.79 (m, 2H), 1.75–1.62 (m, 4H), 1.48 (s, 6H), 1.42–1.28 (m, 16H) ppm. ^{31}**P NMR** (243 MHz, $CDCl_3$): δ

44.31 ppm. **^{13}C NMR** (151 MHz, $CDCl_3$): δ 145.3, 137.9, 134.7, 127.6, 124.7, 123.7, 123.4, 123.0, 87.7 (d, $^2J_{PC}$ = 1.5 Hz, 2C (4^0)), 31.6, 30.6 (d, *J* = 15.6 Hz, 1C, (2^0)), 30.1, 29.3, 29.2, 29.0, 29.0, 28.1 (d, *J* = 131.6 Hz, 1C (2^0)), 24.7 (d, *J* = 3.7 Hz, 2C (1^0)), 24.1 (d, *J* = 5.3 Hz, 2C (1^0)), 22.9 (d, *J* = 5.2 Hz, 1C (2^0)) ppm. **FTIR** (neat): *ν* 2923, 2846, 1262, 1138, 922, 801, 698 cm^{-1}. **HRMS** (pESI) *m/z*: $[M + H]^+$ calcd for $C_{23}H_{35}O_3PS_2H$ 455.1843, found 455.1838.

2-[10-(2,2'-Bithiophen-5-yl)decyl]-4,4,5,5-tetramethyl-1,3,2-dioxaphospholane 2-oxide (3c)

The same procedure and equivalents of starting materials were used as for **3a** starting from 1.8 g (5.2 mmol) of **2c**. The product was isolated as white solid (1.20 g, 43%). **M. p.** = 60.2-62.3 °C. **^{1}H NMR** (600 MHz, $CDCl_3$): δ 7.16 (dd, *J* = 5.1, 1.1 Hz, 1H), 7.09 (dd, *J* = 3.6, 1.0 Hz, 1H), 7.01–6.95 (m, 2H), 6.67 (d, *J* = 3.5 Hz, 1H), 2.78 (t, *J* = 7.6 Hz, 2H), 1.89–1.77 (m, 2H), 1.75–1.64 (m, 4H), 1.49 (s, 6H), 1.41–1.24 (m, 18H) ppm. **^{13}C NMR** (151 MHz, $CDCl_3$): δ 145.3, 137.9, 134.7, 127.6, 124.7, 123.7, 123.4, 122.9, 87.7 (d, *J* = 1.4 Hz, 2C (4^0)), 31.6, 30.6 (d, *J* = 15.7 Hz, 1C 2^0), 30.1, 29.4, 29.3, 29.3, 29.1, 29.0, 28.1 (d, *J* = 131.6 Hz, 1C 2^0), 24.7 (d, *J* = 3.7 Hz, 2C 1^0), 24.1 (d, *J* = 5.3 Hz, 2C (1^0)), 22.9 (d, *J* = 5.2 Hz, 1C 2^0) ppm. **^{31}P NMR** (243 MHz, $CDCl_3$): δ 44.34 ppm. **FTIR** (neat): *ν* 2918, 2849, 1466, 1262, 1133, 960, 929, 719 cm^{-1}. **HRMS** (pESI) *m/z*: $[M + Na]^+$ calcd for $C_{24}H_{37}O_3PS_2Na$ 491.1820, found 491.1814.

2-[11-(2,2'-Bithiophen-5-yl)undecyl]-4,4,5,5-tetramethyl-1,3,2-dioxaphospholane 2-oxide (3d)

The same procedure and equivalents of starting materials were used as for **3a** starting from 300 mg (0.9 mmol) of **2d**. The product was isolated as white solid (180 mg, 40% yield). **M.p.** = 54.3-56.2 °C. **^{1}H NMR** (300 MHz, $CDCl_3$): δ 7.16 (dd, *J* = 5.1, 1.1 Hz, 1H), 7.09 (dd, *J* = 3.6, 1.1 Hz, 1H), 6.98 (m, 2H), 6.67 (d, *J* = 3.5 Hz, 1H), 2.78 (t, *J* = 7.5 Hz, 2H), 1.93–1.60 (m, 8H), 1.49 (s, 6H), 1.34 (m, 18H) ppm. **^{13}C NMR** (151 MHz, $CDCl_3$): δ 145.3, 137.9, 134.7, 127.6, 124.7, 123.7, 123.4, 122.9, 87.7 (d, *J* = 1.5 Hz, 2C 4˚), 31.6, 30.6 (d, *J* = 15.7 Hz, 1C 2^0), 30.1, 29.5, 29.5, 29.3, 29.1, 29.0, 28.1 (d, *J* = 131.5 Hz, 1C 2^0), 24.7 (d, *J* = 3.8 Hz, 2C 1^0), 24.1 (d, *J* = 5.3 Hz, 2C 1^0), 22.9 (d, *J* = 5.2 Hz, 1C 2^0) ppm. **^{31}P NMR** (243 MHz, $CDCl_3$): δ 44.35 ppm. **FTIR** (neat): *ν* 2919, 2846, 1257, 959, 926 cm^{-1}. **HRMS** (pESI) *m/z*: $[M + H]^+$ calcd for $C_{25}H_{39}O_3PS_2$ H 483.2158, found 483.2151.

2-[12-(2,2'-Bithiophen-5-yl)dodecyl]-4,4,5,5-tetramethyl-1,3,2-dioxaphospholane 2-oxide (3e)

The same procedure and equivalents of starting materials were used as for **3a** starting from 2 g (6.01 mmol) of **2e**. Product was isolated as a white solid (1.26 g, 42% yield). **M.p.** = 57.4-59.8 °C. **^{1}H NMR** (600 MHz, $CDCl_3$): δ 7.15 (dd, *J* = 5.1, 1.1 Hz, 1H), 7.08 (dd, *J* = 3.6, 1.1 Hz, 1H), 7.00–6.95 (m, 2H), 6.66 (d, *J* = 3.5 Hz, 1H), 2.77 (t, *J* = 7.6 Hz, 2H), 1.87–1.78 (m, 2H), 1.75–1.62 (m, 4H), 1.48 (s, 6H), 1.41–1.22 (m, 23H) ppm. **^{31}P NMR** (243 MHz, $CDCl_3$): δ 44.40 ppm. **^{13}C NMR** (151 MHz, $CDCl_3$): δ 145.3, 137.9, 134.7, 127.6, 124.7, 123.7, 123.3, 122.9, 87.7 (d, *J* = 1.5 Hz, 2C 4˚), 31.6, 30.6 (d, *J* = 15.7 Hz, 1C 2^0), 30.1, 29.6, 29.5, 29.5, 29.3, 29.1, 29.1, 28.1 (d, *J* = 131.6 Hz, 1C 2^0), 24.7 (d, *J* = 3.7 Hz, 2C 1^0), 24.1 (d, *J* = 5.3 Hz, 2C 1^0), 22.9 (d, *J* = 5.2 Hz, 1C 2^0) ppm. **FTIR** (neat): *ν* 2919, 2847, 1256, 1134, 957, 928 cm^{-1}. **HRMS** (pESI) *m/z*: $[M + H]^+$ calcd for $C_{26}H_{41}O_3PS_2H$ 497.2315, found 497.2308.

2-[13-(2,2'-Bithiophen-5-yl)tridecyl]-4,4,5,5-tetramethyl-1,3,2-dioxaphospholane 2-oxide (3f)

The same procedure and equivalents of starting materials were used as for **3a** starting from 1.4 g (4.04 mmol) of **2f**. Product was isolated as a white solid (0.90 g, 45% yield). **M. p.** = 67.8-70.0 °C. **^{1}H NMR** (600 MHz, $CDCl_3$): δ 7.15 (dd, *J* = 5.1, 1.1 Hz, 1H), 7.08 (dd, *J* = 3.6, 1.1 Hz, 1H), 7.00–6.95 (m, 2H), 6.66 (d, *J* = 3.5 Hz, 1H), 2.77 (t, *J* = 7.6 Hz, 2H), 1.89–1.79 (m, 2H), 1.68 (ddd, *J* = 22.7, 11.5, 6.4 Hz, 4H), 1.48 (s, 6H), 1.41–1.23 (m, 24H) ppm. **^{31}P NMR** (243 MHz, $CDCl_3$) δ 44.41 ppm. **^{13}C NMR** (151 MHz, $CDCl_3$): δ 145.3, 137.9, 134.7, 127.6, 124.7, 123.7, 123.3, 122.9, 87.7 (d, *J* = 1.5 Hz, 2C 4°), 31.6, 30.6 (d, *J* = 15.7 Hz, 1C 2^0), 30.1, 29.6, 29.6, 29.6, 29.5, 29.3, 29.1, 29.1, 28.1 (d, *J* = 131.5 Hz, 1C 2^0), 24.7 (d, *J* = 3.8 Hz, 2C 1^0), 24.1 (d, *J* = 5.3 Hz, 2C 1^0), 22.9 (d, *J* = 5.2 Hz, 1C 2^0) ppm. **FTIR** (neat): *v* 2917, 2848, 1259, 964, 930, 713, 689 cm^{-1}. **HRMS** (pESI) *m/z*: $[M + H]^+$ calcd for $C_{27}H_{43}O_3PS_2H$ 511.2471, found 511.2464.

8-(2,2'-Bithiophen-5-yl)octylphosphonic acid (TTC8P)

2-[8-(2,2'-Bithiophen-5-yl)octyl]-4,4,5,5-tetramethyl-1,3,2-dioxaphospholane 2-oxide (500 mg, 1.1 mmol) was dissolved in dry dichloromethane (5 mL) and $BrSiMe_3$ (450 mg, 2.9 mmol, 2.5 equiv.) was added dropwise at 0 °C. The reaction mixture was stirred at 40 °C for 3 h, then allowed to stir at room temperature overnight. The solvent was removed and the remaining solid was dissolved in methanol and product was precipitated with pentane. The white solid was filtered and washed with anhydrous pentane. After drying in vacuum, product was isolated as a white powder (366 mg, 87% yield). **M. p.** = 113.8-117.3 °C. **^{1}H NMR** (600 MHz, DMSO): δ 7.42 (dd, *J* = 5.1, 0.8 Hz, 1H), 7.18 (d, *J* = 2.7 Hz, 1H), 7.06 (d, *J* = 3.5 Hz, 1H), 7.03 (dd, *J* = 5.0, 3.6 Hz, 1H), 6.77 (d, *J* = 3.5 Hz, 1H), 3.46 (br.s, 2x OH included in residual water in DMSO), 2.75 (t, *J* = 7.5 Hz, 2H), 1.63–1.55 (m, 2H), 1.49–1.39 (m, 4H), 1.34–1.19 (m, 8H) ppm. **^{31}P NMR** (243 MHz, DMSO): δ 26.46 ppm. **^{13}C NMR** (151 MHz, DMSO): δ 145.1, 137.3, 134.4, 128.6, 125.9, 125.2, 124.0, 123.7, 31.5, 30.5, 30.4, 29.7, 29.1, 29.0, 28.8, 28.0 (d, $^1J_{PC}$ = 136.2 Hz, 1C $-CH_2-P(O)$) 23.2 (d, $^2J_{PC}$ = 4.6 Hz, CH_2) ppm. **FTIR** (neat): *v* 2921, 2847, 1463, 938, 789, 696 cm^{-1}. **HRMS** (pESI) *m/z*: $[M + H]^+$ calcd for $C_{16}H_{23}O_3PS_2H$ 359.0906; found 359.0899.

9-(2,2'-Bithiophen-5-yl)nonylphosphonic acid (TTC9P)

The same procedure and equivalents of starting materials were used as for **TTC8P** starting from 1.1g (2.42 mmol) of **3b**. Few drops of HCl (conc.) were added to methanol/pentane solution to improve precipitation. The product was isolated as a grey powder (0.42 g, 50%). **M. p.** = 123.4-126.4 °C. **^{1}H NMR** (600 MHz, DMSO): δ 7.44 (d, *J* = 5.1 Hz, 1H), 7.20 (d, *J* = 3.5 Hz, 1H), 7.08 (d, *J* = 3.5 Hz, 1H), 7.05 (dd, *J* = 5.0, 3.6 Hz, 1H), 6.79 (d, *J* = 3.5 Hz, 1H), 2.77 (t, *J* = 7.5 Hz, 2H), 1.65–1.57 (m, 2H), 1.51–1.41 (m, 4H), 1.28 (m, 12H) ppm. **^{31}P NMR** (243 MHz, DMSO): δ 27.24 ppm. **^{13}C NMR** (151 MHz, DMSO): δ 145.2, 137.2, 134.3, 128.8, 126.0, 125.0, 124.0, 123.7, 31.3, 30.3 (d, *J* = 16.4 Hz), 29.6, 29.0, 28.9, 28.9, 28.6, 27.5 (d, *J* = 135.2 Hz), 22.8 (d, *J* = 4.7 Hz) ppm. **FTIR** (neat): *v* 2920, 2846, 1463, 996, 951, 695 cm^{-1}. **HRMS** (pESI) *m/z*: $[M + H]^+$ calcd for $C_{17}H_{25}O_3PS_2H$ 373.1061, found 373.1055.

10-(2,2'-Bithiophen-5-yl)decylphosphonic acid (TTC10P)

The same procedure and equivalents of starting materials were used as for 8 **TTC8P** starting from 1 g (2.13 mmol) of **3c**. Few drops of HCl (conc.) were added to methanol/pentane solution to improve precipitation. The product was isolated as a white powder (0.46 g, 60%). **M. p.** = 116.8-119.3 °C.**^{1}H NMR** (600 MHz, DMSO): δ 7.44 (d, *J* = 4.4 Hz, 1H), 7.20 (d, *J* = 2.9 Hz, 1H), 7.08 (d, *J* = 3.5 Hz, 1H), 7.05 (dd, *J* = 5.0, 3.6 Hz, 1H), 6.78 (d, *J* = 3.5 Hz, 1H), 2.76 (t, *J* = 7.5 Hz, 2H), 1.68–1.57 (m, 2H), 1.53–1.40 (m, 4H), 1.36–1.21 (m, 14H) ppm. **^{31}P NMR** (243 MHz, DMSO): δ 27.32 ppm. **^{13}C NMR** (151 MHz, DMSO): δ 145.2, 137.2, 134.3, 128.8, 126.0, 125.0, 124.0, 123.7, 31.3, 30.3 (d, *J* = 16.2 Hz), 29.6, 29.2, 29.0, 28.9, 28.9, 28.5, 27.5 (d, *J* = 135.5 Hz), 22.8 (d, *J* = 4.7 Hz) ppm. **FTIR** (neat): *v* 2916, 2846, 1463, 1044, 1005, 956, 788, 691 cm^{-1}. **HRMS** (pESI) *m/z*: $[M + H]^+$ calcd for $C_{18}H_{27}O_3PS_2H$ 387.1217, found 387.1212.

11-(2,2'-Bithiophen-5-yl)undecylphosphonic acid (TTC11P)

The same procedure and equivalents of starting materials were used as for **TTC8P** starting from 0.4 g (0.829 mmol) of **3d**. The product was isolated as a grey powder (51 mg, 71% yield). **M. p.** = 110.2-113.1 °C. **^{1}H NMR** (600 MHz, DMSO): δ 7.38 (d, *J* = 4.9 Hz, 1H), 7.15 (d, *J* = 2.9 Hz, 1H), 7.06–6.99 (m, 2H), 6.74 (d, *J* = 2.9 Hz, 1H), 2.71 (t, *J* = 7.3 Hz, 2H), 1.59–1.51 (m, 2H), 1.44 (m, 4H), 1.22 (m, *J* = 40.4 Hz, 16H) ppm.**^{13}C NMR** (151 MHz, DMSO): δ 145.1, 137.3, 134.4, 128.7, 125.9, 125.1, 124.0, 123.7, 31.4, 30.5 (d, *J* = 16.2 Hz), 29.7, 29.4, 29.3, 29.3, 29.1, 29.1, 28.7, 27.9 (d, *J* = 135.9 Hz), 23.1 (d, *J* = 4.6 Hz) ppm. **^{31}P NMR** (243 MHz, DMSO): δ 26.69 ppm. **FTIR** (neat): *v* 2913, 2845, 1462, 1426, 1004, 951, 802, 689, 538 cm^{-1}. **HRMS** (pESI) *m/z*: $[M + H]^+$ calcd for $C_{19}H_{29}O_3PS_2H$ 401.1376, found 401.1371.

12-(2,2'-Bithiophen-5-yl)dodecylphosphonic acid (TTC12P)

The same procedure and equivalents of starting materials were used as for **TTC8P** starting from 1.00 g (2.01 mmol) of **3e**. Few drops of HCl (conc.) were added to methanol/pentane solution to improve precipitation. The product was isolated as a grey powder (660 mg, 80% yield). **M. p.** = 98.1-101.8 °C. **^{1}H NMR** (600 MHz, DMSO): δ 7.42 (dd, *J* = 5.1, 0.9 Hz, 1H), 7.18 (d, *J* = 3.5 Hz, 1H), 7.06 (d, *J* = 3.5 Hz, 1H), 7.03 (dd, *J* = 5.0, 3.6 Hz, 1H), 6.76 (d, *J* = 3.4 Hz, 1H), 4.24 (ws, 2H - OH), 2.74 (t, *J* = 7.5 Hz, 2H), 1.64–1.53 (m, 2H), 1.49–1.39 (m, 4H), 1.25 (m, 16H) ppm. **^{13}C NMR** (151 MHz, DMSO): δ 145.0, 137.3, 134.4, 128.6, 125.8, 125.2, 124.0, 123.7, 31.5, 30.5 (d, *J* = 16.0 Hz), 29.7, 29.5, 29.4, 29.4, 29.2, 29.2, 29.0 (d, *J* = 9.0 Hz), 28.8, 27.9 (d, *J* = 136.6 Hz), 23.1 (d, *J* = 4.6 Hz) ppm. **^{31}P NMR** (243 MHz, DMSO): δ 26.72 ppm. **FTIR** (neat): *v* 2915, 2847, 1462, 1203, 983 (st), 947, 796, 685 cm^{-1}. **HRMS** (pESI) *m/z*: $[M + H]^+$ calcd for $C_{20}H_{31}O_3PS_2H$ 415.1532, found 415.1525.

13-(2,2'-Bithiophen-5-yl)tridecylphosphonic acid (TTC13P)

The same procedure and equivalents of starting materials were used as for **TTC8P** starting from 0.748 g (1.46 mmol) of **3f.** Few drops of HCl (conc.) were added to methanol/pentane solution to improve precipitation. The product was isolated as a grey powder (501 mg, 79% yield). **M.p.** = 108.9-112.6 °C. **^{1}H NMR** (600 MHz, DMSO): δ 7.43 (d, *J* = 5.1 Hz, 1H), 7.19 (d, *J* = 3.8 Hz, 1H), 7.10–7.01 (m, 2H), 6.77 (d, *J* = 3.3 Hz, 1H), 3.44 (br.s, 2x OH included in residual water in DMSO), 2.75 (t, *J* = 7.6 Hz, 2H), 1.62–1.56 (m, 2H), 1.51–1.38 (m, 4H), 1.36–1.16 (m, 18H) ppm. **^{13}C NMR** (151 MHz, DMSO): δ 145.0, 137.3, 134.4, 128.6, 125.8, 125.2, 124.0,

123.7, 31.5, 30.6, 30.5, 29.7, 29.5, 29.5, 29.4, 29.4, 29.2, 29.1, 28.8, 28.0 (d, *J* = 136.5 Hz), 23.2 (d, *J* = 4.6 Hz) ppm. **^{31}P NMR** (243 MHz, DMSO): δ 26.68 ppm. **FTIR** (neat): *v* 2914, 2846, 1472, 1007 (st), 801, 689, 541 cm^{-1}. **HRMS** (pESI) *m/z*: $[M + H]^+$ calcd for $C_{21}H_{33}O_3PS_2H$ 429.1689, found 429.1682.

^{1}H, ^{13}C and ^{31}P NMR spectra

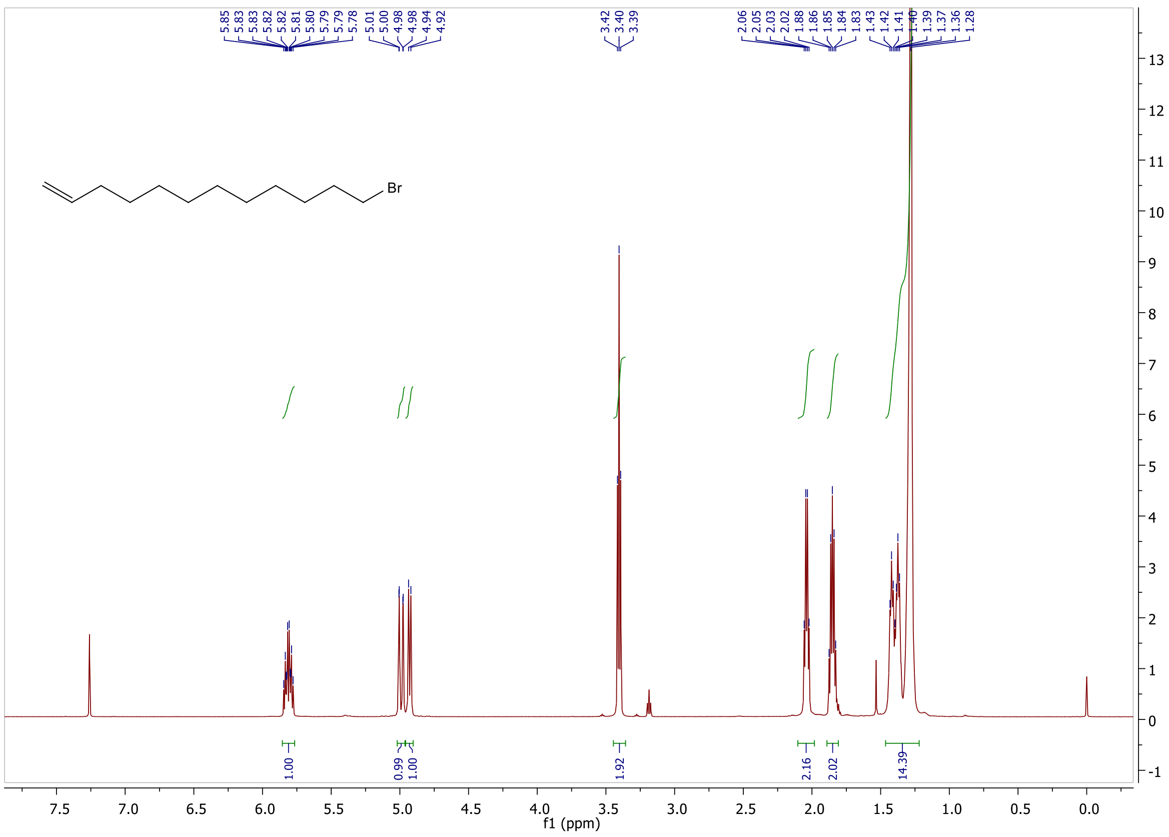

Figure S1. ^{1}H NMR (600 MHz, $CDCl_3$) of 12-bromododec-1-ene (**1e**).

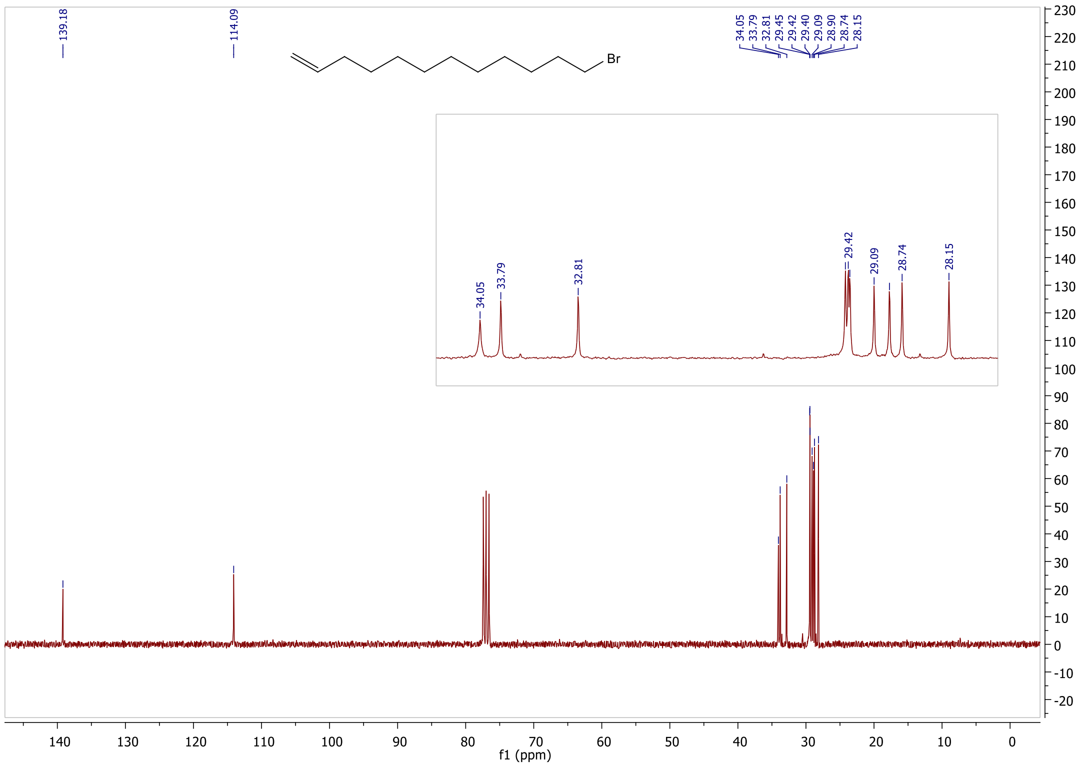

Figure S2. ^{13}C NMR (75 MHz, $CDCl_3$) of 12-bromododec-1-ene (**1e**).

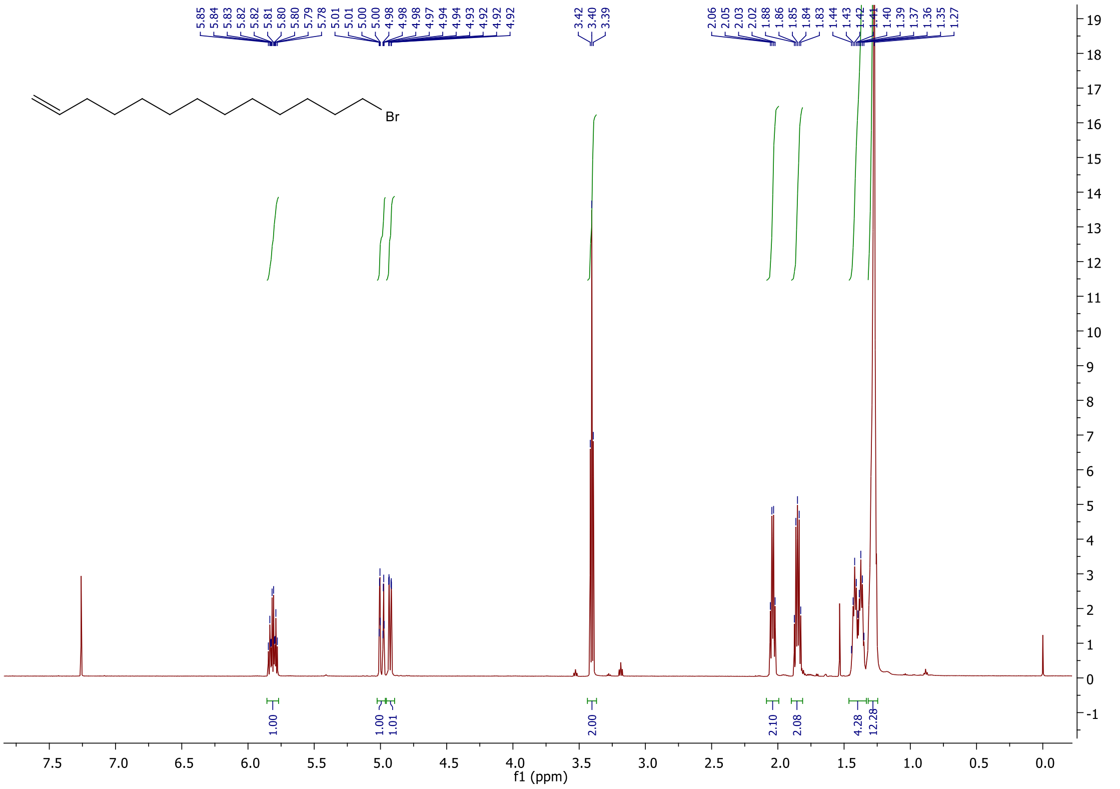

Figure S3. ^{1}H NMR (600 MHz, $CDCl_3$) of 13-bromotridec-1-ene (**1f**).

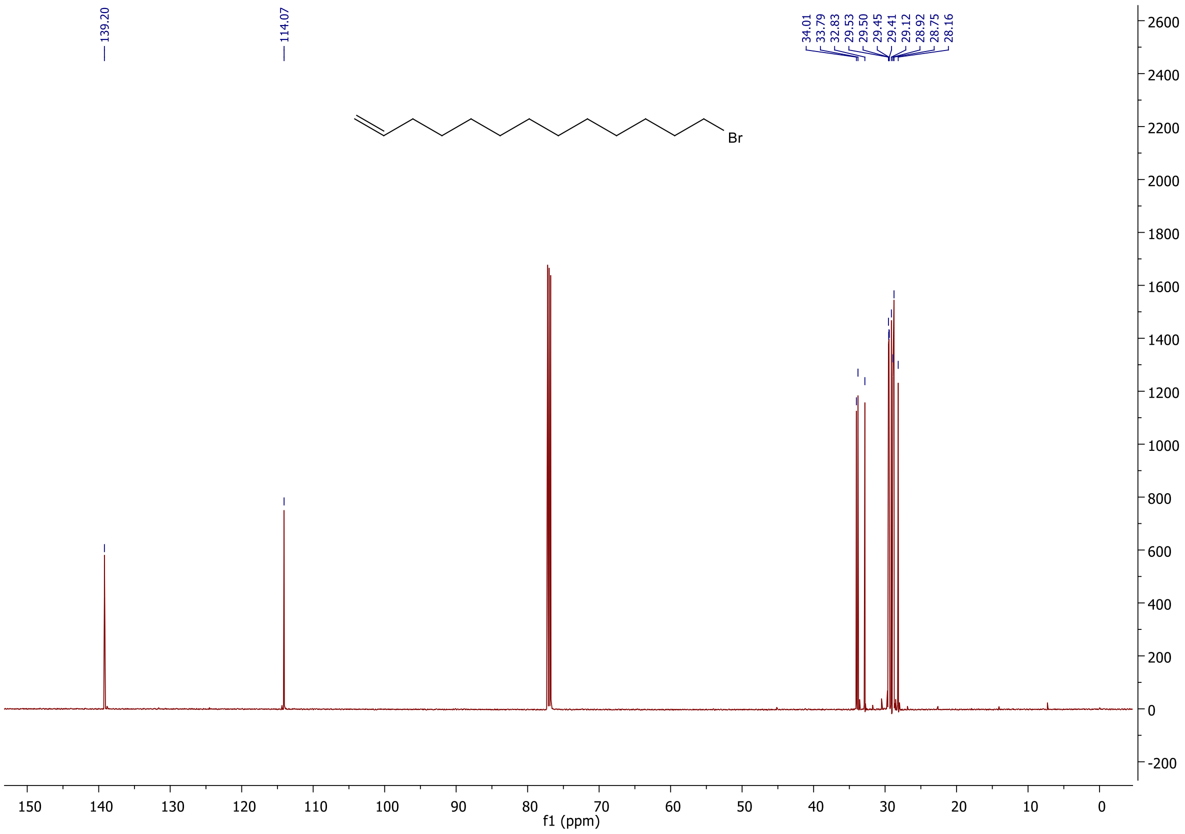

Figure S4. ^{13}C NMR (151 MHz, $CDCl_3$) of 13-bromotridec-1-ene (**1f**).

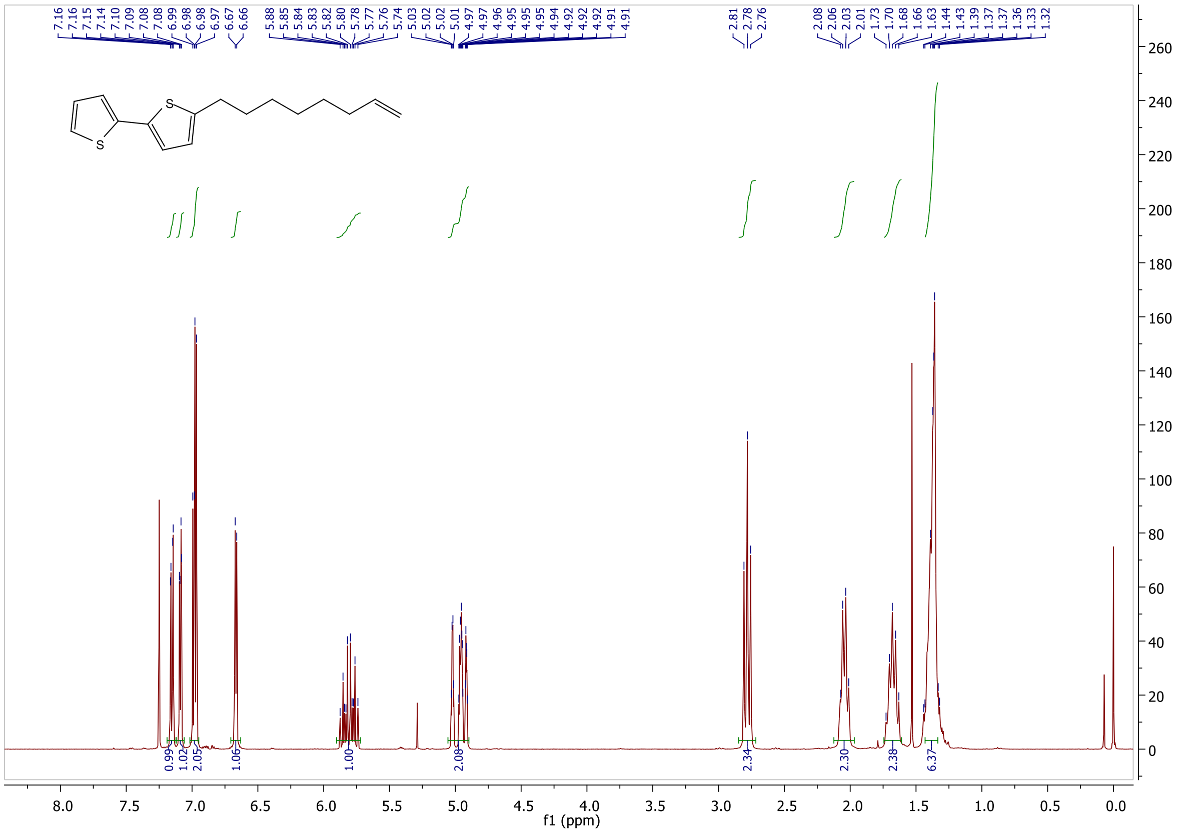

Figure S5. ^{1}H NMR (600 MHz, $CDCl_3$) of 5-(oct-7-en-1-yl)-2,2'-bithiophene (**2a**).

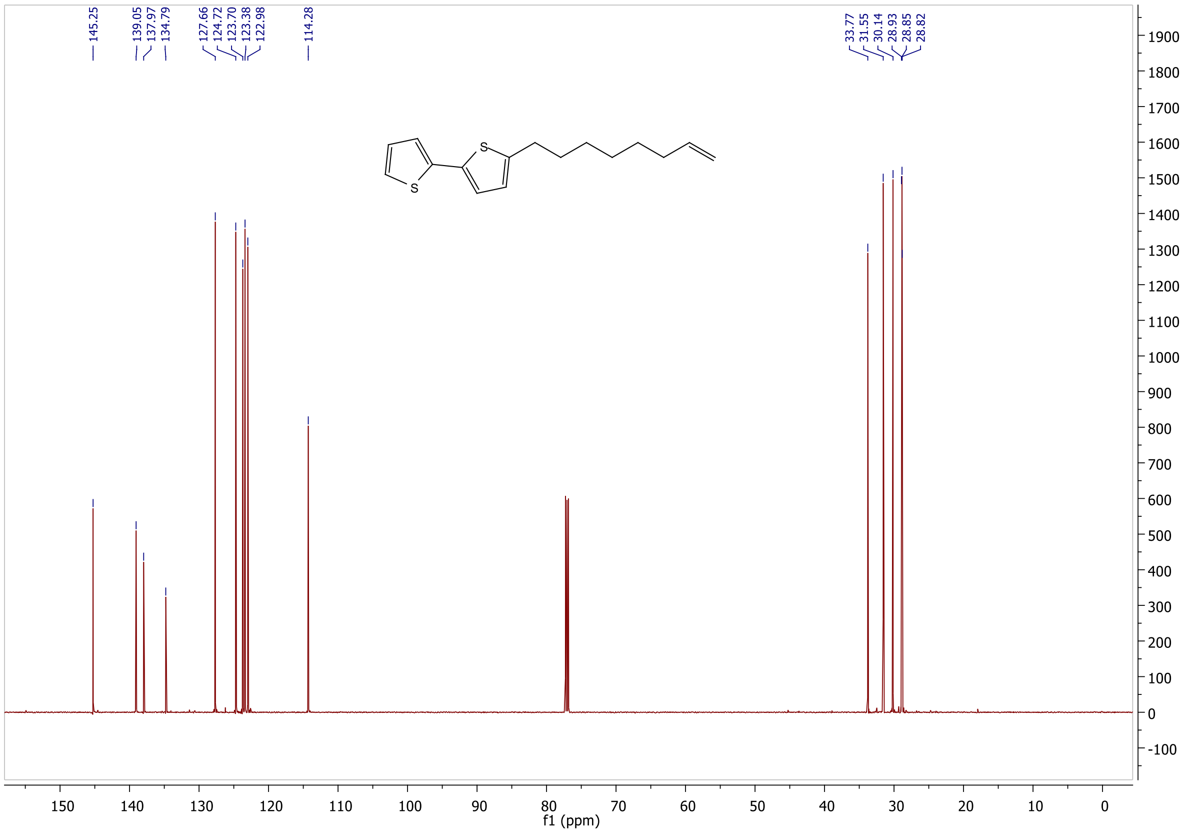

Figure S6. ^{13}C NMR (151 MHz, $CDCl_3$) of 5-(oct-7-en-1-yl)-2,2'-bithiophene (**2b**).

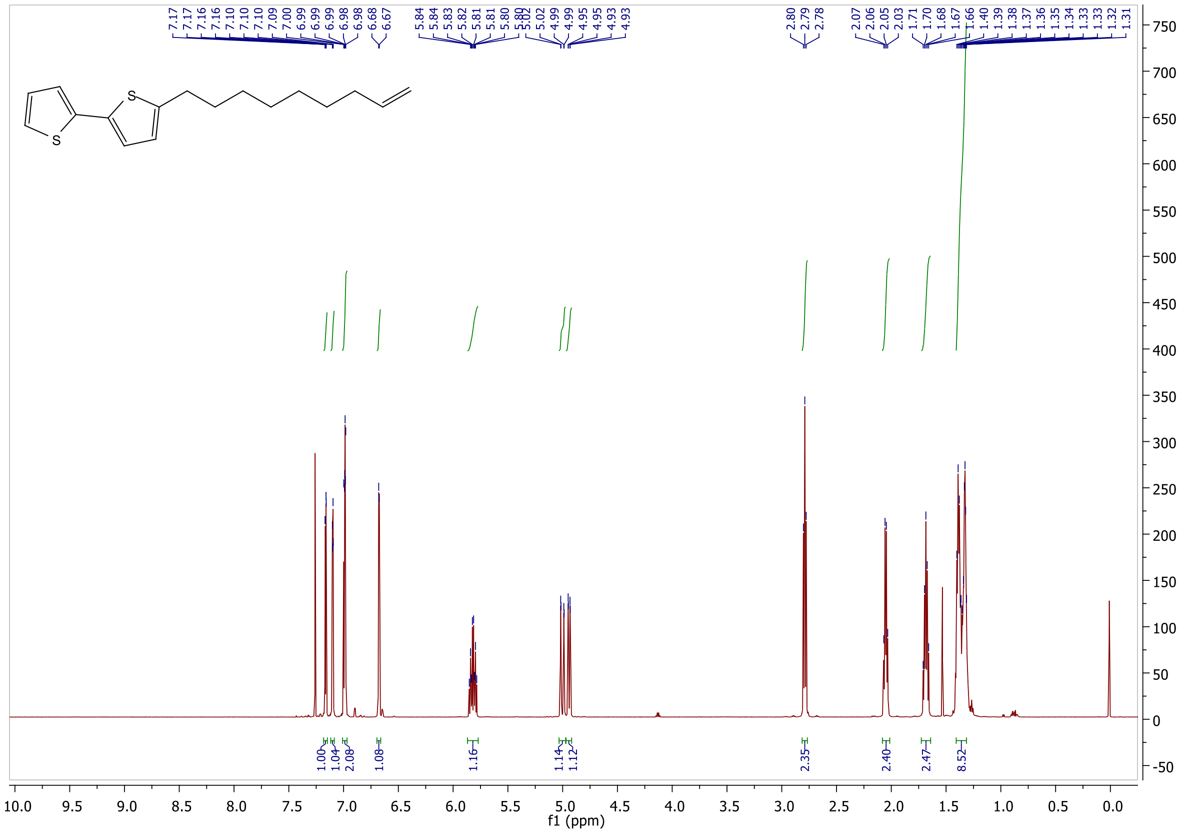

Figure S7. ^{1}H NMR (600 MHz, $CDCl_3$) of 5-(non-8-en-1-yl)-2,2'-bithiophene (**2b**).

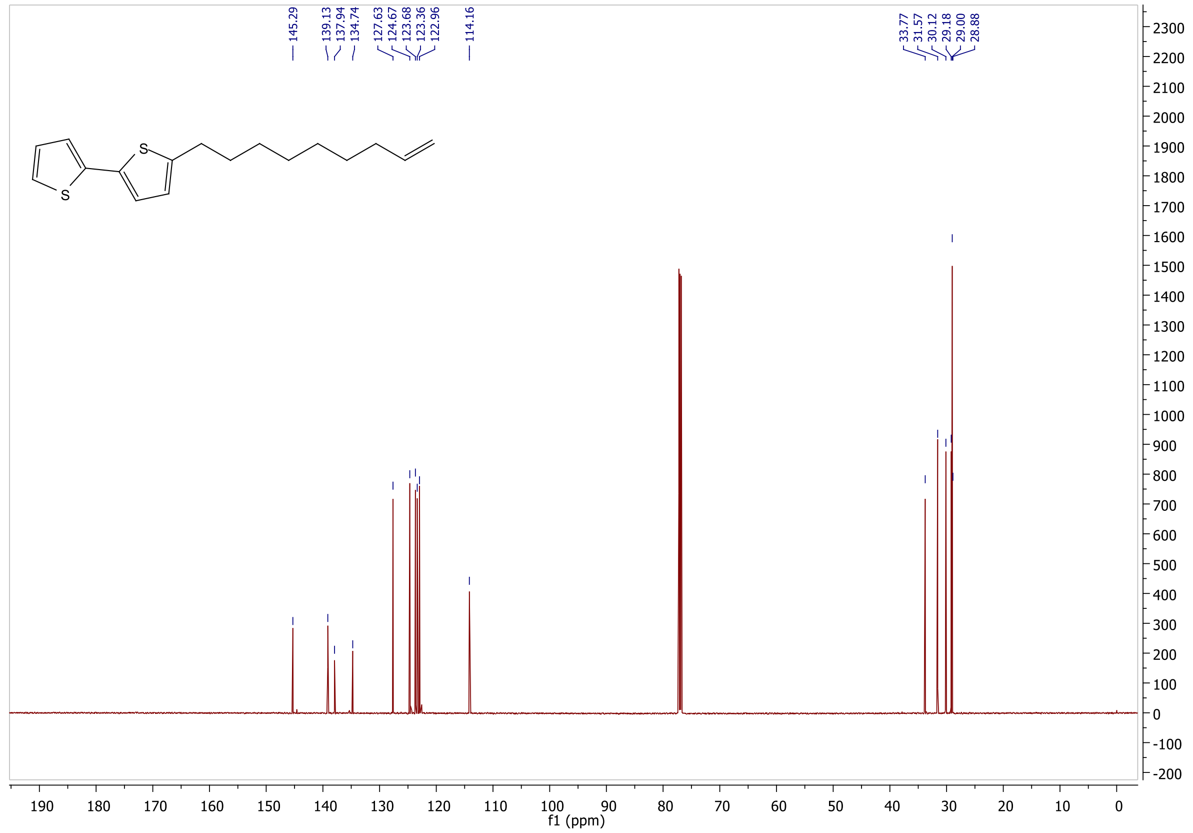

Figure S8. ^{13}C NMR (151 MHz, $CDCl_3$) of 5-(non-8-en-1-yl)-2,2'-bithiophene (**2b**).

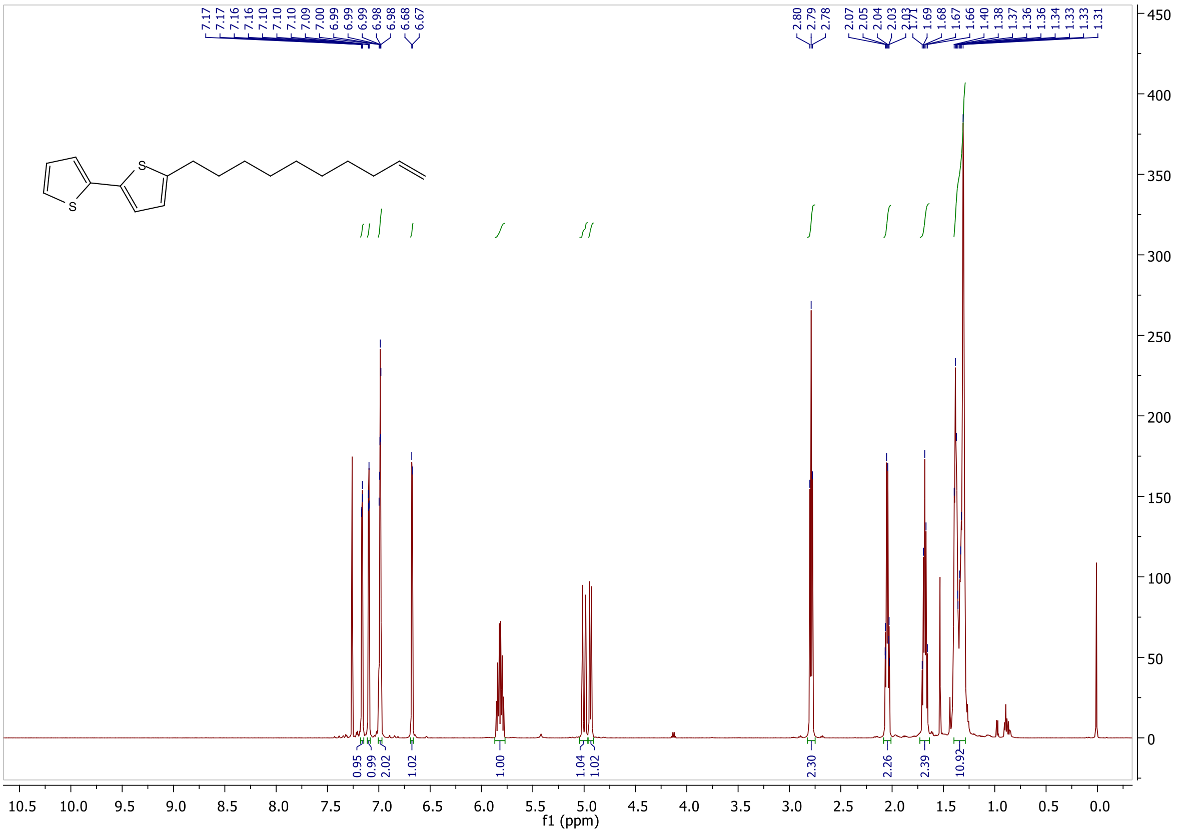

Figure S9. ^{1}H NMR (600 MHz, $CDCl_3$) of 5-(dec-9-en-1-yl)-2,2'-bithiophene (**2c**).

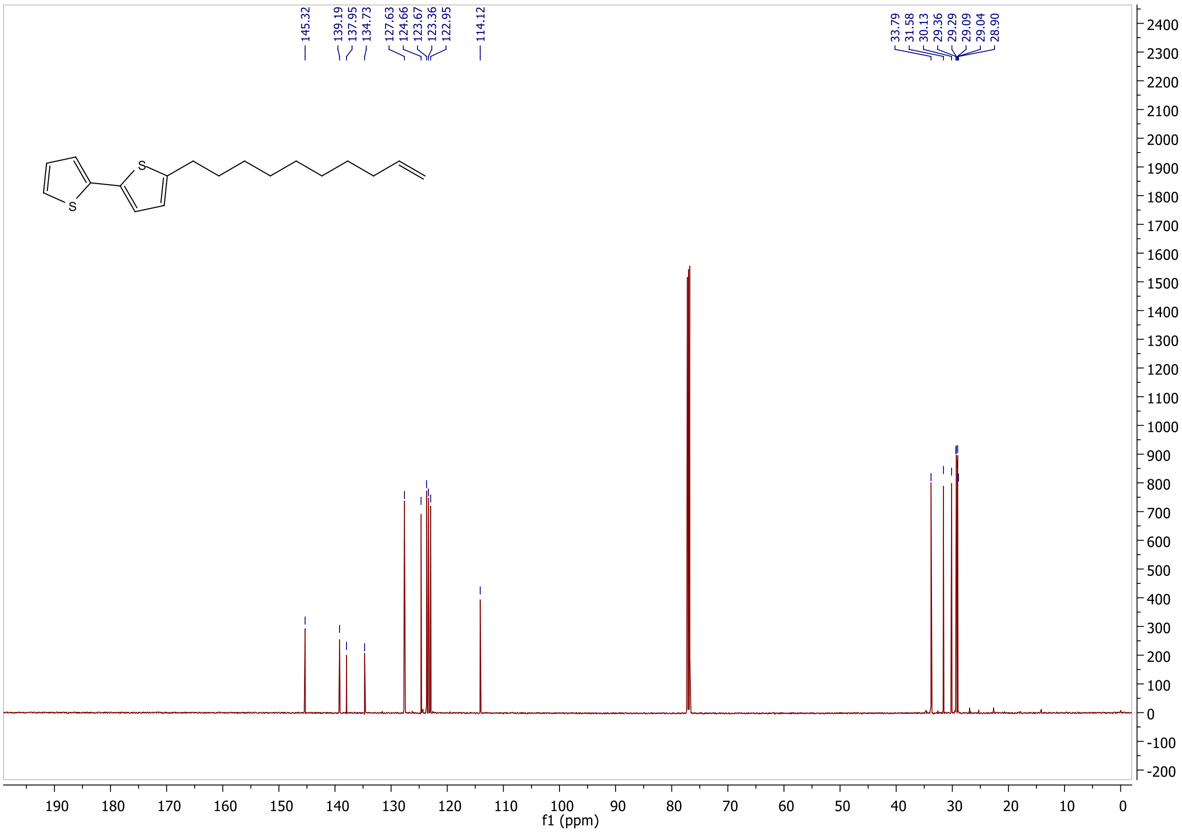

Figure S10. ^{13}C NMR (151 MHz, $CDCl_3$) of 5-(dec-9-en-1-yl)-2,2'-bithiophene (**2c**).

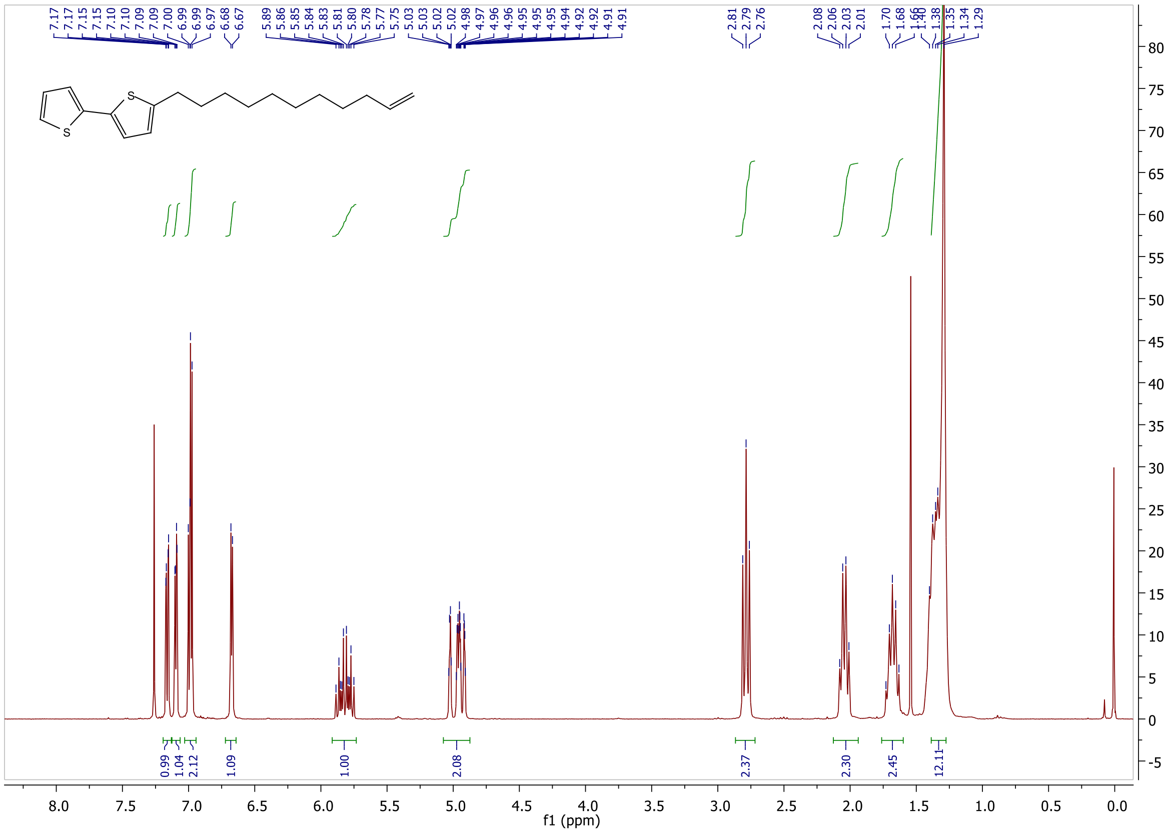

Figure S11. ^{1}H NMR (600 MHz, $CDCl_3$) of 5-(undec-10-en-1-yl)-2,2'-bithiophene (**2d**).

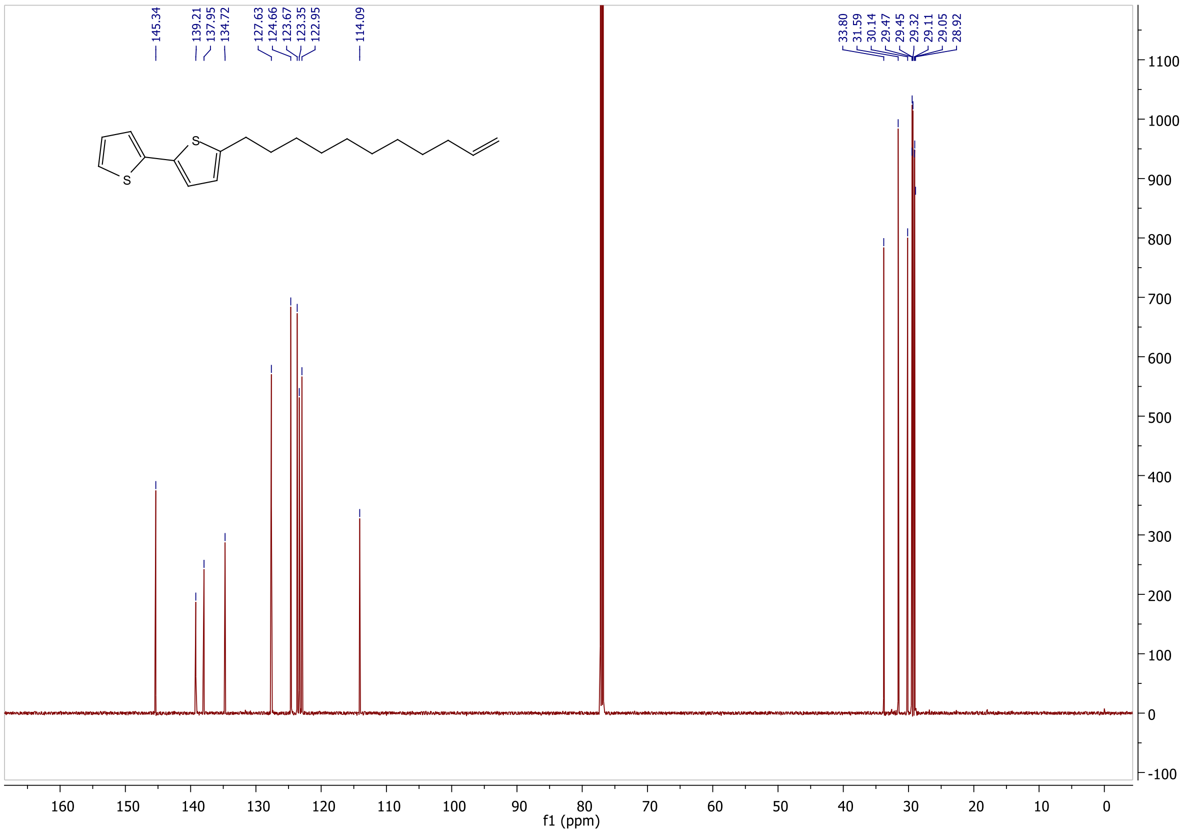

Figure S12. ^{13}C NMR (151 MHz, $CDCl_3$) of 5-(undec-10-en-1-yl)-2,2'-bithiophene (**2d**).

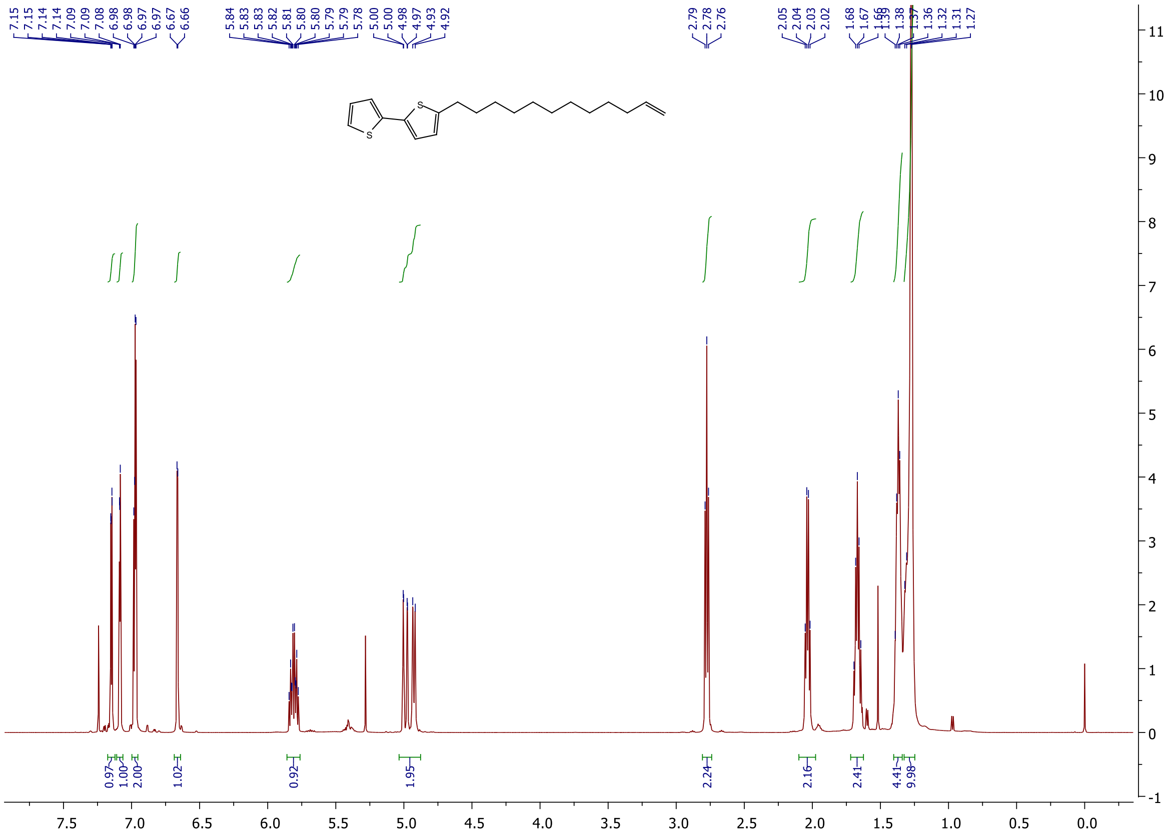

Figure S13. ^{1}H NMR (600 MHz, $CDCl_3$) of 5-(dodec-11-en-1-yl)-2,2'-bithiophene (**2e**).

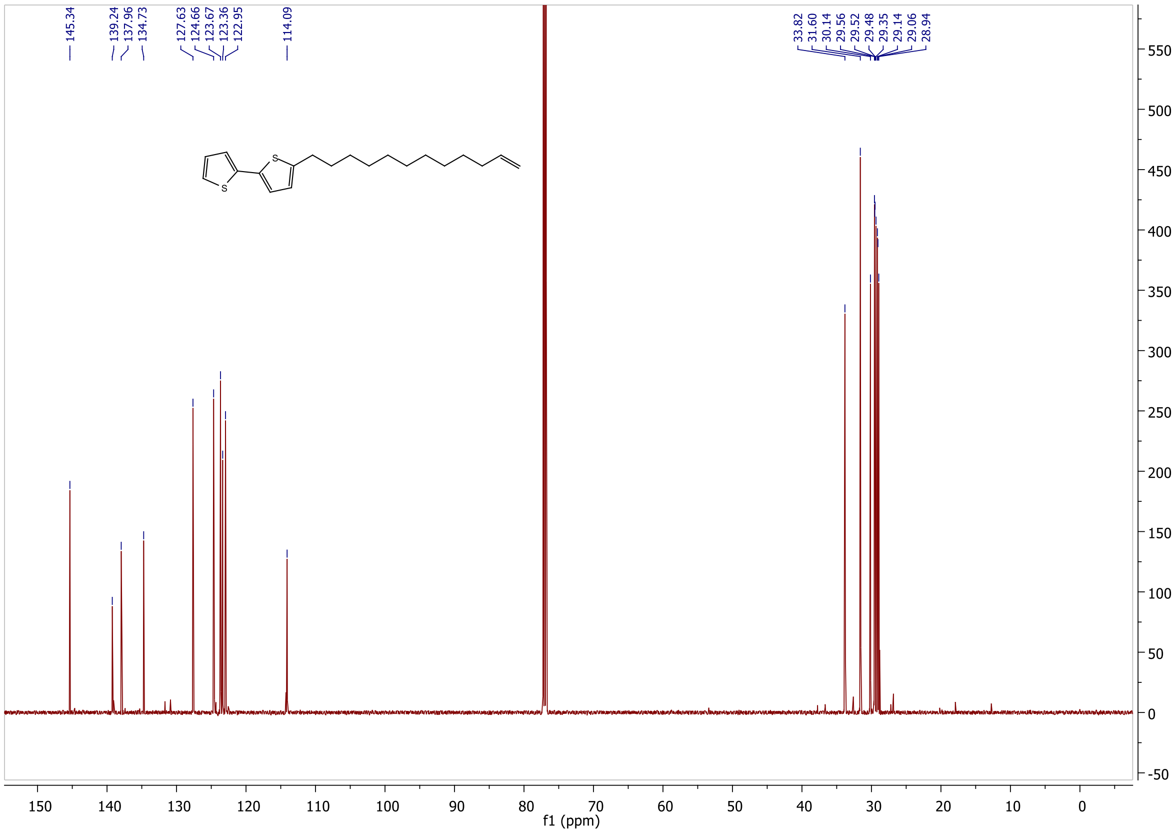

Figure S14. ^{13}C NMR (151 MHz, $CDCl_3$) of 5-(dodec-11-en-1-yl)-2,2'-bithiophene (**2e**).

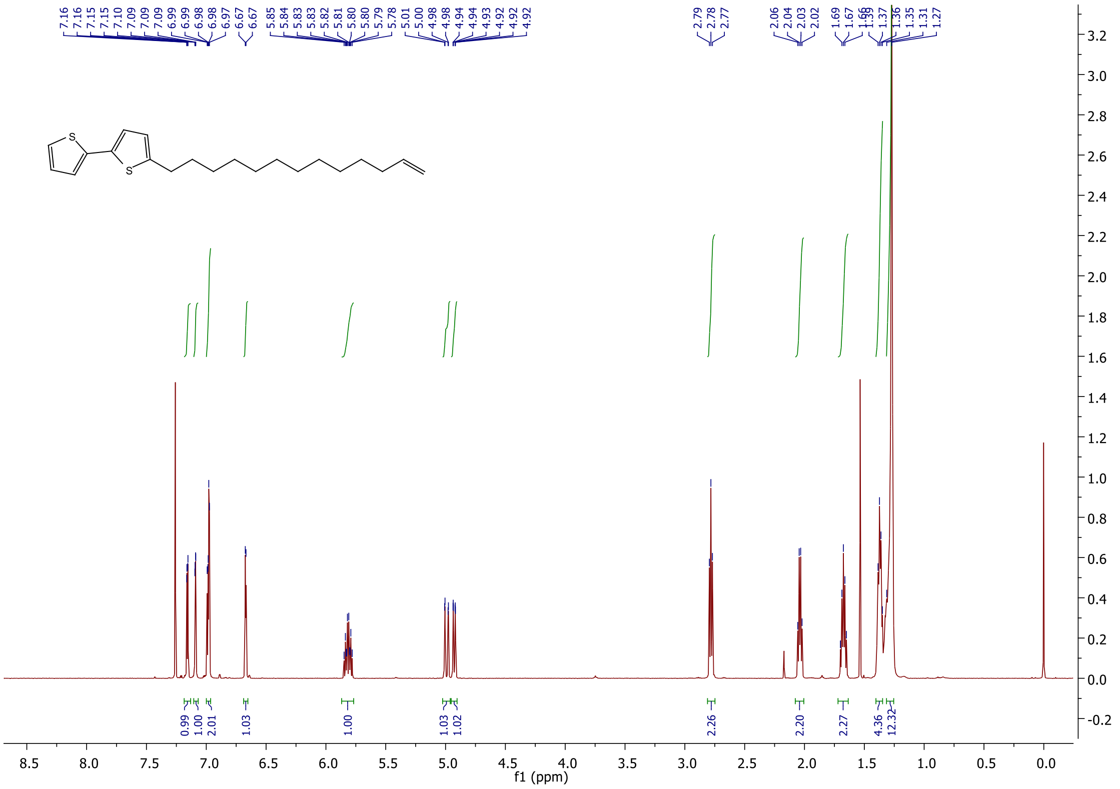

Figure S15. ^{1}H NMR (600 MHz, $CDCl_3$) of 5-(tridec-12-en-1-yl)-2,2'-bithiophene (**2f**).

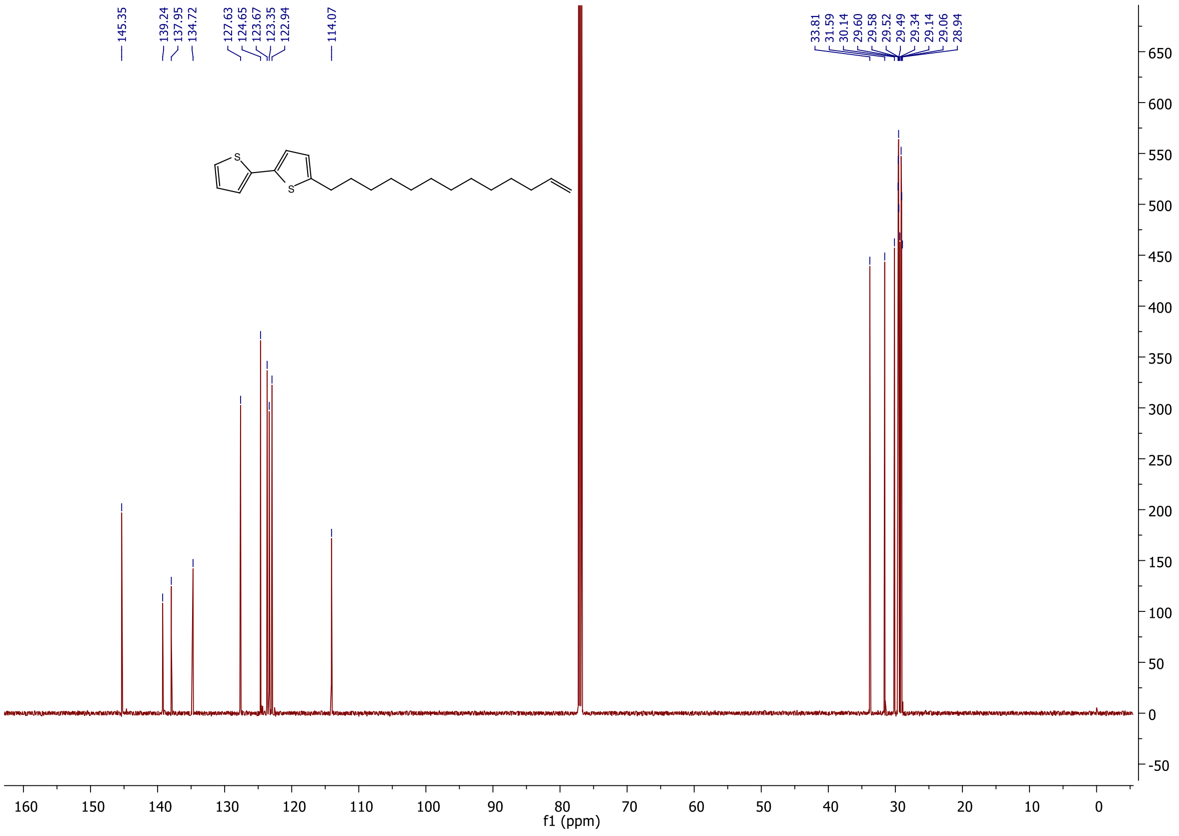

Figure S16. ^{13}C NMR (151 MHz, $CDCl_3$) of 5-(tridec-12-en-1-yl)-2,2'-bithiophene (**2f**).

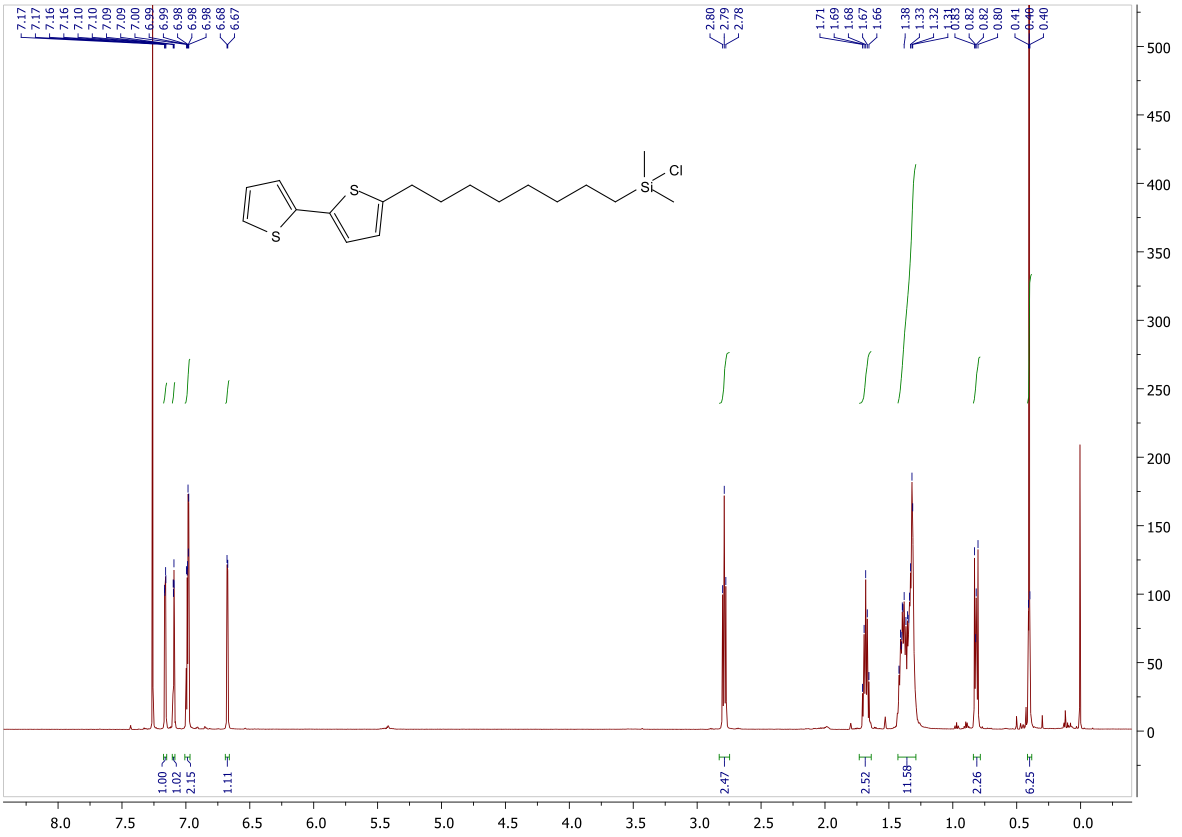

Figure S17. ^{1}H NMR (600 MHz, $CDCl_3$) of [8-(2,2'-bithiophen-5-yl)octyl]chlorodimethylsilane (**TTC8Si**).

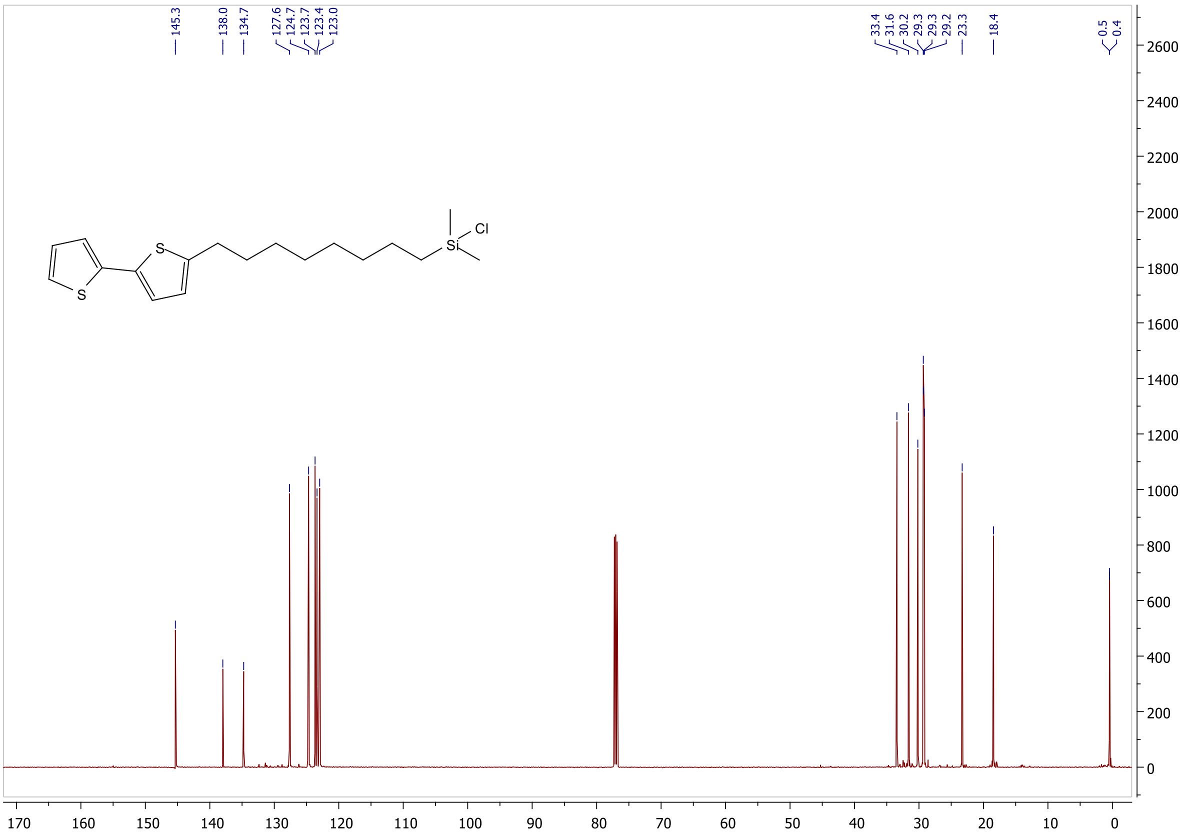

Figure S18. ^{13}C NMR (151 MHz, $CDCl_3$) of [8-(2,2'-bithiophen-5-yl)octyl]chlorodimethylsilane (**TTC8Si**).

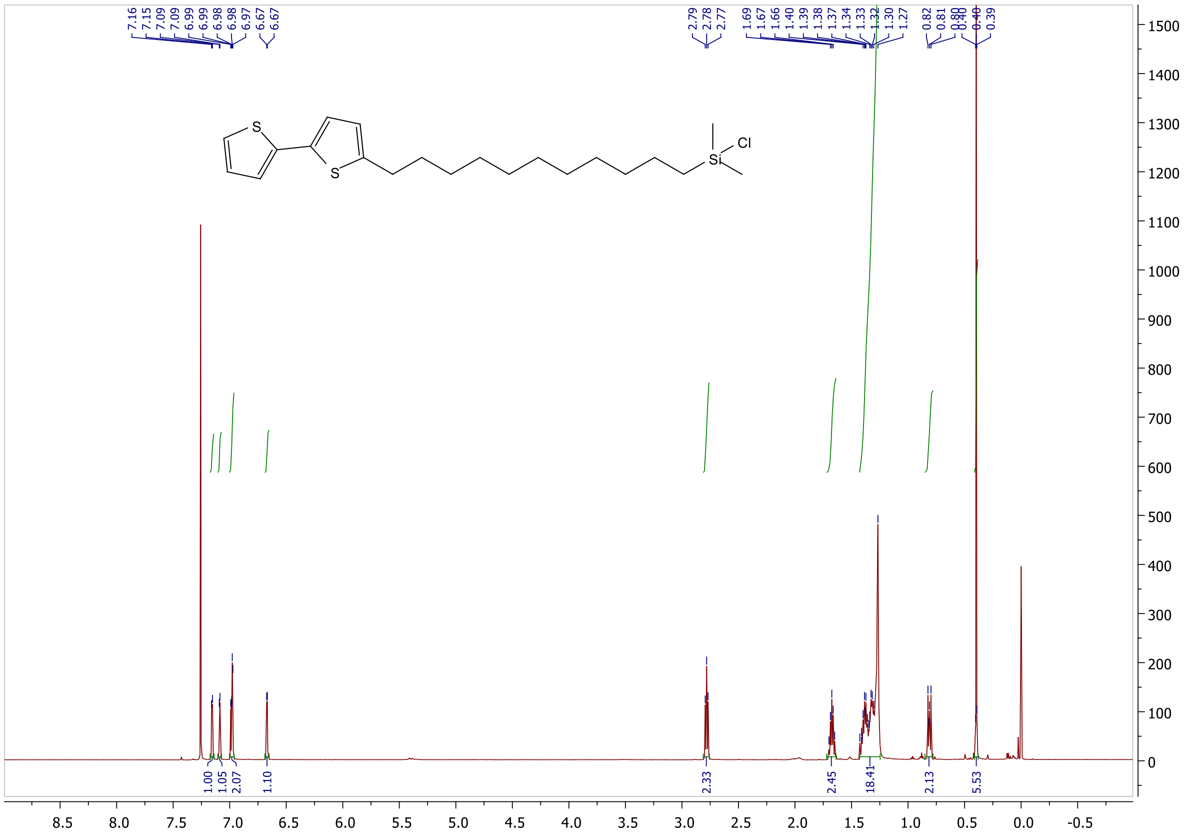

Figure S19. ^{1}H NMR (600 MHz, $CDCl_3$) of [11-(2,2'-bithiophen-5-yl)undecyl]chlorodimethylsilane (**TTC11Si**).

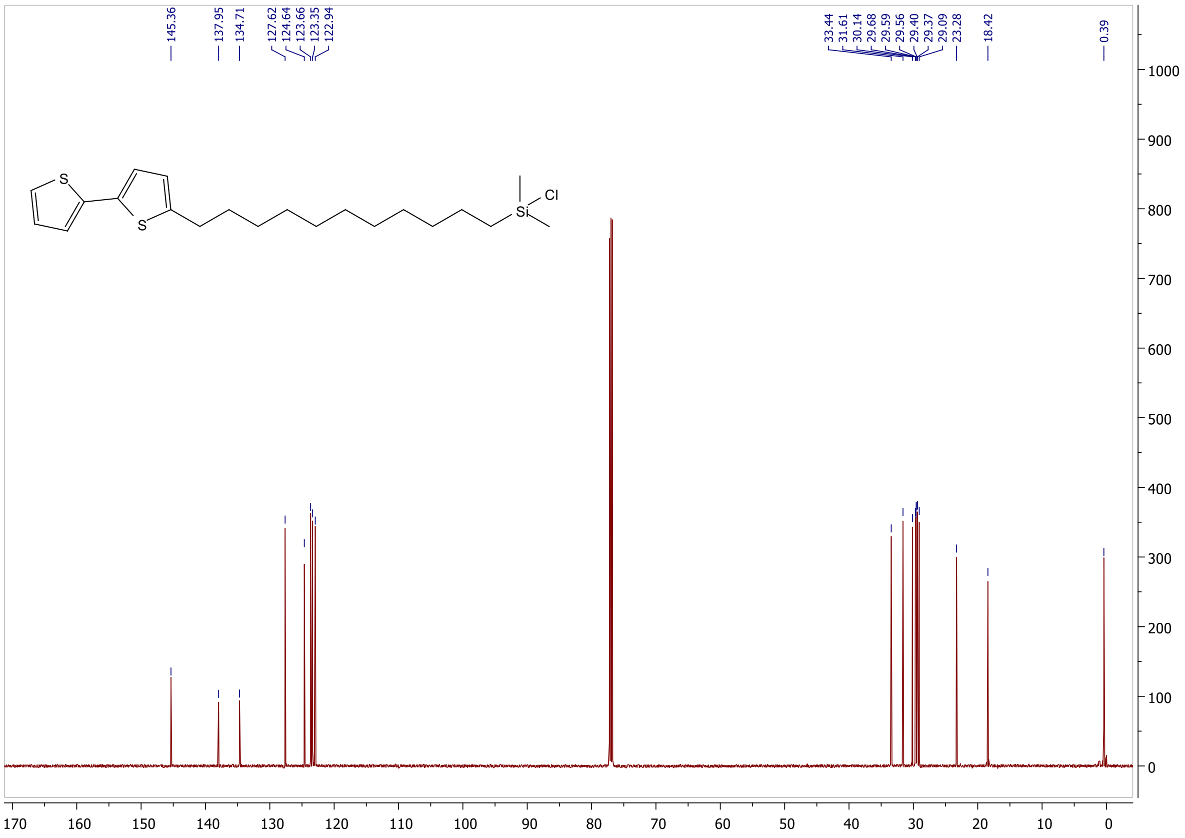

Figure S20. ^{13}C NMR (151 MHz, $CDCl_3$) of [11-(2,2'-bithiophen-5-yl)undecyl]chlorodimethylsilane (**TTC11Si**).

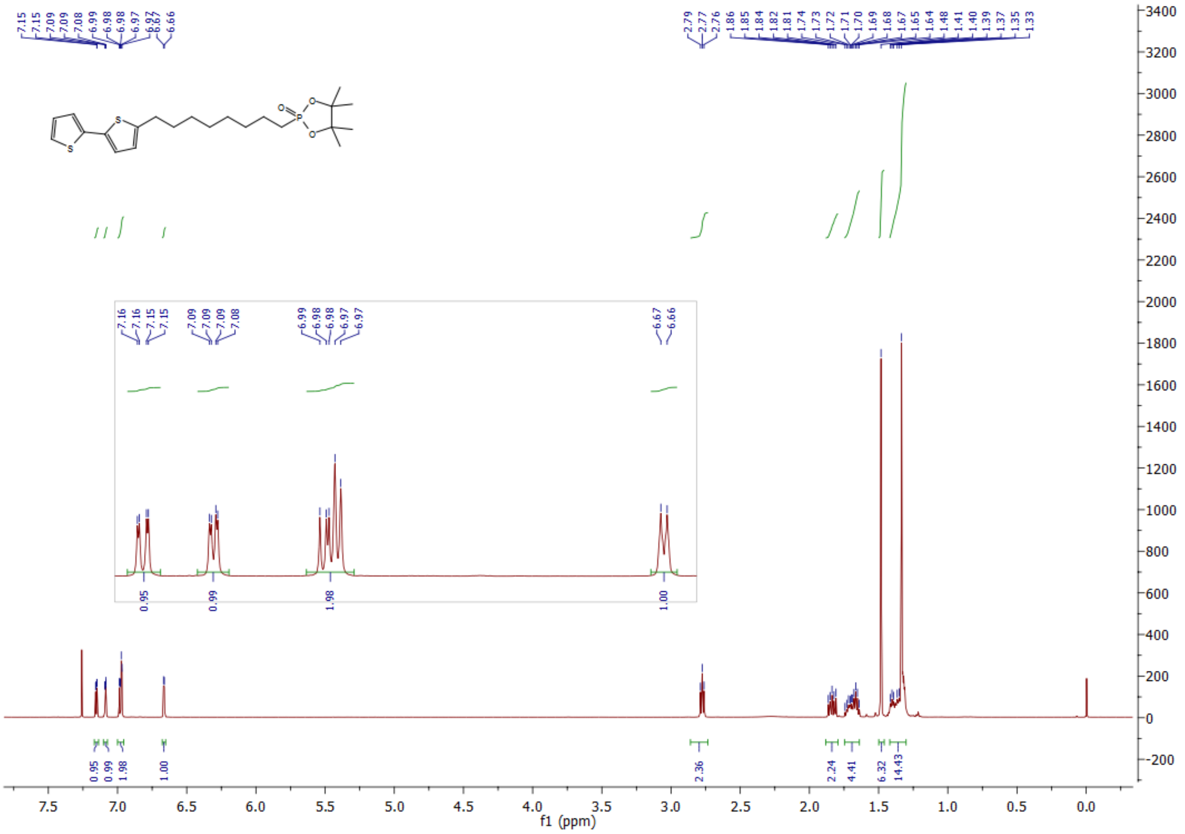

Figure S21. ^{1}H NMR (600 MHz, $CDCl_3$) of 2-[8-(2,2'-bithiophen-5-yl)octyl]-4,4,5,5-tetramethyl-1,3,2-dioxaphospholane (**3a**).

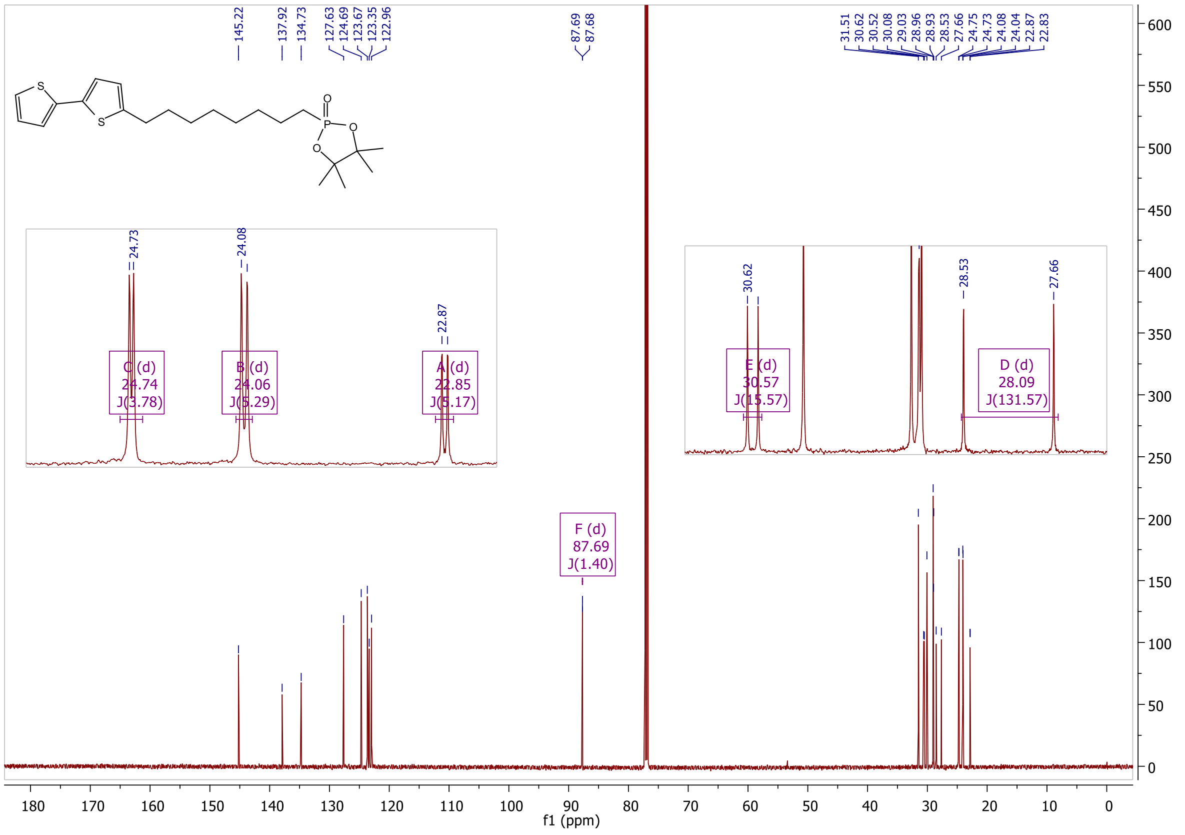

Figure S22. ^{13}C NMR (151 MHz, $CDCl_3$) of 2-[8-(2,2'-bithiophen-5-yl)octyl]-4,4,5,5-tetramethyl-1,3,2-dioxaphospholane 2-oxide (**3a**).

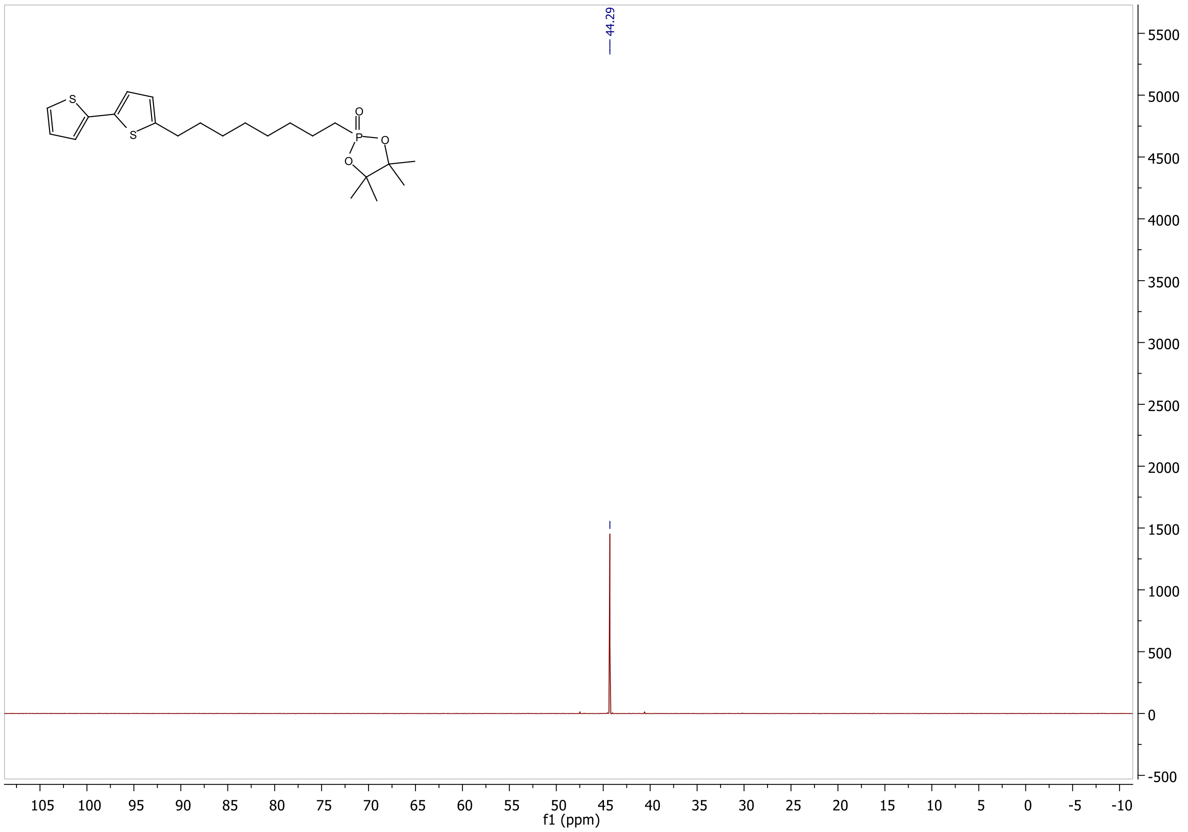

Figure S23. ^{31}P NMR (243 MHz, $CDCl_3$) of 2-[8-(2,2'-bithiophen-5-yl)octyl]-4,4,5,5-tetramethyl-1,3,2-dioxaphospholane 2-oxide (**3a**).

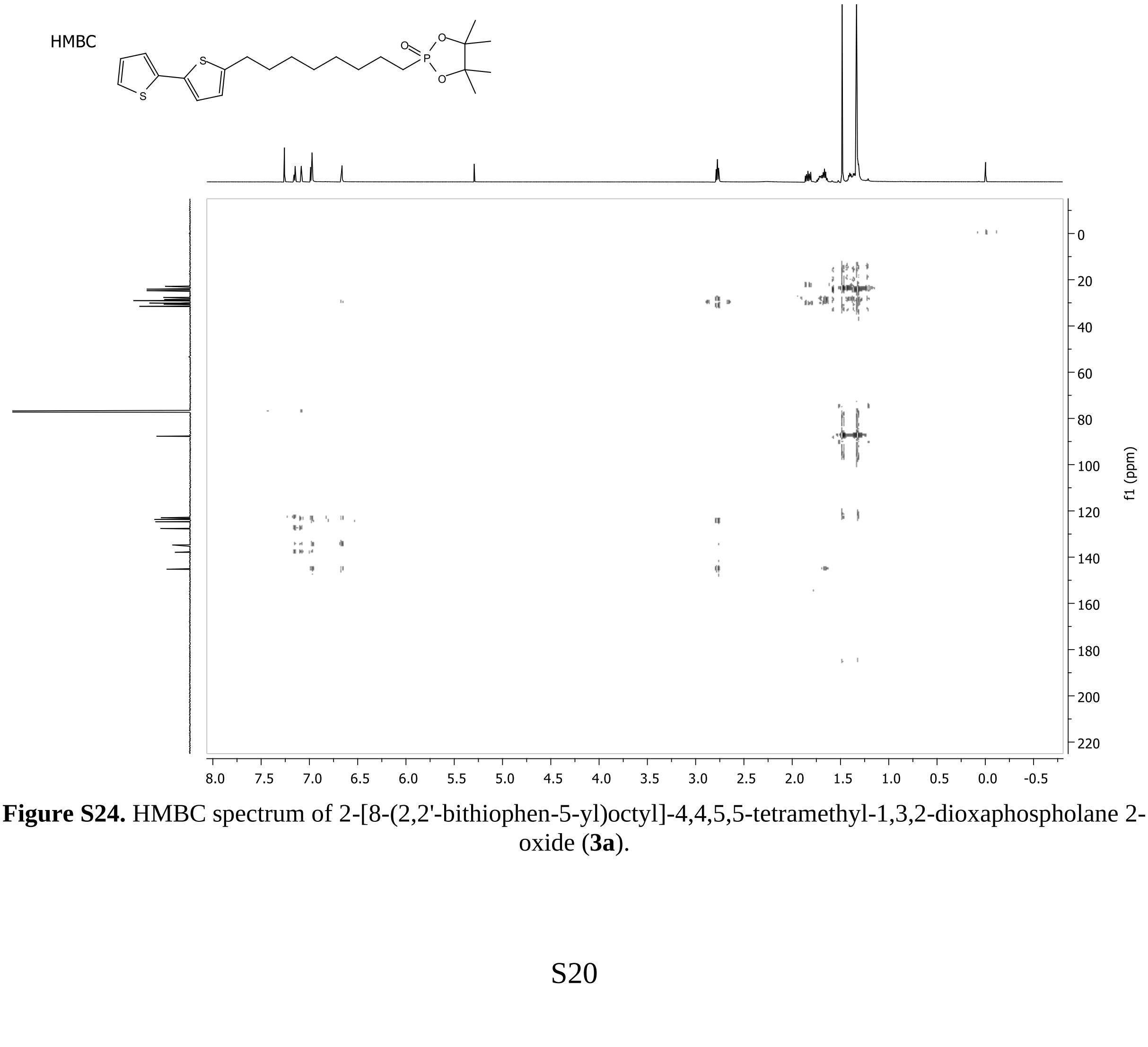

Figure S24. HMBC spectrum of 2-[8-(2,2'-bithiophen-5-yl)octyl]-4,4,5,5-tetramethyl-1,3,2-dioxaphospholane 2-oxide (**3a**).

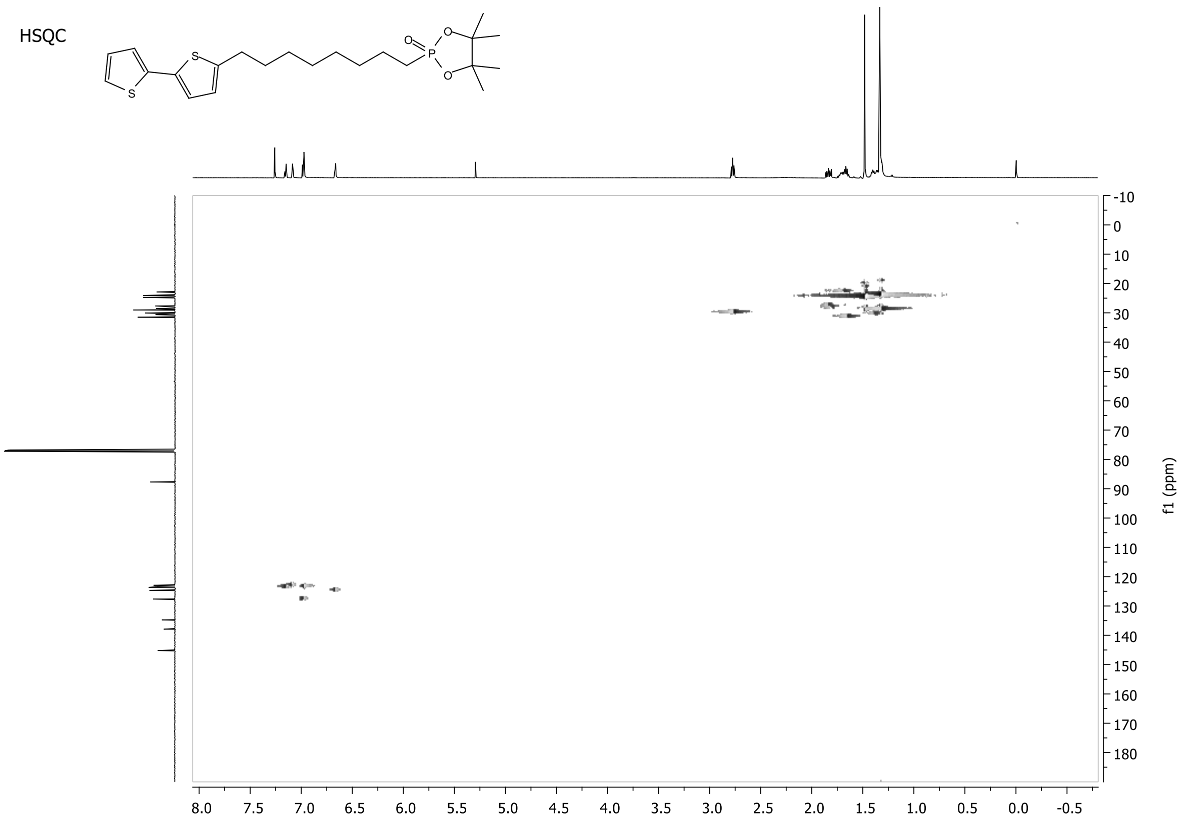

Figure S25. HSQC spectrum of of 2-[8-(2,2'-bithiophen-5-yl)octyl]-4,4,5,5-tetramethyl-1,3,2-dioxaphospholane 2-oxide (**3a**).

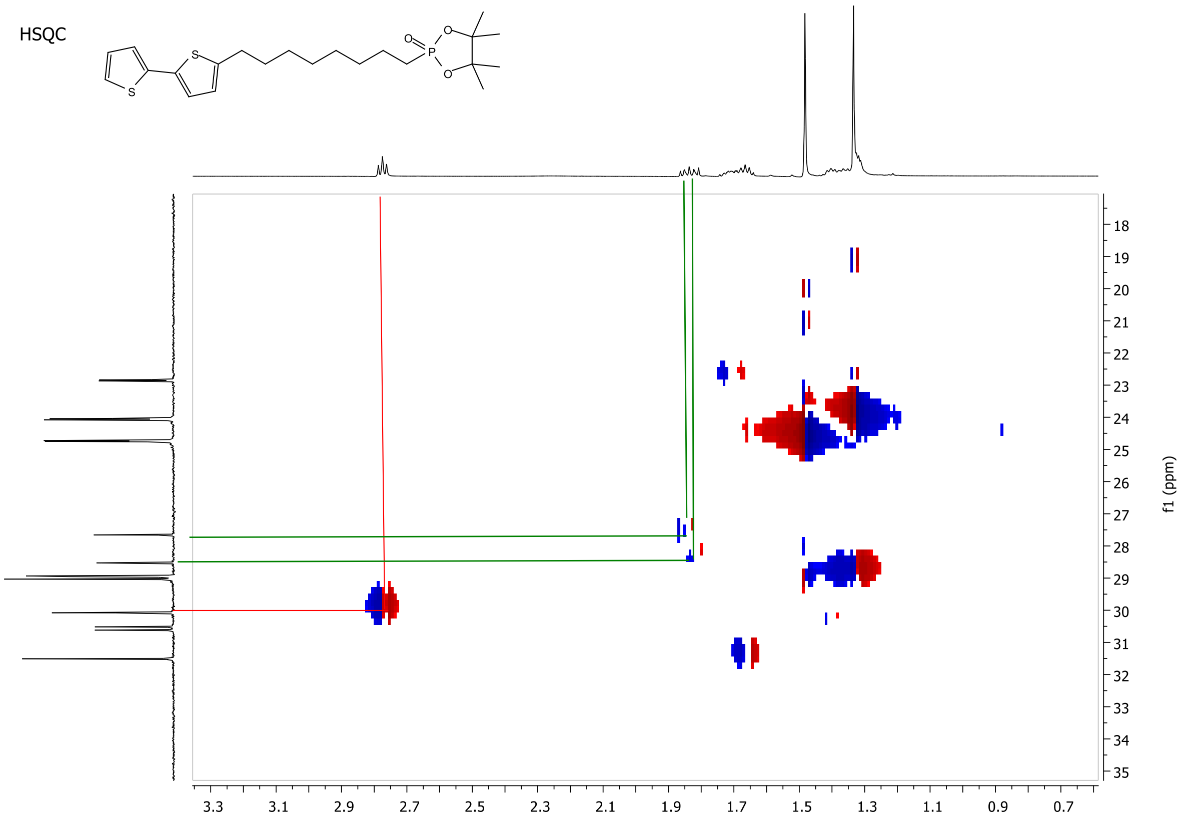

Figure S26. Zoomed HSQC spectrum of 2-[8-(2,2'-bithiophen-5-yl)octyl]-4,4,5,5-tetramethyl-1,3,2-dioxaphospholane 2-oxide (**3a**).

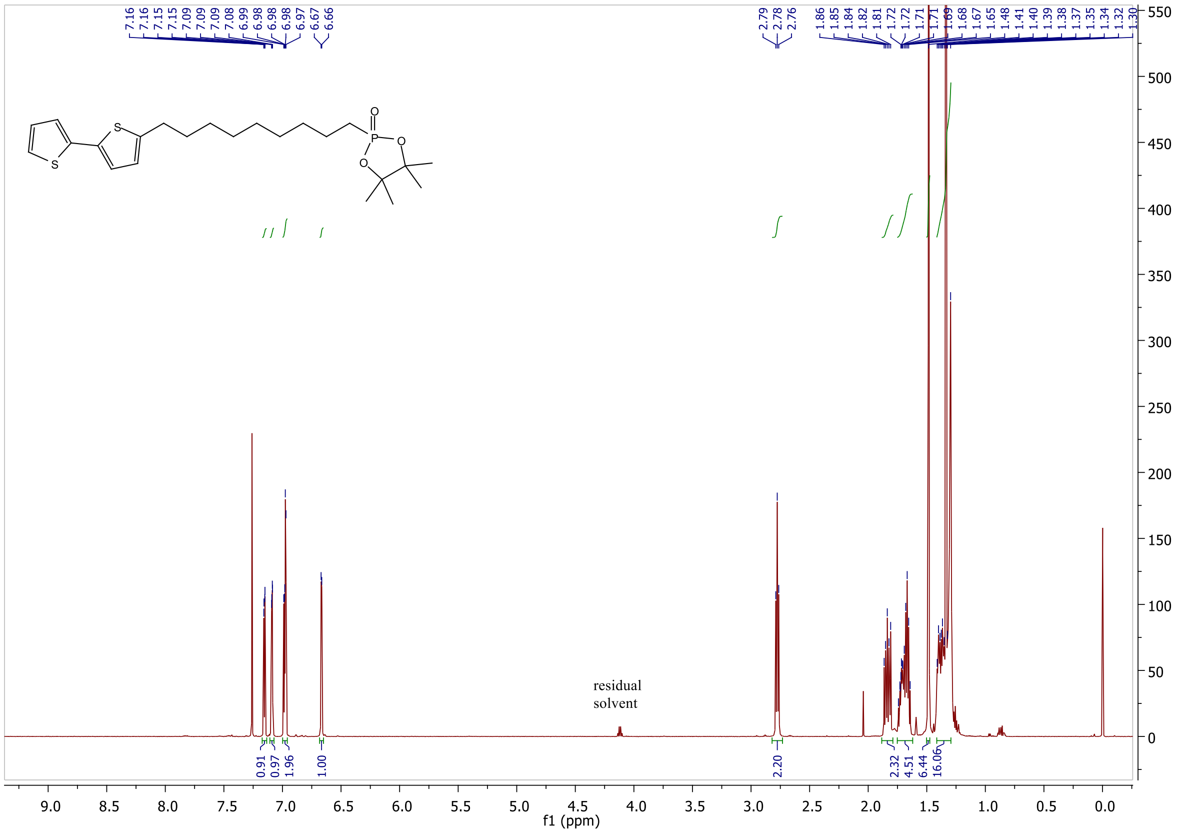

Figure S27. ^{1}H NMR (600 MHz, $CDCl_3$) of 2-[9-(2,2'-bithiophen-5-yl)nonyl]-4,4,5,5-tetramethyl-1,3,2-dioxaphospholane 2-oxide (**3b**).

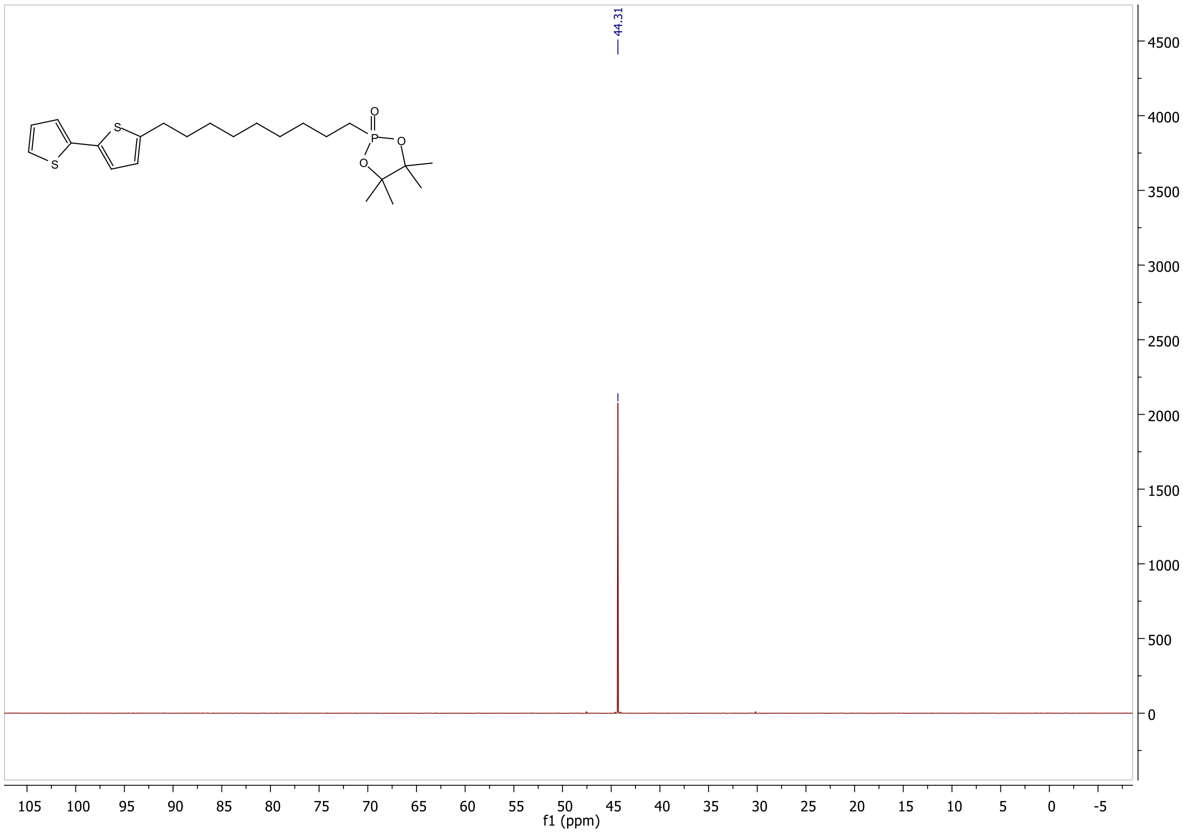

Figure S28. ^{31}P NMR (243 MHz, $CDCl_3$) 2-[9-(2,2'-bithiophen-5-yl)nonyl]-4,4,5,5-tetramethyl-1,3,2-dioxaphospholane 2-oxide (**3b**).

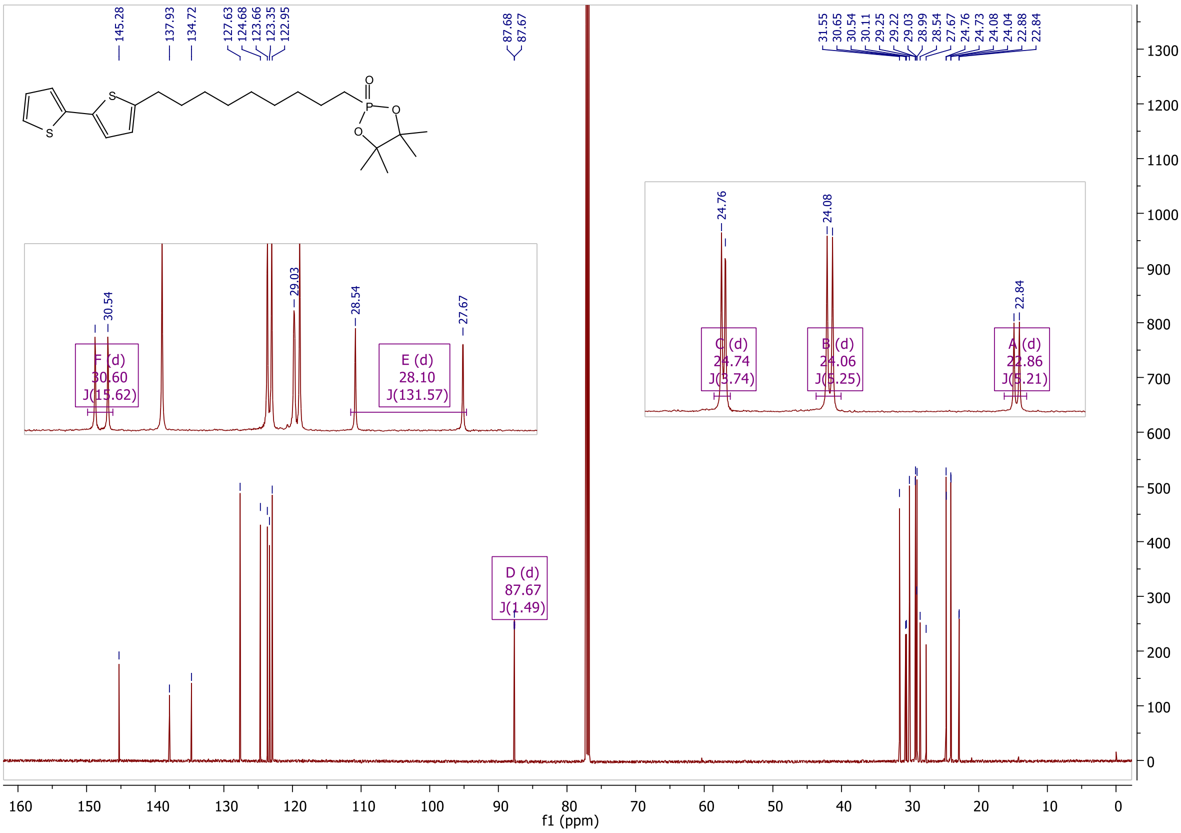

Figure S29. ^{13}C NMR (151 MHz, $CDCl_3$) 2-[9-(2,2'-bithiophen-5-yl)nonyl]-4,4,5,5-tetramethyl-1,3,2-dioxaphospholane 2-oxide (**3b**).

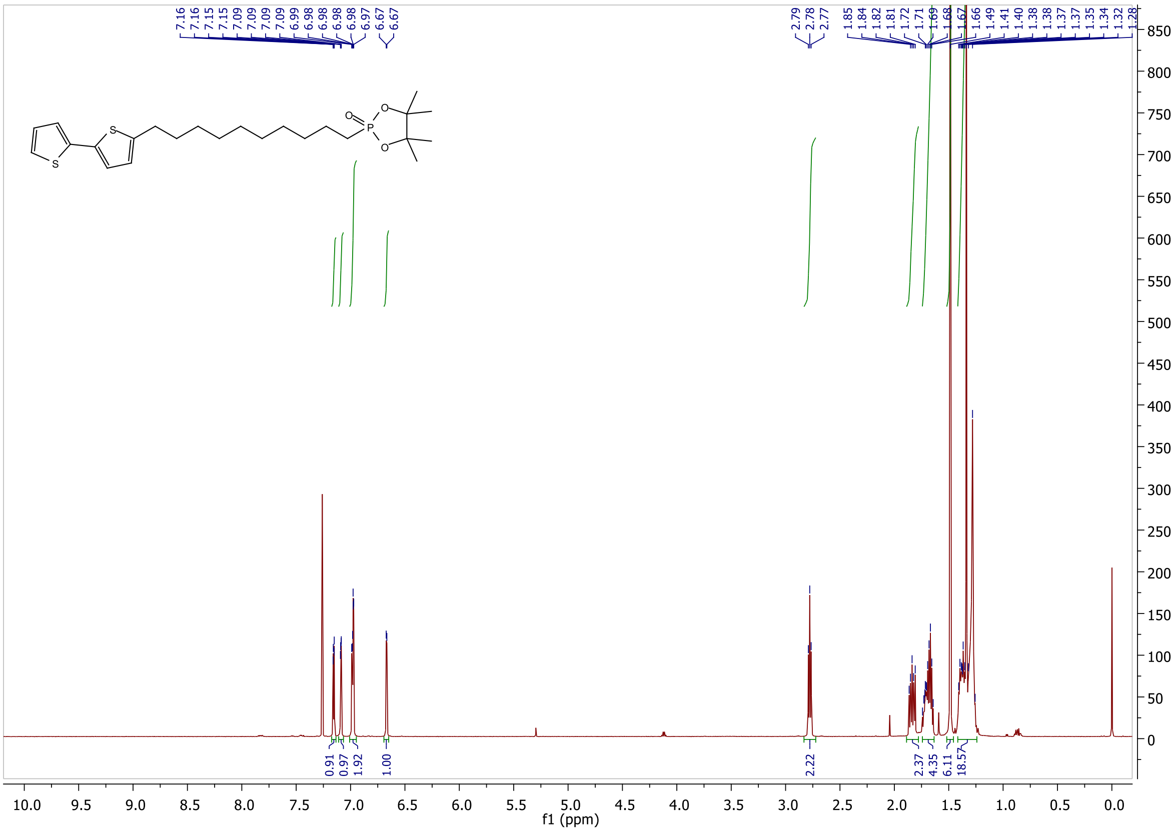

Figure S30. ^{1}H NMR (600 MHz, $CDCl_3$) of 2-[10-(2,2'-bithiophen-5-yl)decyl]-4,4,5,5-tetramethyl-1,3,2-dioxaphospholane 2-oxide (**3c**).

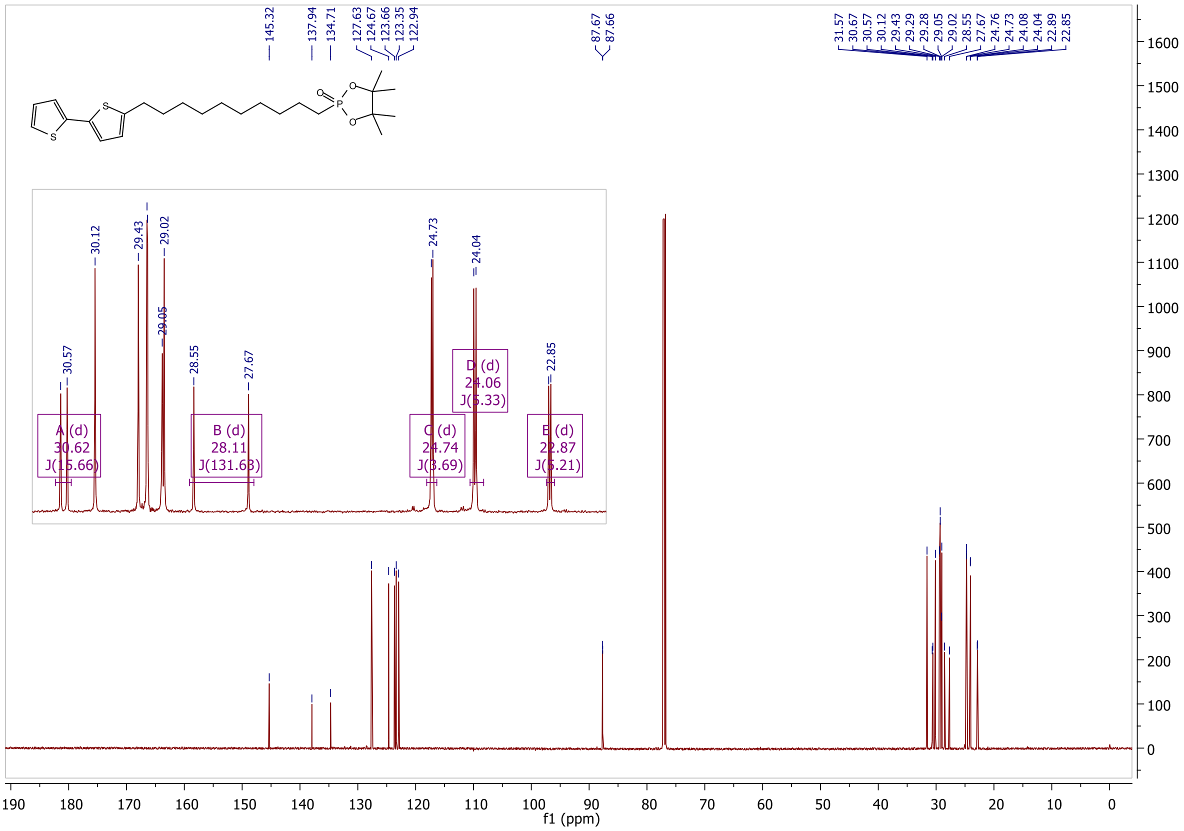

Figure S31. ^{13}C NMR (151 MHz, $CDCl_3$) 2-[10-(2,2'-bithiophen-5-yl)decyl]-4,4,5,5-tetramethyl-1,3,2-dioxaphospholane 2-oxide (**3c**).

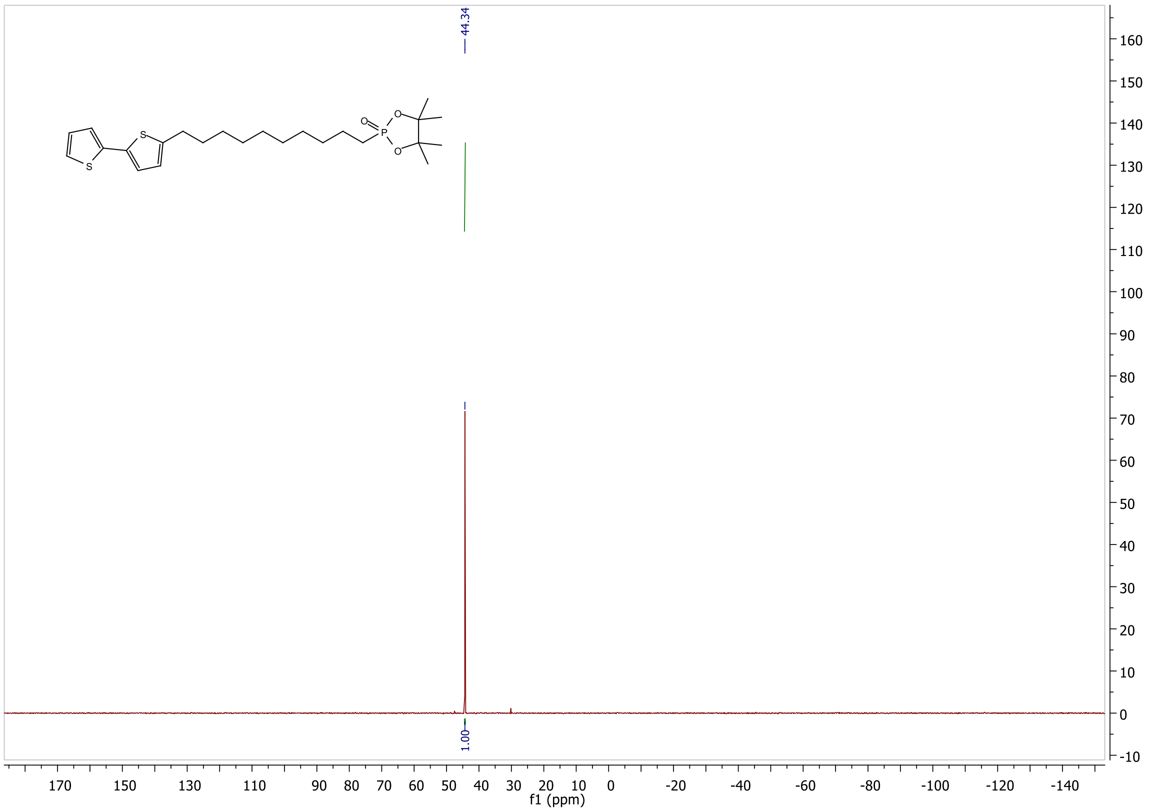

Figure S32. ^{31}P NMR (243 MHz, $CDCl_3$) 2-[10-(2,2'-bithiophen-5-yl)decyl]-4,4,5,5-tetramethyl-1,3,2-dioxaphospholane 2-oxide (**3c**).

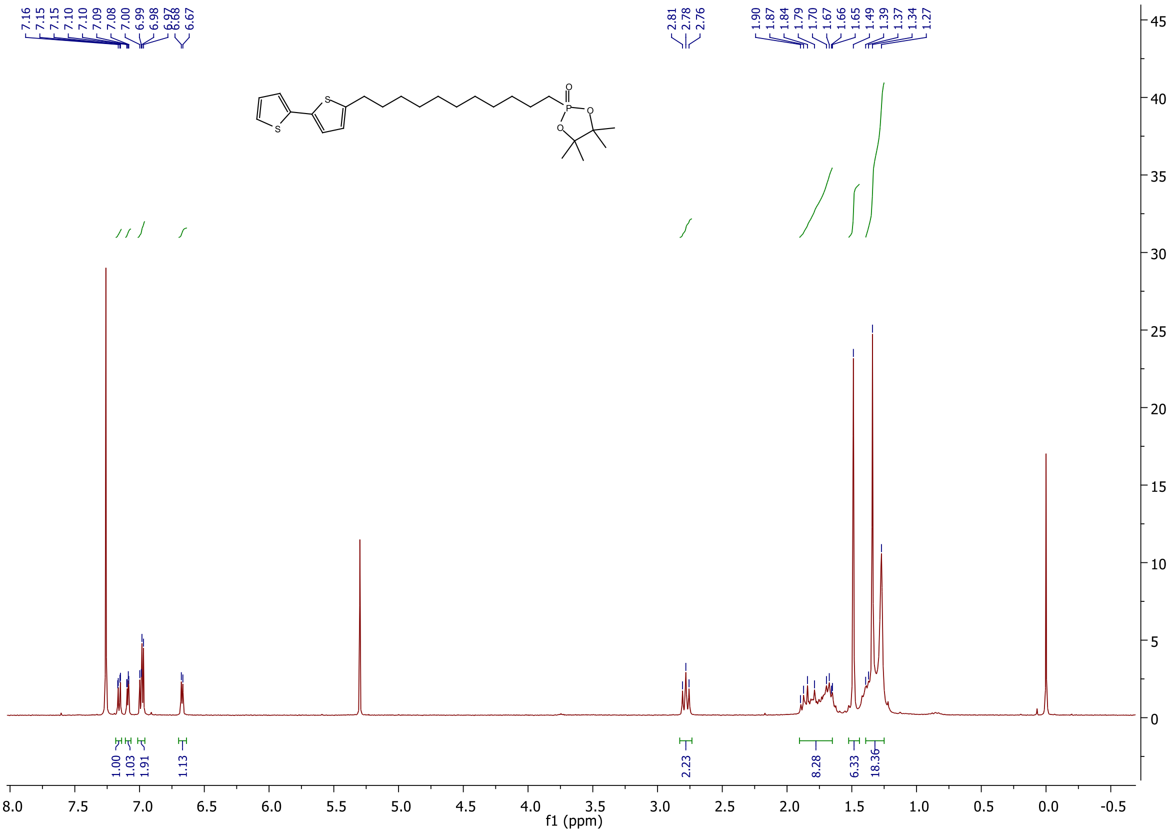

Figure S33. ^{1}H NMR (600 MHz, $CDCl_3$) of 2-[11-(2,2'-bithiophen-5-yl)undecyl]-4,4,5,5-tetramethyl-1,3,2-dioxaphospholane 2-oxide (**3d**).

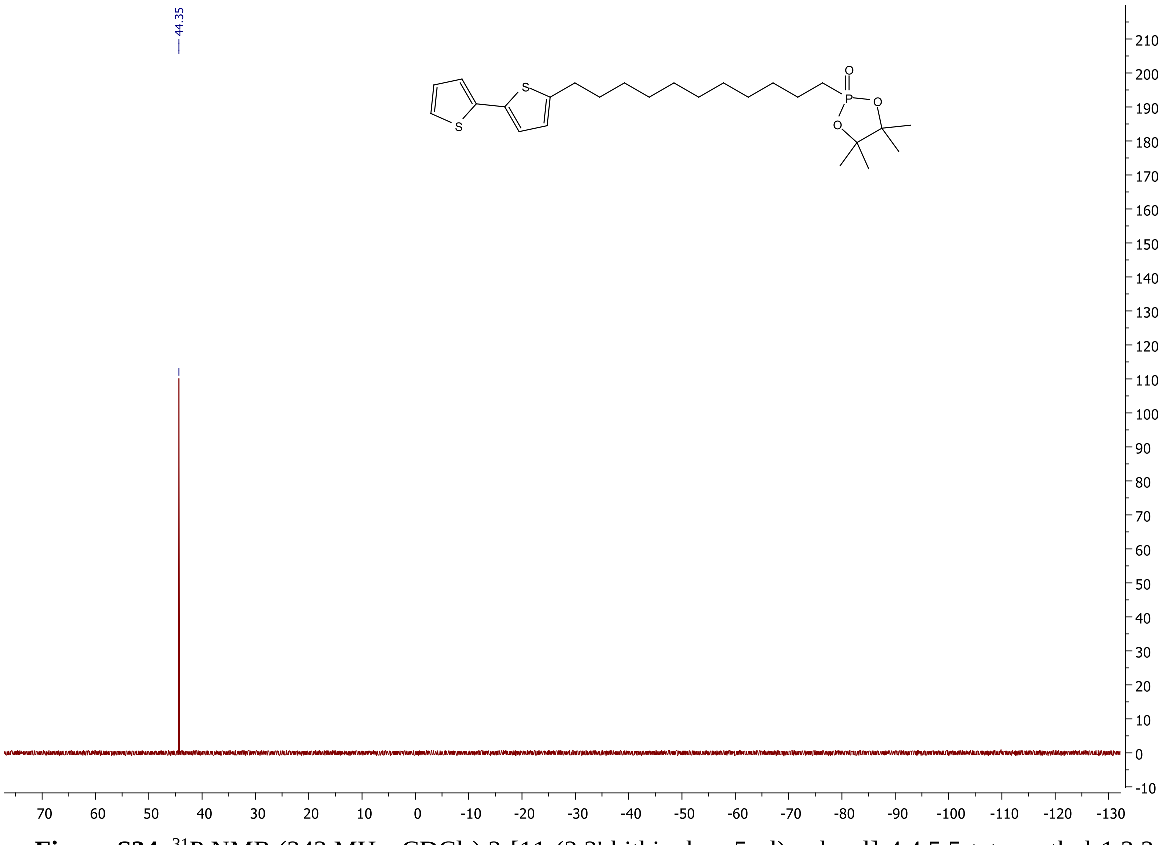

Figure S34. ^{31}P NMR (243 MHz, $CDCl_3$) 2-[11-(2,2'-bithiophen-5-yl)undecyl]-4,4,5,5-tetramethyl-1,3,2-dioxaphospholane 2-oxide (**3d**).

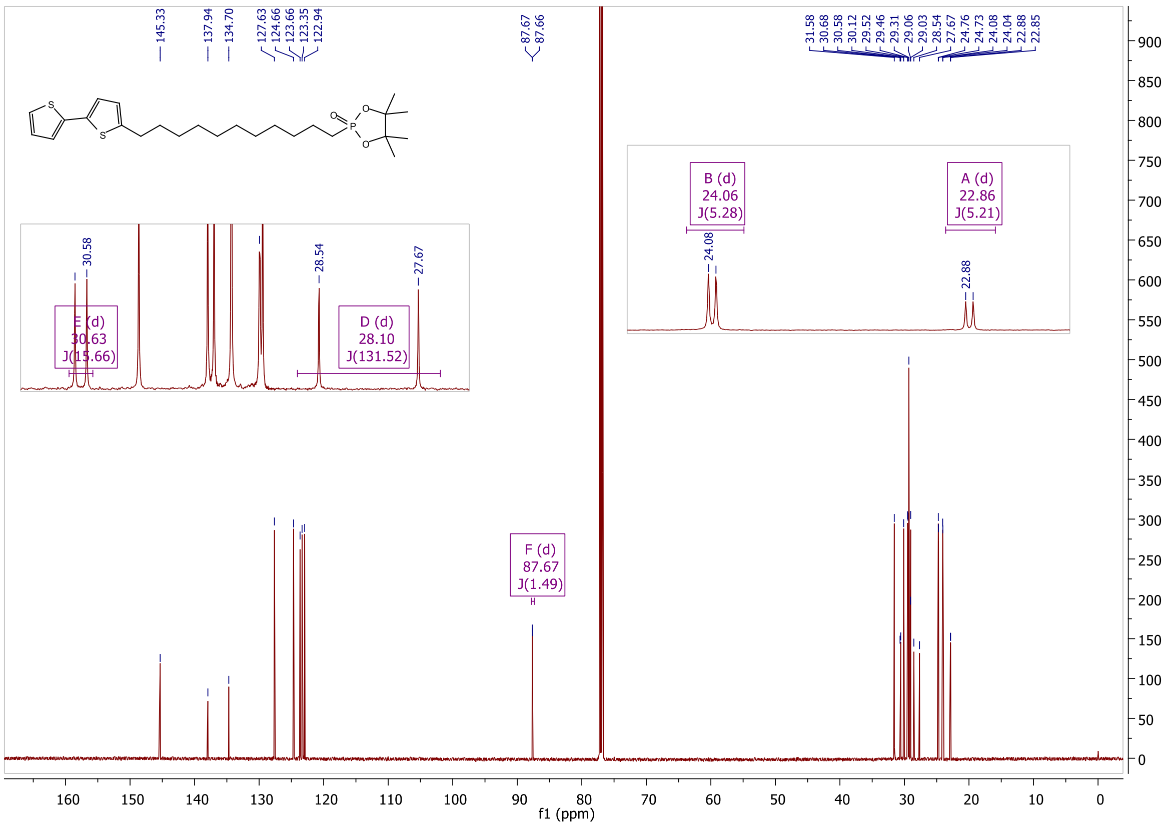

Figure S35. ^{13}C NMR (151 MHz, $CDCl_3$) 2-[11-(2,2'-bithiophen-5-yl)undecyl]-4,4,5,5-tetramethyl-1,3,2-dioxaphospholane 2-oxide (**3d**).

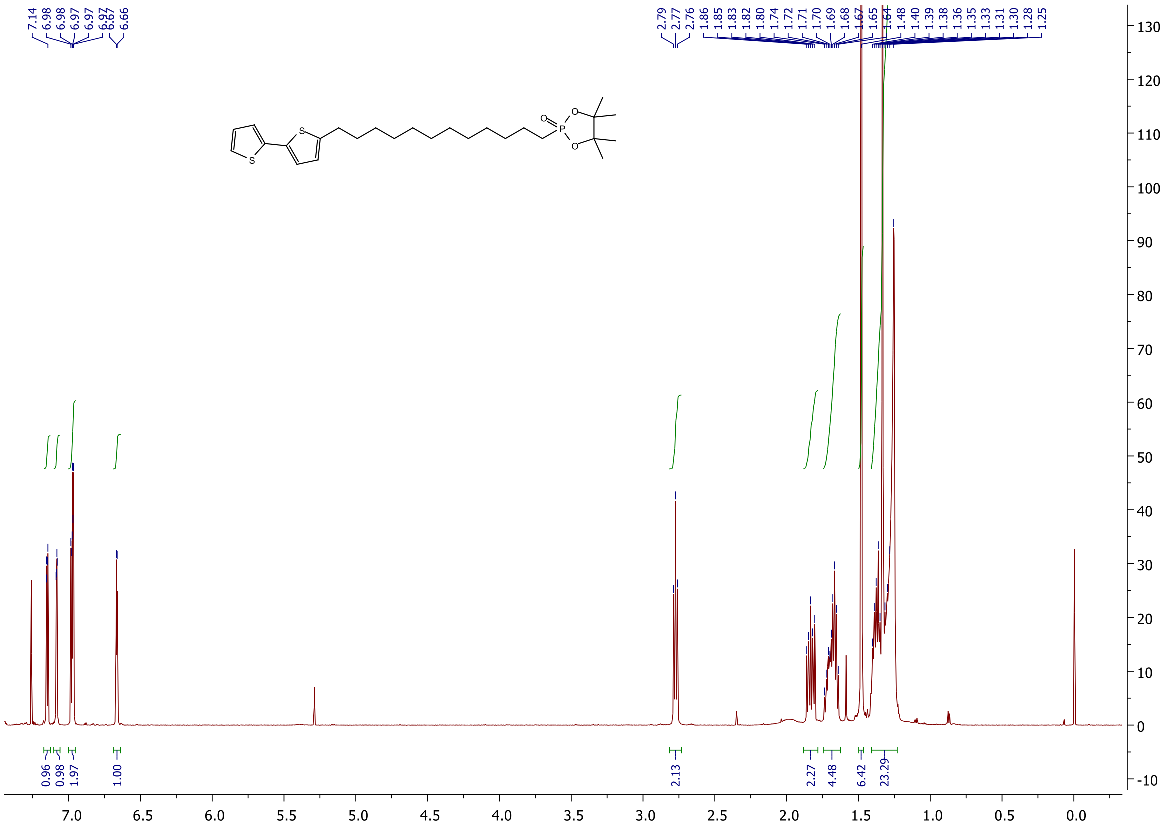

Figure S36. ^{1}H NMR (600 MHz, $CDCl_3$) of 2-[12-(2,2'-bithiophen-5-yl)dodecyl]-4,4,5,5-tetramethyl-1,3,2-dioxaphospholane 2-oxide (**3e**).

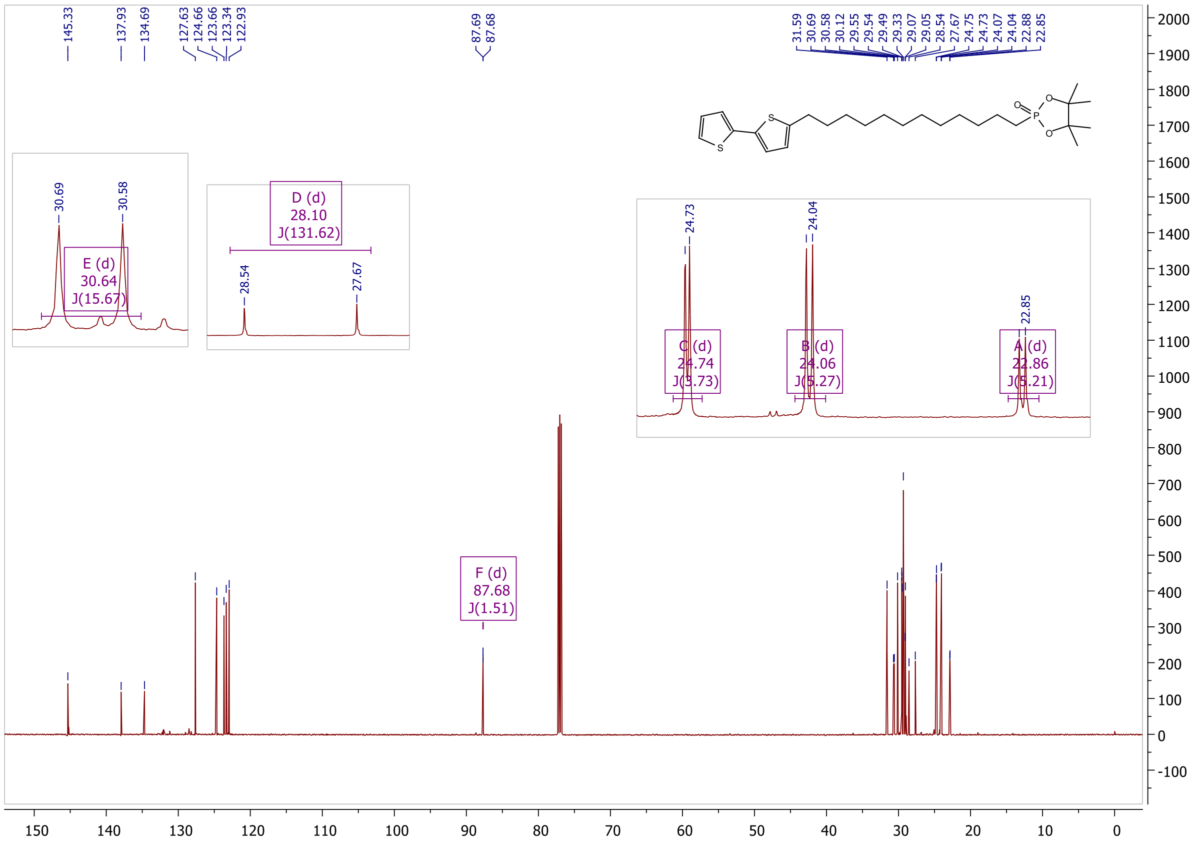

Figure S37. ^{13}C NMR (151 MHz, $CDCl_3$) 2-[12-(2,2'-bithiophen-5-yl)dodecyl]-4,4,5,5-tetramethyl-1,3,2-dioxaphospholane 2-oxide (**3e**).

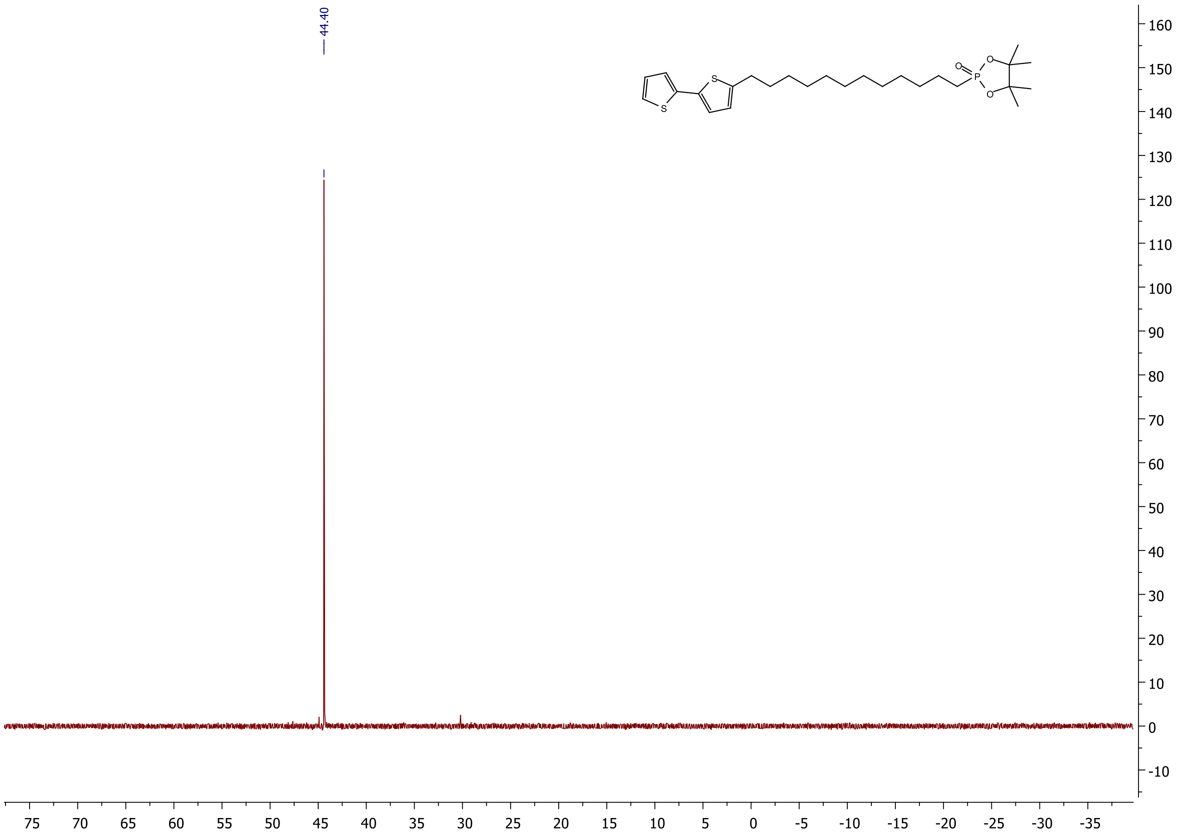

Figure S38. ^{31}P NMR (243 MHz, $CDCl_3$) 2-[12-(2,2'-bithiophen-5-yl)dodecyl]-4,4,5,5-tetramethyl-1,3,2-dioxaphospholane 2-oxide (**3e**).

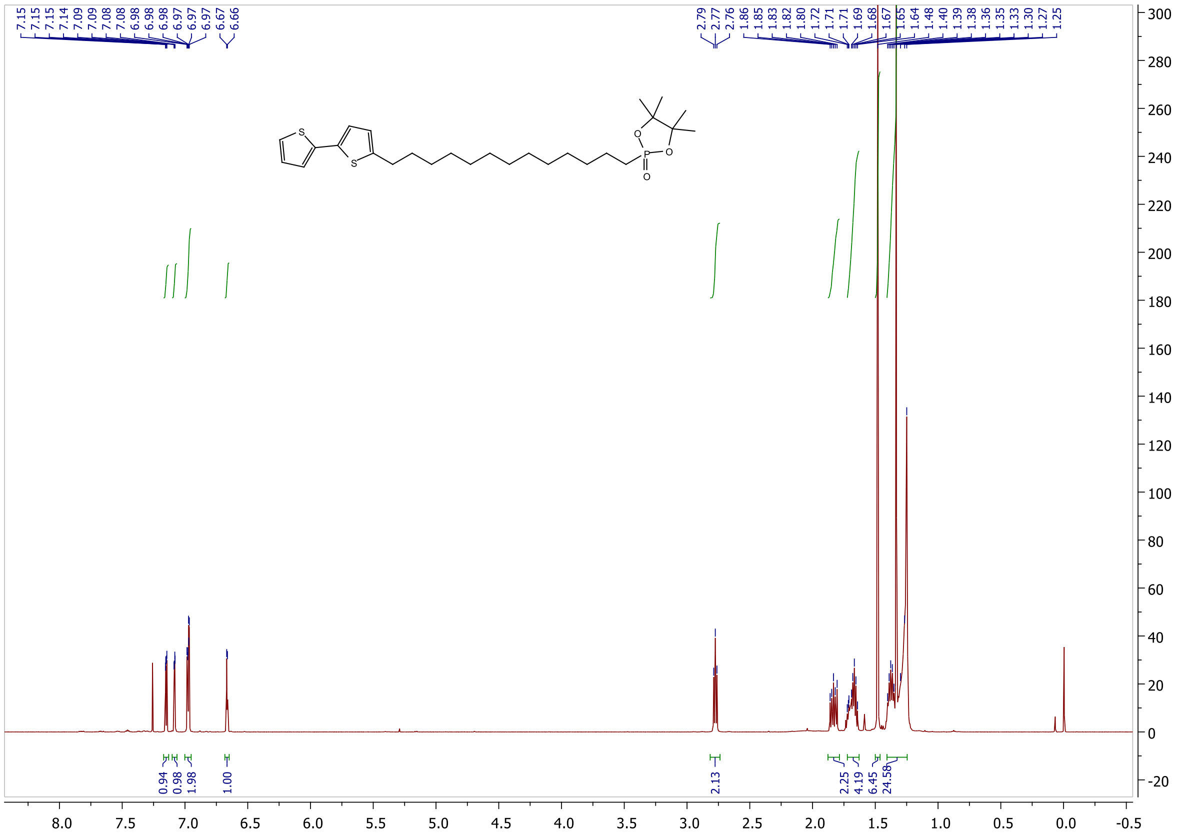

Figure S39. ^{1}H NMR (600 MHz, $CDCl_3$) of 2-[13-(2,2'-bithiophen-5-yl)tridecyl]-4,4,5,5-tetramethyl-1,3,2-dioxaphospholane 2-oxide (**3f**).

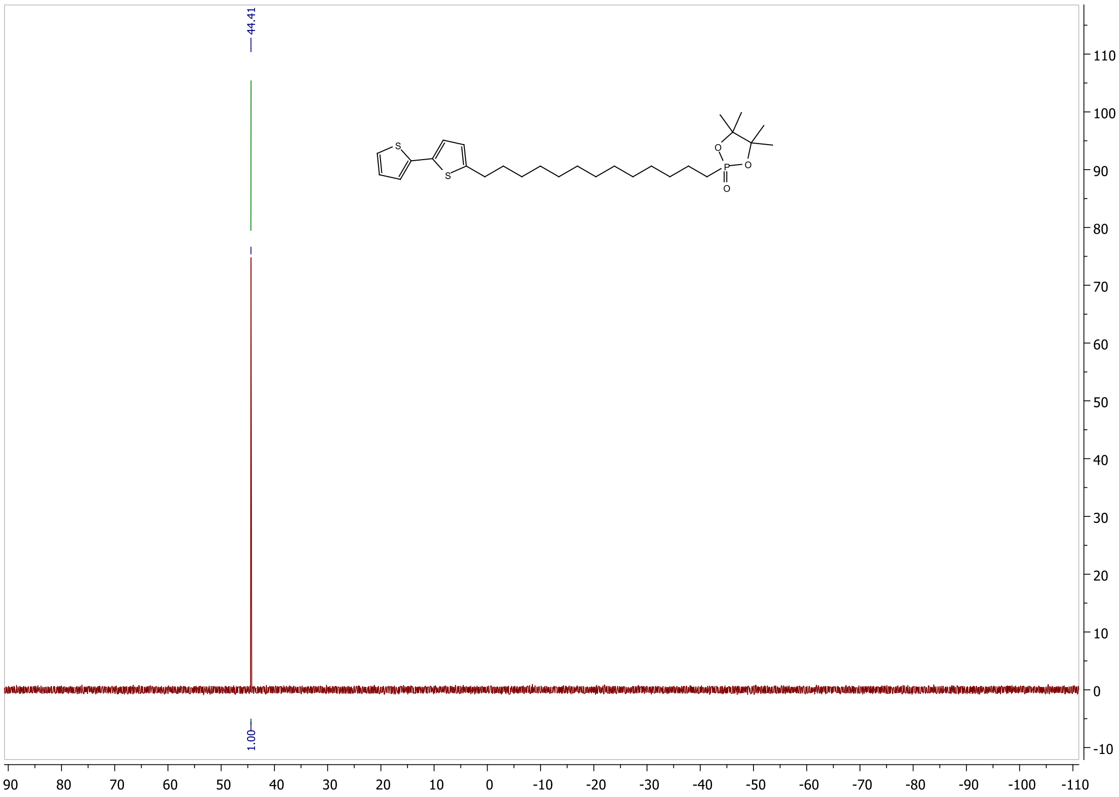

Figure S40. ^{31}P NMR (243 MHz, $CDCl_3$) 2-[13-(2,2'-bithiophen-5-yl)tridecyl]-4,4,5,5-tetramethyl-1,3,2-dioxaphospholane 2-oxide (**3f**).

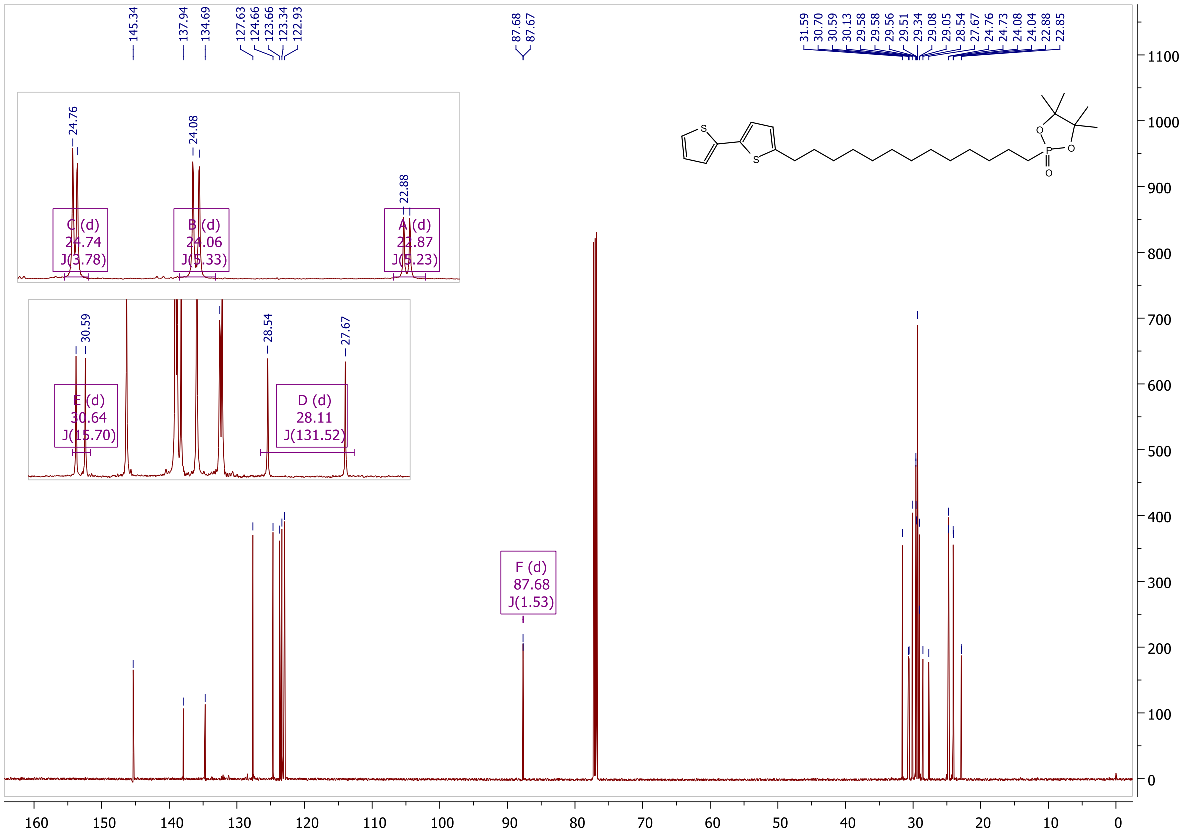

Figure S41. ^{13}C NMR (151 MHz, $CDCl_3$) 2-[13-(2,2'-bithiophen-5-yl)tridecyl]-4,4,5,5-tetramethyl-1,3,2-dioxaphospholane 2-oxide (**3f**).

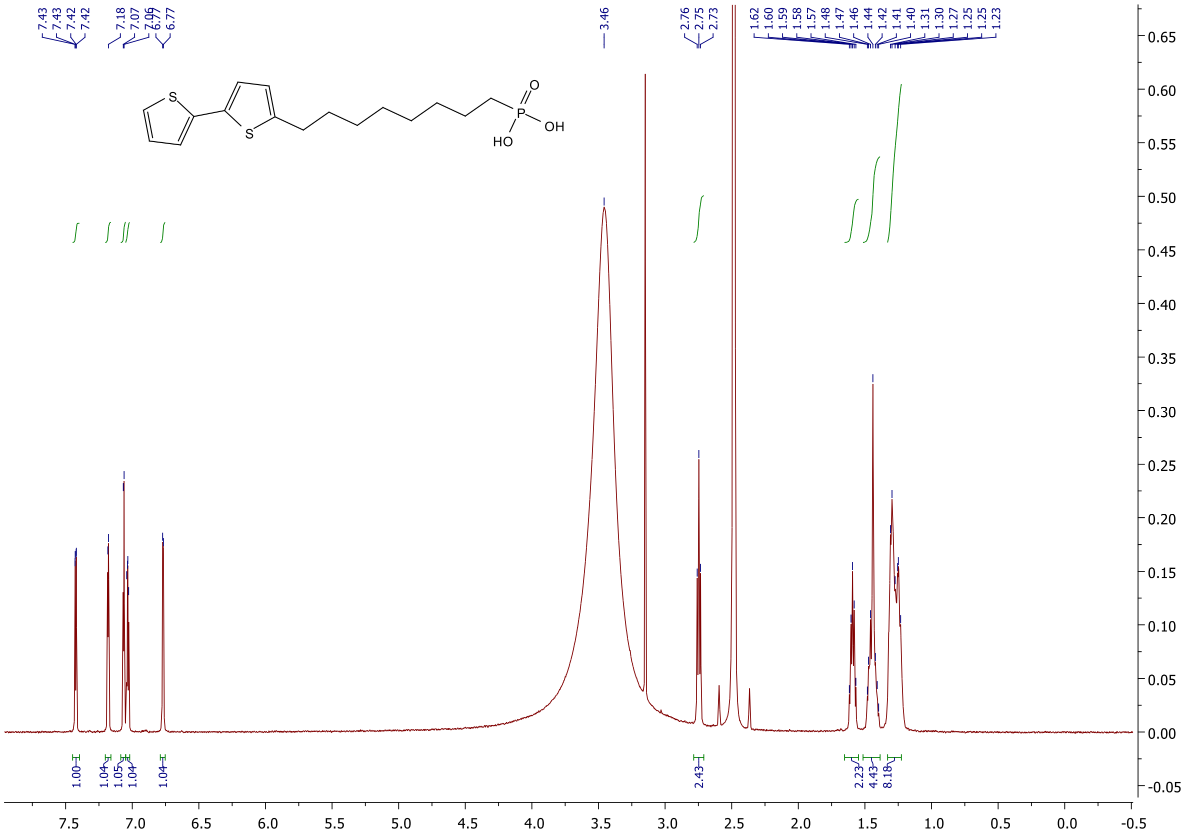

Figure S42. ^{1}H NMR (600 MHz, DMSO) of 8-(2,2'-bithiophen-5-yl)octylphosphonic acid (**TTC8P**).

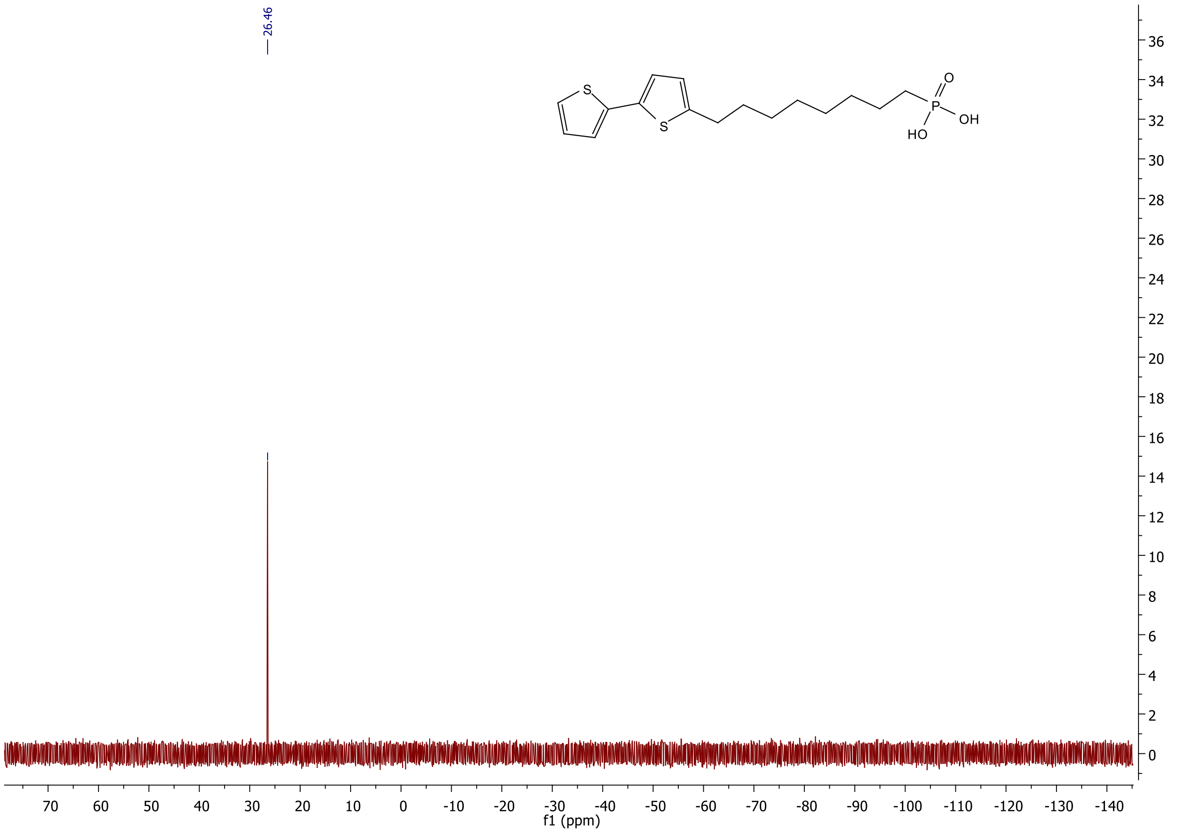

Figure S43. ^{31}P NMR (243 MHz, DMSO) of 8-(2,2'-bithiophen-5-yl)octylphosphonic acid (**TTC8P**).

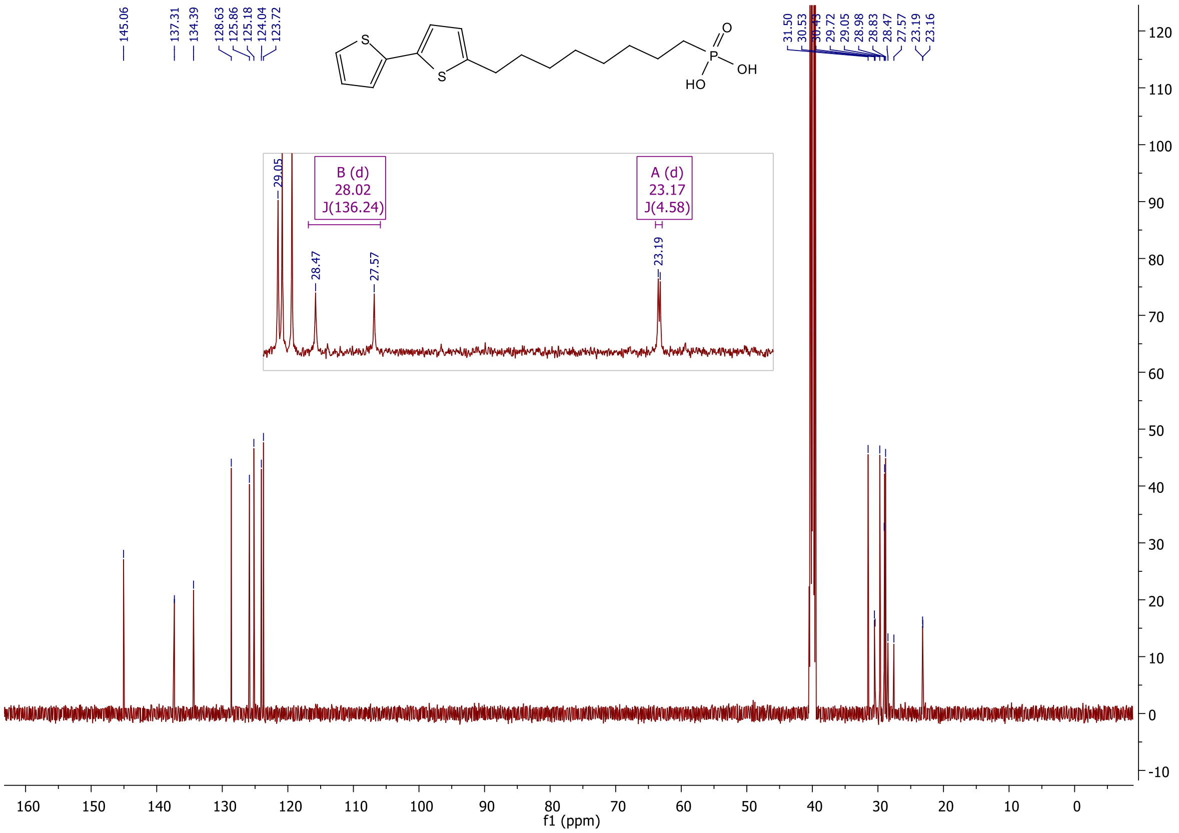

Figure S44. ^{13}C NMR (151 MHz, DMSO) of 8-(2,2'-bithiophen-5-yl)octylphosphonic acid (**TTC8P**).

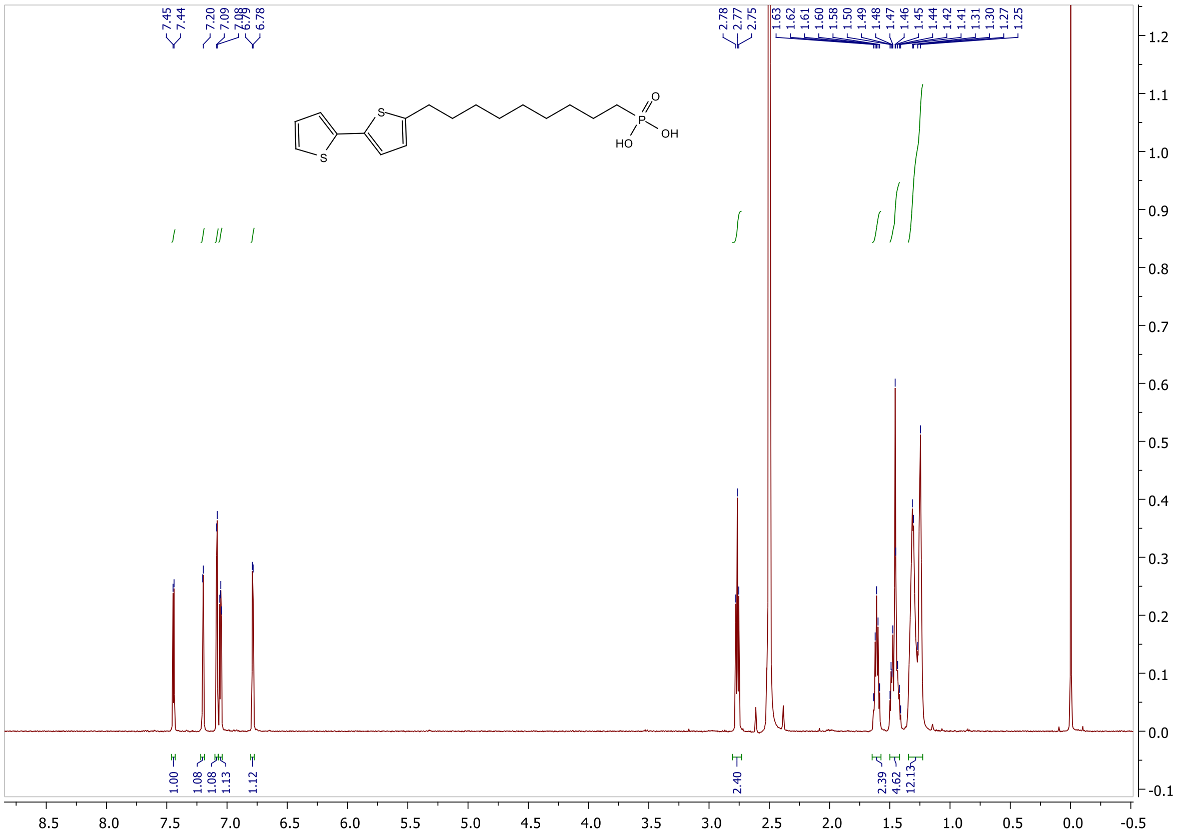

Figure S45. ^{1}H NMR (600 MHz, DMSO) of 9-(2,2'-bithiophen-5-yl)nonylphosphonic acid (**TTC9P**).

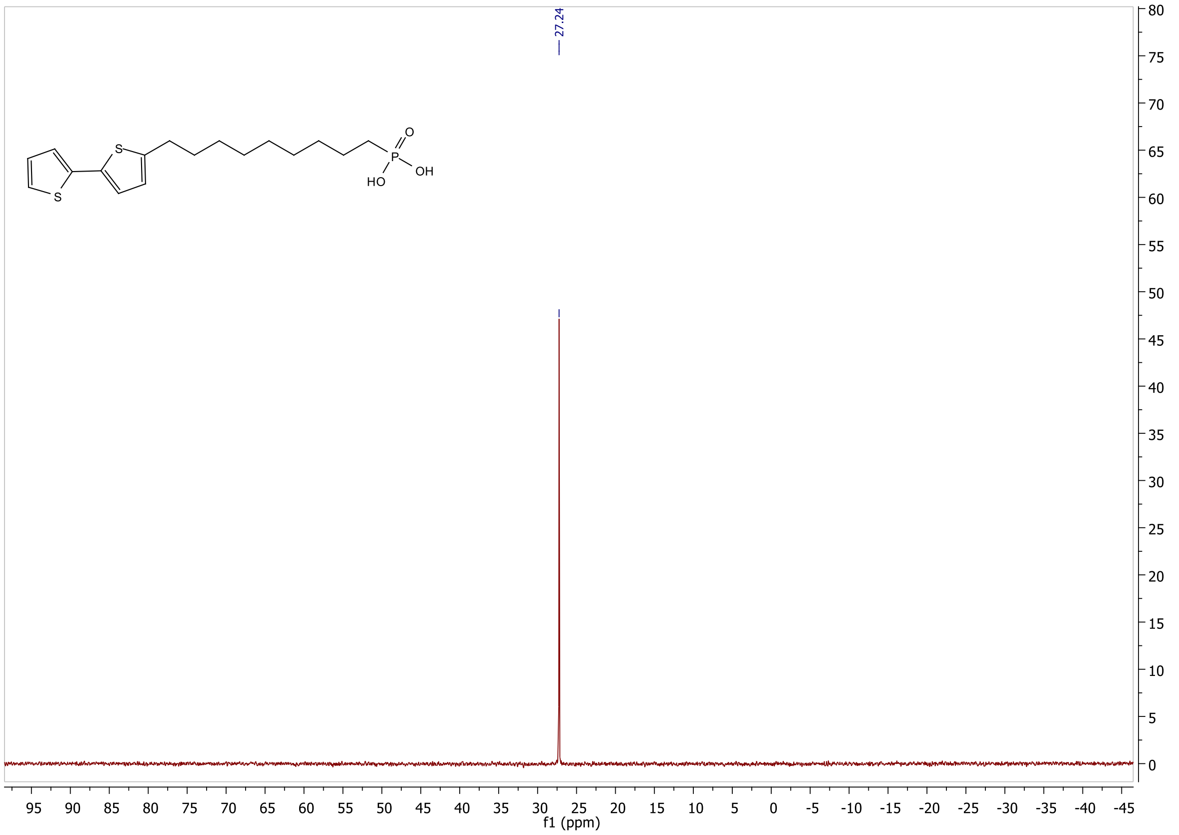

Figure S46. ^{31}P NMR (243 MHz, DMSO) of 9-(2,2'-bithiophen-5-yl)nonylphosphonic acid (**TTC9P**).

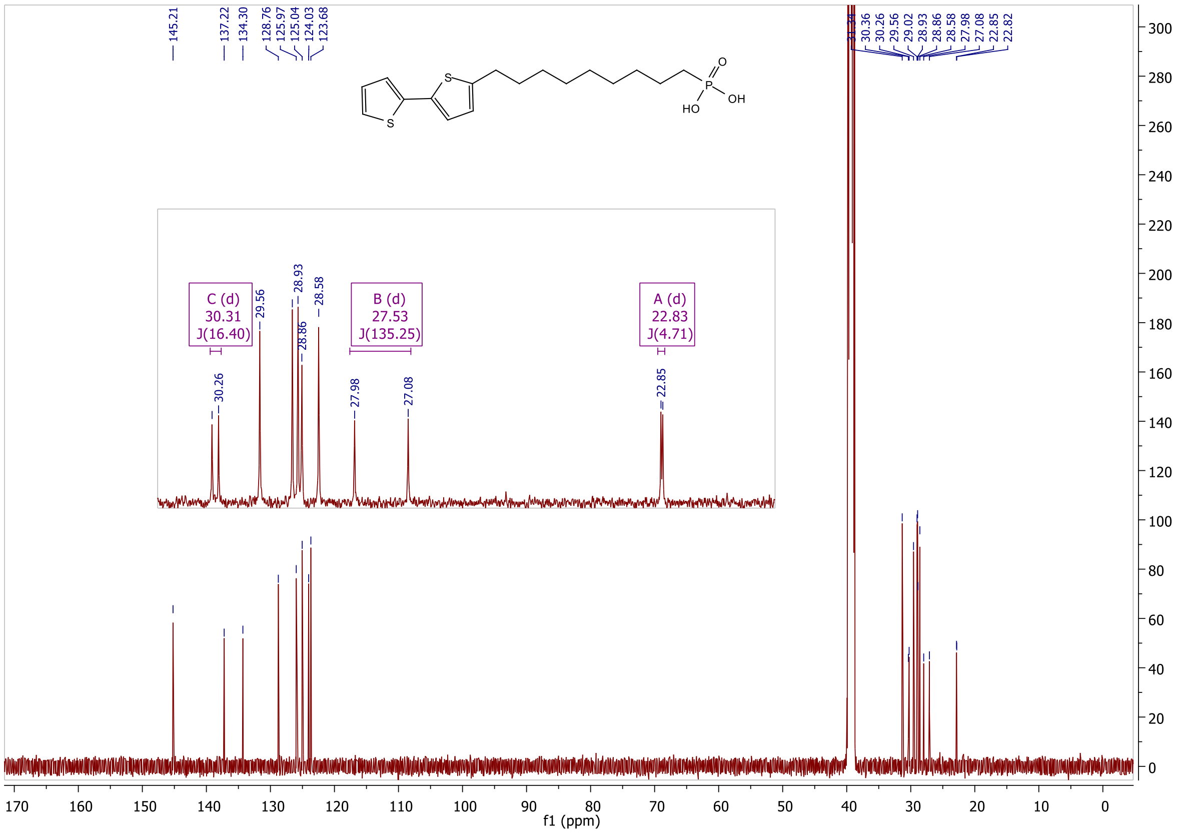

Figure S47. ^{13}C NMR (151 MHz, DMSO) of 9-(2,2'-bithiophen-5-yl)nonylphosphonic acid (**TTC9P**).

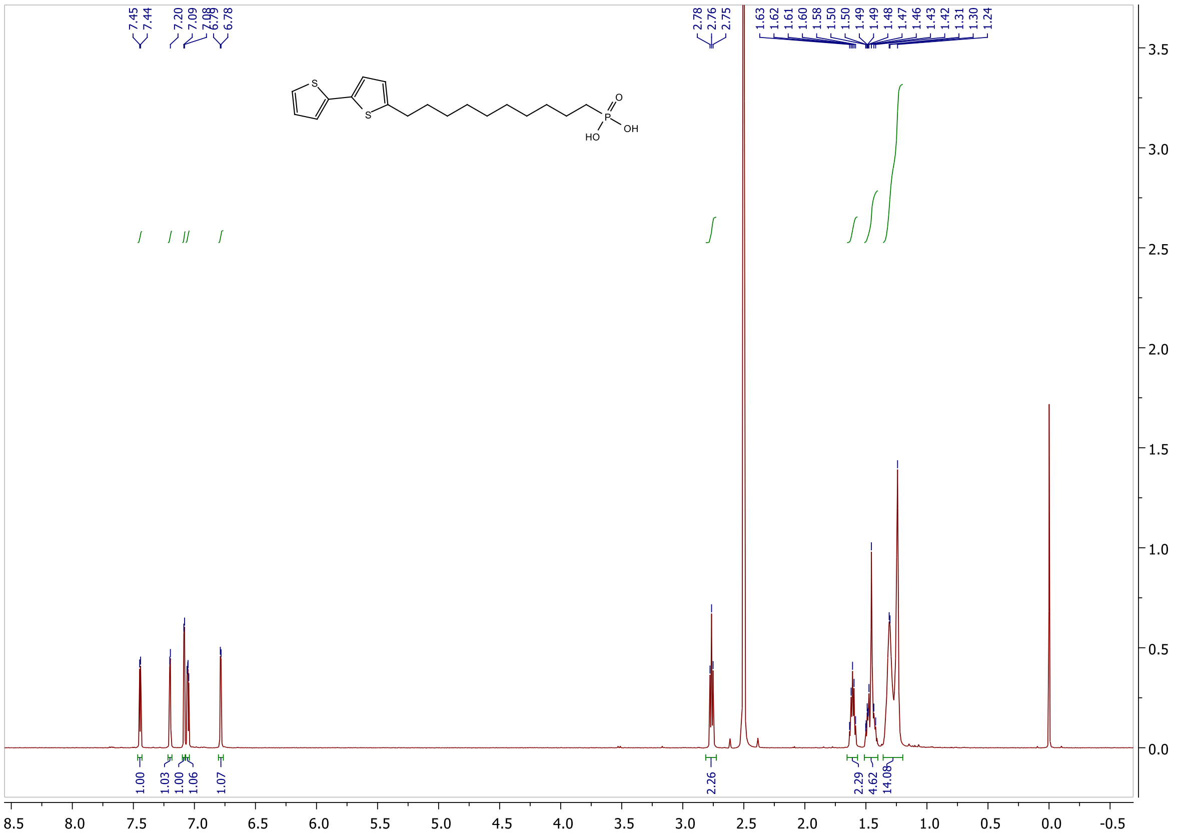

Figure S48. ^{1}H NMR (600 MHz, DMSO) of 10-(2,2'-bithiophen-5-yl)decylphosphonic acid (**TTC10P**).

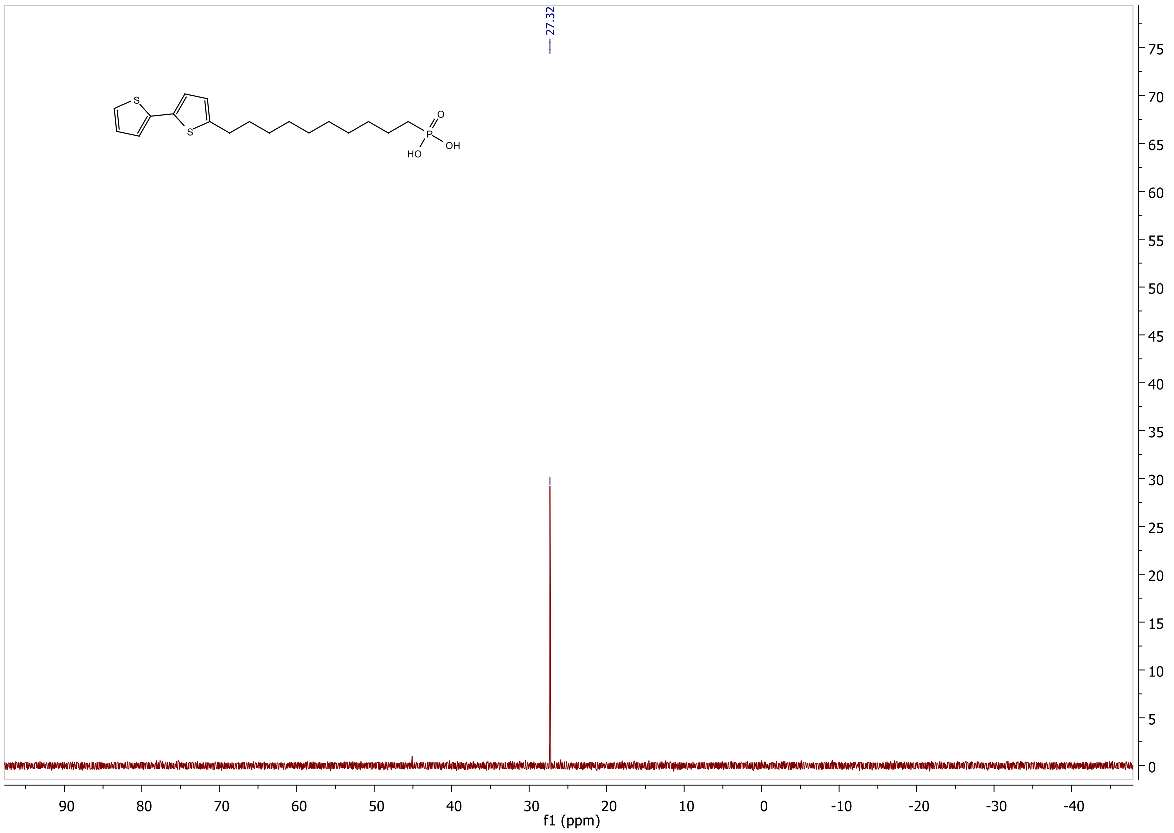

Figure S49. ^{31}P NMR (243 MHz, DMSO) of 10-(2,2'-bithiophen-5-yl)decylphosphonic acid (**TTC10P**).

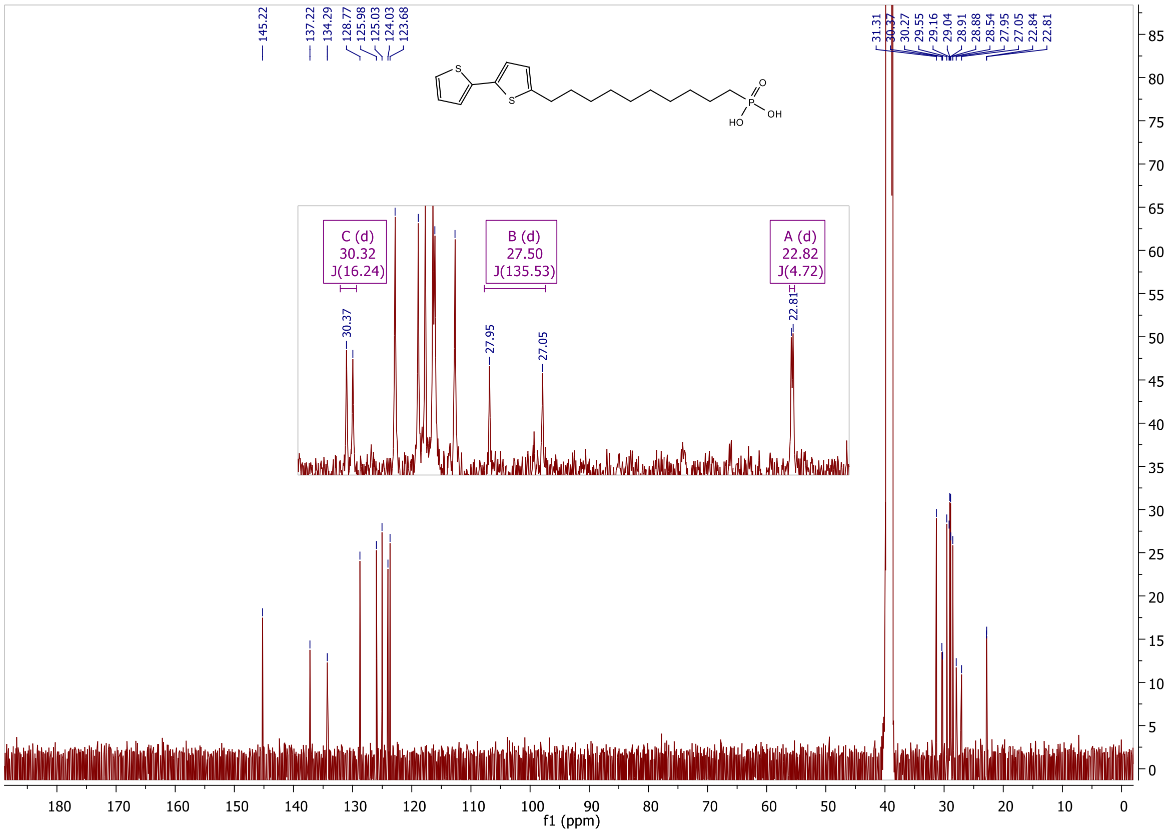

Figure S50. ^{13}C NMR (151 MHz, DMSO) of 10-(2,2'-bithiophen-5-yl)decylphosphonic acid (**TTC10P**).

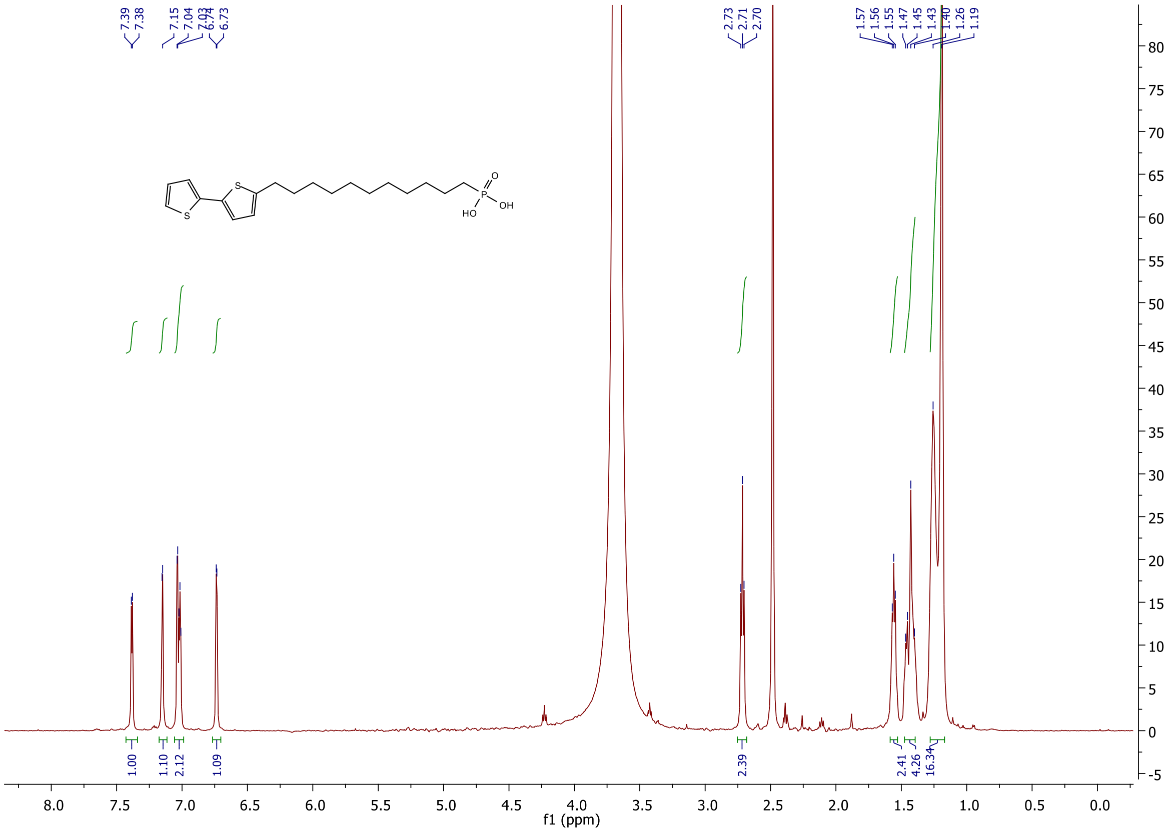

Figure S51. ^{1}H NMR (600 MHz, DMSO) of 11-(2,2'-bithiophen-5-yl)undecylphosphonic acid (**TTC11P**).

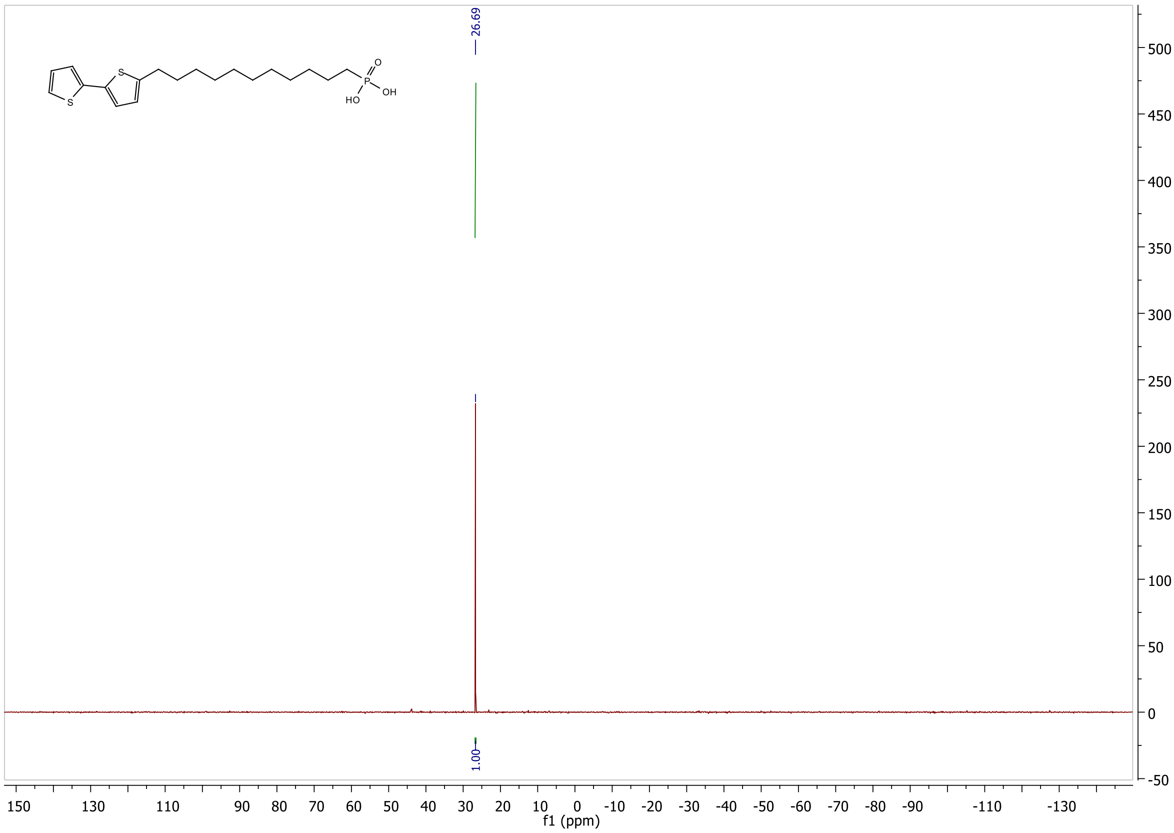

Figure S52. ^{31}P NMR (243 MHz, DMSO) of 11-(2,2'-bithiophen-5-yl)undecylphosphonic acid (**TTC11P**).

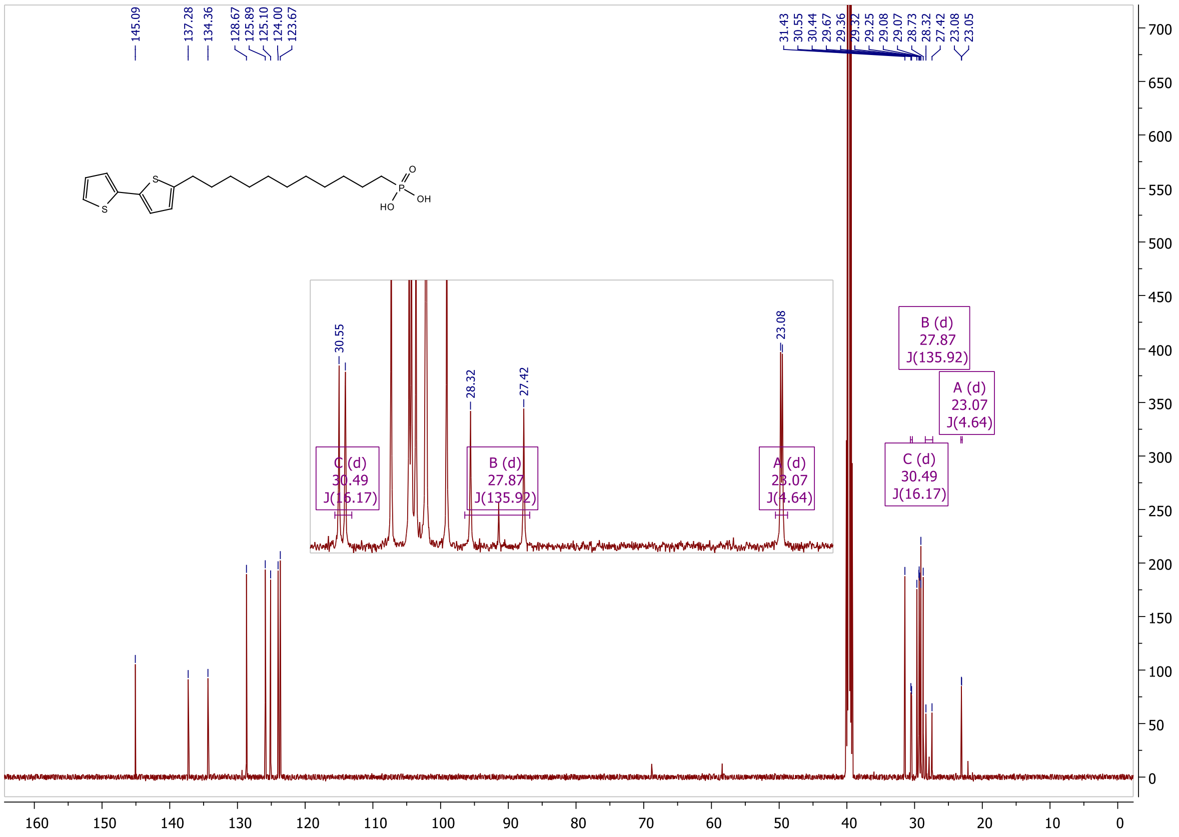

Figure S53. ^{13}C NMR (151 MHz, DMSO) of 11-(2,2'-bithiophen-5-yl)undecylphosphonic acid (**TTC11P**).

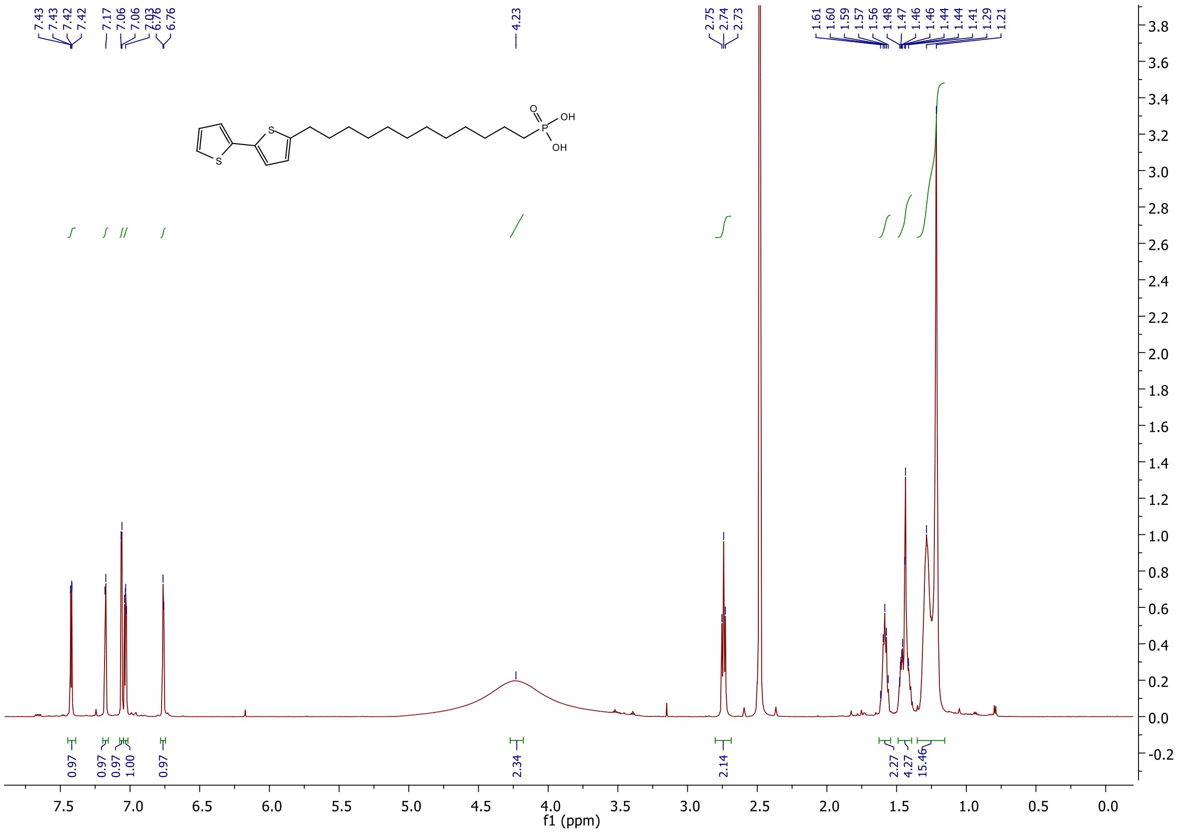

Figure S54. ^{1}H NMR (600 MHz, DMSO) of 12-(2,2'-bithiophen-5-yl)dodecylphosphonic acid (**TTC12P**).

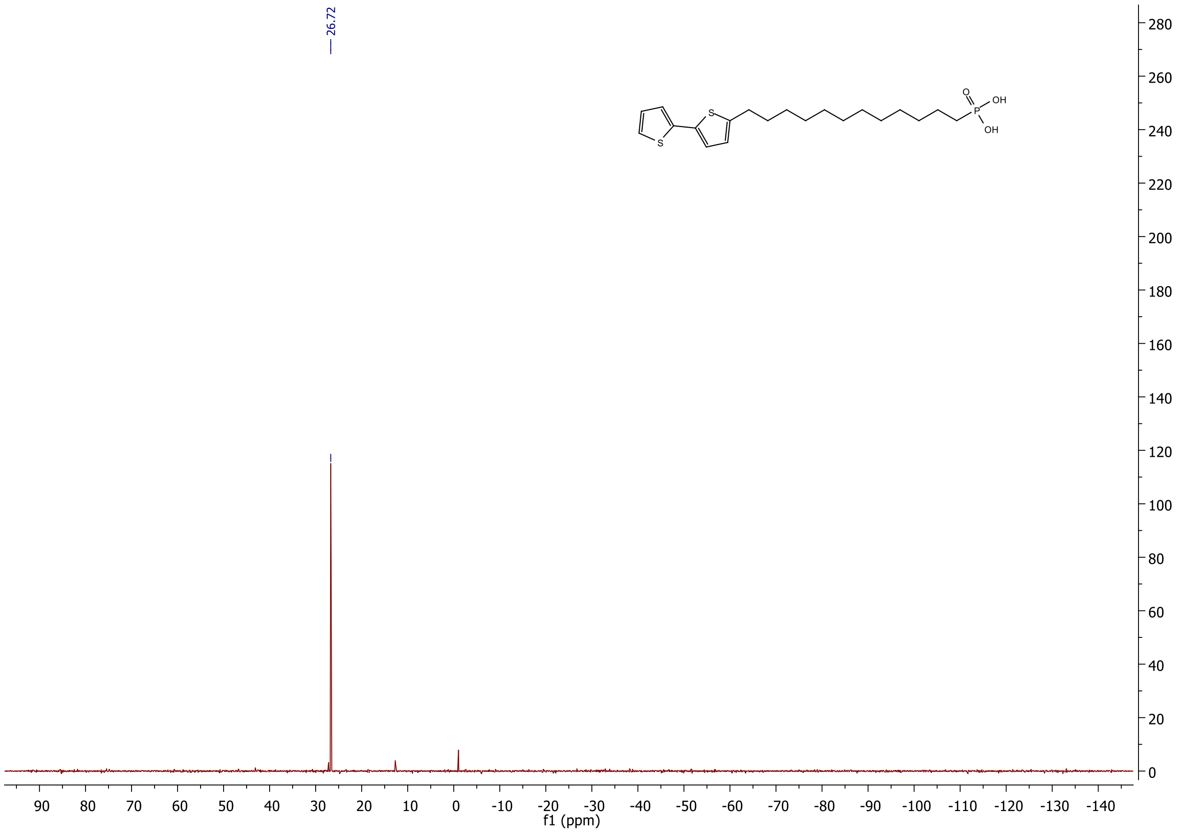

Figure S55. ^{31}P NMR (243 MHz, DMSO) of 12-(2,2'-bithiophen-5-yl)dodecylphosphonic acid (**TTC12P**).

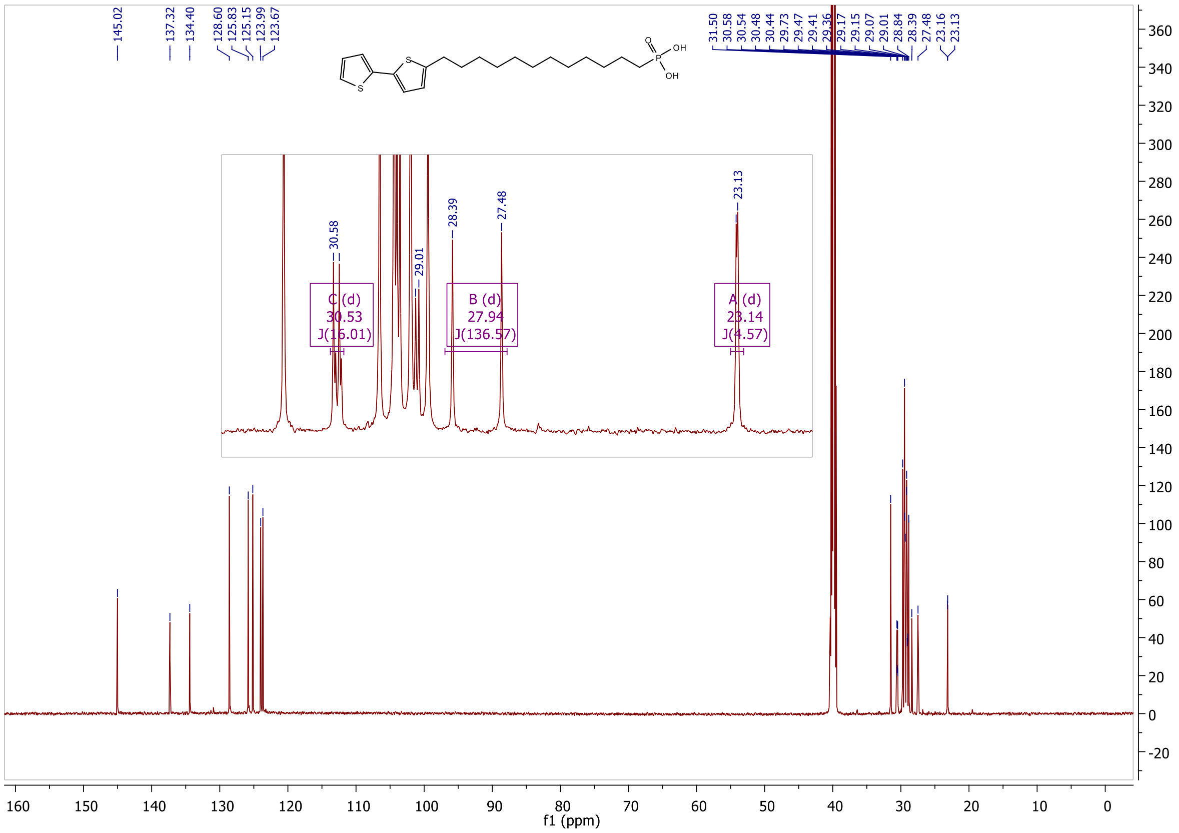

Figure S56. ^{13}C NMR (151 MHz, DMSO) of 12-(2,2'-bithiophen-5-yl)dodecylphosphonic acid (**TTC12P**).

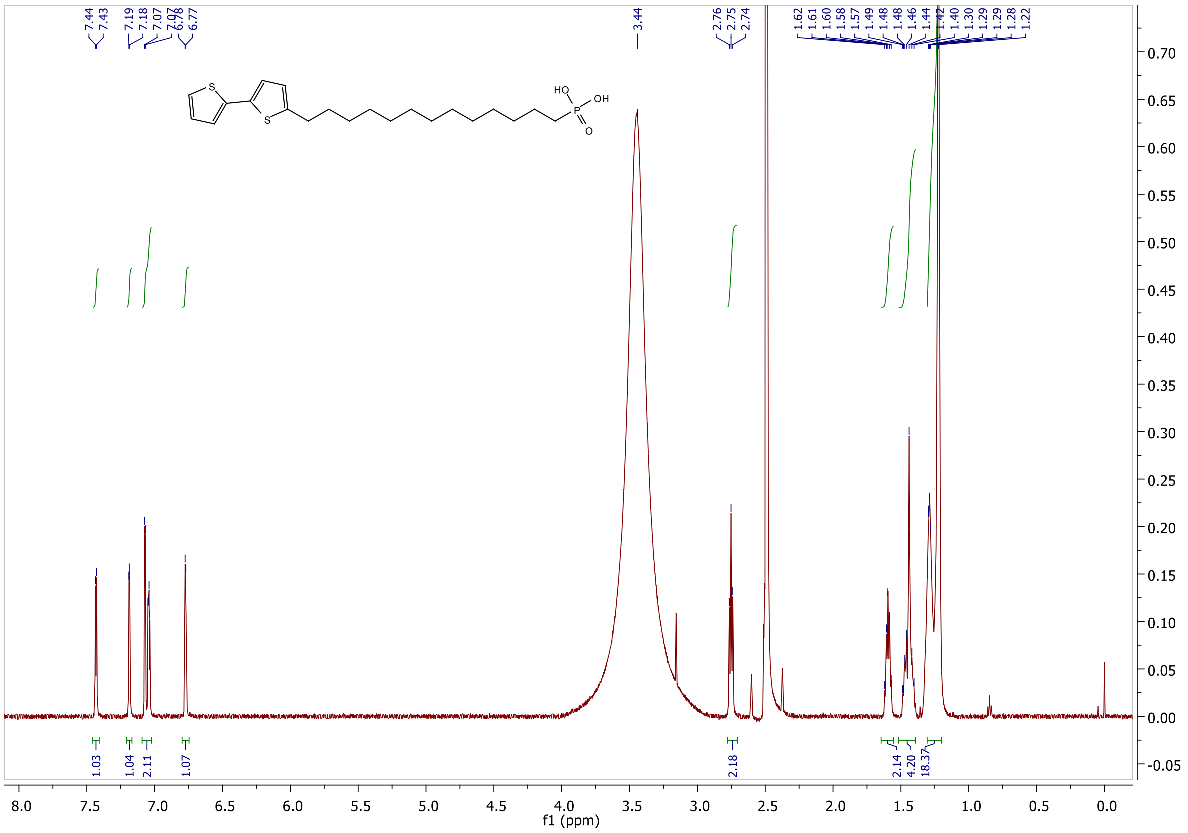

Figure S57. ^{1}H NMR (600 MHz, DMSO) of 13-(2,2'-bithiophen-5-yl)tridecylphosphonic acid (**TTC13P**).

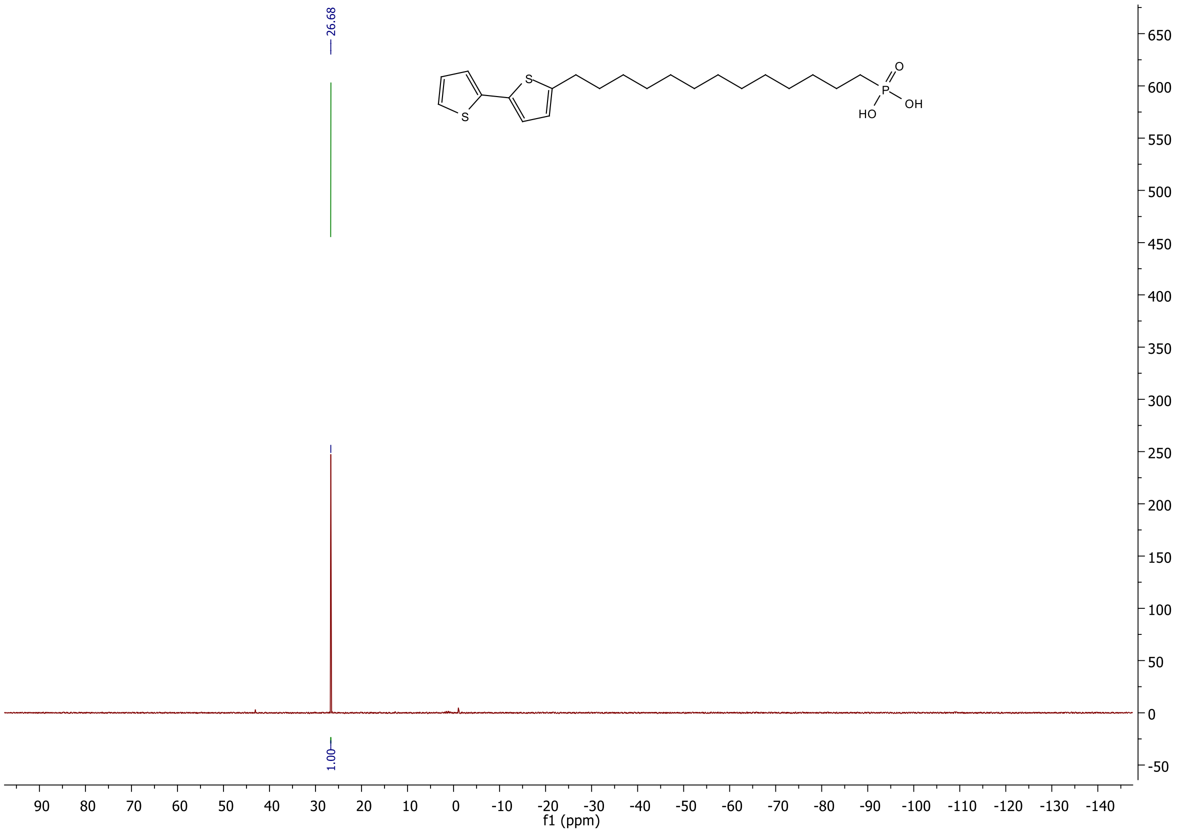

Figure S58. ^{31}P NMR (243 MHz, DMSO) of 13-(2,2'-bithiophen-5-yl)tridecylphosphonic acid (**TTC13P**).

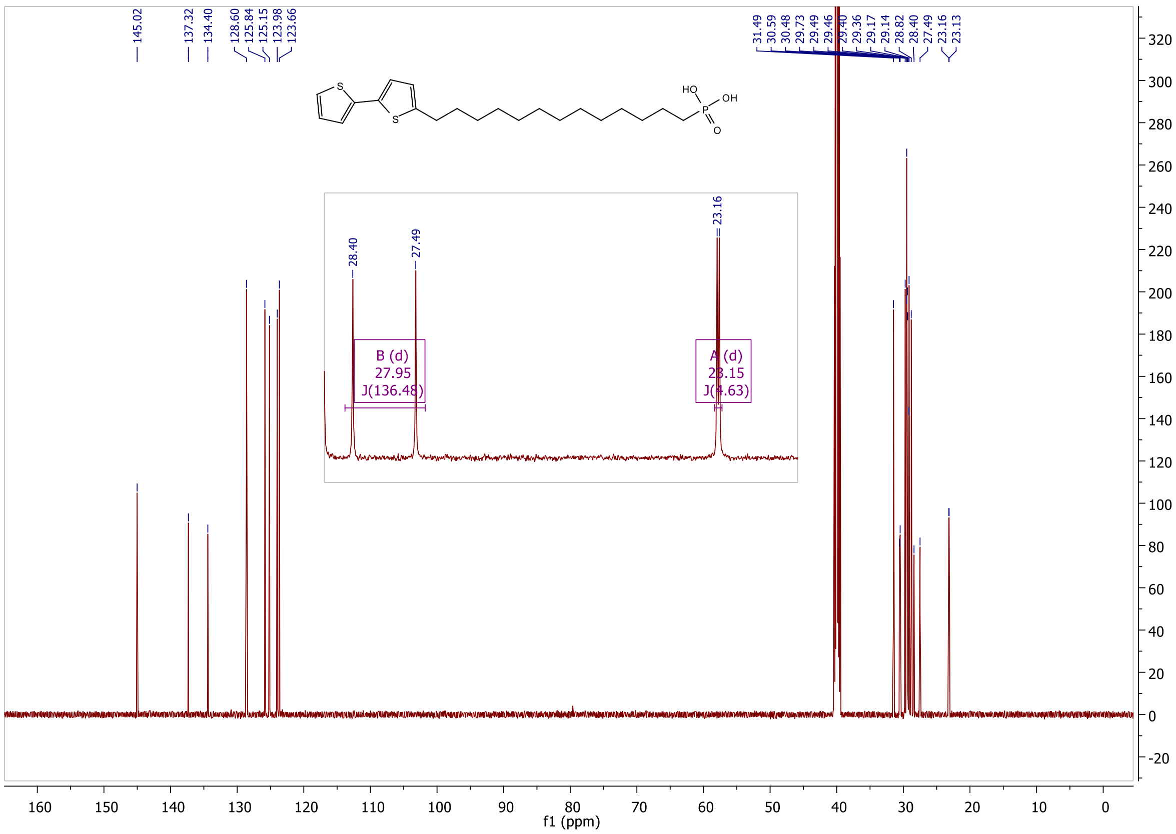

Figure S59. ^{13}C NMR (151 MHz, DMSO) of 13-(2,2'-bithiophen-5-yl)tridecylphosphonic acid (**TTC13P**).
